# Beyond Body Weight: Mapping Vitamin, Mineral, and Trace-Element Status in Anorexia Nervosa—Scoping Review

**DOI:** 10.3390/nu18203450

**Published:** 2026-10-21

**Authors:** James Chmiel, Donata Kurpas

**Affiliations:** 1 Faculty of Physical Culture and Health, Institute of Physical Culture Sciences, University of Szczecin, Al. Piastów 40B Blok 6, 71-065 Szczecin, Poland; 2 Division of Research Methodology, Department of Nursing, Faculty of Nursing and Midwifery, Wroclaw Medical University, 51-618 Wrocław, Poland

**Keywords:** anorexia nervosa, micronutrients, vitamins, minerals, trace elements, electrolytes, scoping review, PRISMA-ScR, refeeding syndrome, nutritional rehabilitation

## Abstract

**Introduction:** Background: Anorexia nervosa (AN) is associated with severe dietary restriction and multisystem effects of undernutrition, but biochemical micronutrient status does not necessarily parallel dietary intake or body weight. This scoping review mapped biological evidence on vitamin, mineral, trace-element, and electrolyte status in AN. **Methods:** The review was structured using JBI scoping review methodology and is reported according to PRISMA-ScR. PubMed/MEDLINE, Scopus, Web of Science Core Collection, and supplementary Google Scholar searching were used from inception to 30 March 2026, together with backward reference-list screening. Eligibility was defined using the Population–Concept–Context framework. Original human studies containing a clearly identifiable AN population and reporting biological micronutrient markers were eligible; refeeding studies, case reports/series, and diagnostically mixed cohorts were retained when relevant but were distinguished from AN-specific comparative or cohort evidence. Data were charted by design, population, biological matrix, timing, and nutrient outcome and synthesized narratively. **Results:** Database searching identified 621 records; 109 studies met eligibility criteria, and reference-list screening added 27 studies, yielding 136 included sources. Findings were heterogeneous and did not support universal biochemical depletion. Phosphorus was frequently preserved at admission but declined during refeeding in many cohorts. Potassium, sodium, and magnesium abnormalities were most evident in medically unstable, purging, or refeeding contexts. Serum calcium was commonly preserved despite impaired bone health. Iron deficiency was uncommon in many cohorts despite anemia and altered ferritin, transferrin, or hepcidin. Selenium, zinc, and vitamin D were recurrently low in several studies, whereas vitamin B12 was more often normal or elevated than deficient. **Conclusions:** AN is characterized by context-dependent micronutrient dysregulation rather than uniform deficiency. Interpretation should account for study design, illness stage, purging behavior, supplementation, refeeding, biological matrix, and the limitations of circulating biomarkers. Normal serum or plasma concentrations do not necessarily exclude depleted tissue stores, functional insufficiency, or refeeding risk. Interpretation is prioritized by study design, sample size, biological matrix and treatment stage rather than numerical direction-of-finding counts. This review maps monitoring-relevant abnormalities but does not establish the efficacy of supplementation strategies.

## 1. Introduction

Anorexia nervosa is a severe feeding and eating disorder characterized by persistent restriction of energy intake, resulting in significantly low body weight, together with intense fear of weight gain, persistent behaviors that prevent weight restoration, and a disturbed experience of body weight or shape [1,2,3,4]. Its core symptoms include progressive dietary restriction, rigid food rules, avoidance of meals, excessive preoccupation with calories, body weight, and appearance, compulsive exercise, and, in some patients, binge eating or purging [2,5,6]. Psychologically, anorexia nervosa is commonly associated with body image distortion, overvaluation of thinness, anxiety, depressive symptoms, irritability, social withdrawal, and reduced insight into the seriousness of malnutrition [1,4,7]. Somatic symptoms reflect starvation and multisystem undernutrition and may include marked weight loss, fatigue, cold intolerance, dizziness or syncope, bradycardia, hypotension, hypothermia, gastrointestinal complaints, amenorrhea or other endocrine disturbances, reduced bone mineral density, electrolyte abnormalities, and potentially life-threatening medical complications [5,6,8,9,10].

Micronutrients are biologically relevant to many of the organ systems affected in anorexia nervosa. Electrolytes and major minerals are central to neuromuscular excitability [11], cardiac conduction [12,13], renal regulation and acid–base balance [14,15], and refeeding safety [16], while trace elements such as zinc, selenium, and copper contribute to immune function, antioxidant defense, thyroid metabolism, erythropoiesis, connective-tissue integrity, and neurobiological regulation [17,18,19]. Vitamins are similarly important because they participate in bone metabolism, one-carbon metabolism, mitochondrial energy production, neurotransmitter synthesis, coagulation, antioxidant protection, collagen formation, and cellular proliferation [20,21,22,23,24,25]. Therefore, disturbances in micronutrient status may contribute to clinically relevant complications of anorexia nervosa, including impaired bone mineralization, anemia, muscle weakness, oxidative stress, immune vulnerability, neurological symptoms, and electrolyte instability.

Although weight loss and energy deficiency are the most visible clinical features of the disorder, anorexia nervosa also represents a state of profound biological adaptation to chronic undernutrition. These adaptations affect not only macronutrient metabolism, but also the absorption, transport, storage, redistribution, utilization, and excretion of vitamins, minerals, trace elements, and electrolytes. Consequently, micronutrient status in anorexia nervosa cannot be inferred reliably from body weight, dietary intake, or serum concentration alone.

However, the existing evidence does not support a simple model in which anorexia nervosa uniformly produces measurable biochemical depletion of all micronutrients. Several studies suggest that some nutrients are frequently abnormal, whereas others remain within reference ranges even in severely malnourished patients [26,27]. This apparent paradox may reflect the fact that circulating concentrations are influenced by multiple factors beyond intake, including reduced tissue compartments, dehydration, altered plasma volume, changes in carrier proteins, hepatic metabolism, endocrine adaptation, renal conservation or wasting, inflammation, supplementation, and stage of treatment [27,28,29,30]. Thus, apparently normal serum or plasma concentrations may coexist with depleted tissue stores or functional insufficiency. In contrast, elevated concentrations may sometimes reflect starvation-related redistribution, reduced binding capacity, or metabolic stress rather than adequate nutritional status [26,27,28,29,30].

Despite this relevance, the existing literature on micronutrient status in anorexia nervosa remains fragmented and difficult to interpret. Individual studies have assessed a wide range of nutrients, including fat-soluble vitamins, water-soluble vitamins, essential trace elements, major minerals, and electrolytes, but these studies differ substantially in their design, sample size, patient characteristics, illness severity, duration of illness, treatment setting, nutritional intake, supplementation status, laboratory methods, biological matrices, and comparison groups. Some studies report lower concentrations of selected micronutrients in patients with anorexia nervosa, whereas others show normal values, increased values, or no meaningful differences compared with healthy controls. This heterogeneity makes it difficult to determine whether anorexia nervosa is consistently associated with true biochemical deficiencies, whether abnormalities are nutrient-specific, or whether many observed differences reflect secondary effects of starvation physiology, hydration status, altered transport proteins, changes in body composition, reduced plasma volume, supplementation, or methodological variation.

A particular challenge in this field is that low dietary intake does not necessarily translate into low circulating micronutrient concentrations. In anorexia nervosa, serum or plasma values may be influenced by reduced total-body mass, altered protein binding, hepatic metabolism, inflammation, endocrine changes, dehydration, renal handling, gastrointestinal absorption, and compensatory redistribution between tissues and blood. Conversely, apparently normal circulating levels may not always exclude reduced tissue stores or functional insufficiency. This distinction is especially important for nutrients involved in bone health, erythropoiesis, mitochondrial metabolism, antioxidant defense, immune regulation, neurotransmitter synthesis, and neuromuscular excitability. Therefore, a synthesis limited only to whether a given nutrient is “low” or “normal” risks oversimplifying the biological complexity of anorexia nervosa. A broader mapping approach is needed to clarify what has actually been measured, how it has been measured, and what patterns can reasonably be inferred from the current evidence.

The topic is also clinically important because micronutrient assessment and supplementation are common concerns during nutritional rehabilitation. Patients with anorexia nervosa may be at risk of refeeding-related electrolyte shifts, impaired bone mineralization, anemia, oxidative stress, impaired immune function, and neurological complications. However, routine supplementation without clear evidence may obscure the interpretation of biochemical studies, while inconsistent reporting of supplementation status limits comparability across investigations. Moreover, some nutrients may appear paradoxically elevated rather than depleted, particularly when weight loss, reduced tissue compartments, altered carrier proteins, or metabolic adaptations affect circulating concentrations. As a result, clinicians and researchers need a clearer overview of which nutrients have been studied, which abnormalities appear most reproducible, and where the evidence remains inconclusive.

A scoping review is therefore particularly appropriate for this topic. Unlike a narrowly focused systematic review or meta-analysis, a scoping review can map a broad and heterogeneous body of evidence, identify the range of nutrients assessed, describe the populations and methods used, summarize the direction of reported findings, and highlight gaps that require more standardized future research. This approach is especially suitable because the literature includes diverse study designs, different laboratory markers, variable diagnostic criteria, and inconsistent reporting practices. Before stronger clinical conclusions can be drawn, it is necessary to understand the structure of the evidence base itself: which vitamins, minerals, trace elements, and electrolytes have been examined; which have been studied repeatedly; which are supported only by isolated findings; and which methodological limitations prevent firm interpretation.

The main objective of this scoping review is to systematically map and summarize the available evidence on vitamin, mineral, trace-element, and electrolyte status in individuals with anorexia nervosa. Specifically, the review aims to identify studies that measured circulating, urinary, functional, or other biological indicators of micronutrient status in patients with anorexia nervosa and to summarize the reported directions of findings relative to healthy controls, reference ranges, illness characteristics, or treatment-related changes, where available.

The review also aims to determine whether the current literature supports consistent patterns of deficiency, excess, or preserved biochemical status across specific nutrients. Rather than assuming that anorexia nervosa is uniformly associated with micronutrient depletion, this review seeks to evaluate the evidence nutrient by nutrient and to distinguish robust patterns from isolated or inconsistent observations. This is particularly important because the extracted evidence indicates that the findings are highly heterogeneous and that clear evidence for widespread biochemical deficiency may be limited. By presenting the results in a structured manner, the review will help clarify whether reported abnormalities are consistent across studies or vary by clinical context, measurement method, supplementation, illness severity, or other factors.

The review also seeks to provide a biological and clinical interpretation of the observed findings. For each nutrient or nutrient group, the review will consider plausible mechanisms linking anorexia nervosa with altered micronutrient status, including reduced intake, malabsorption, endocrine adaptation, changes in bone and muscle compartments, altered inflammatory or oxidative status, renal and gastrointestinal regulation, and effects of refeeding or supplementation. This mechanistic perspective is intended to avoid a purely descriptive interpretation and to place biochemical findings within the broader physiology of starvation, adaptation, and recovery.

## 2. Materials and Methods

This scoping review was revised to provide a transparent map of the evidence on biological micronutrient status in AN. The conduct and presentation were aligned with JBI methodological guidance for scoping reviews and the PRISMA Extension for Scoping Reviews (PRISMA-ScR). Reporting of the electronic search was additionally informed by PRISMA-S. A scoping review design was selected because the literature spans multiple nutrient classes, biological matrices, illness stages, age groups, and study designs and was therefore not expected to support a single pooled effect estimate. No protocol was prospectively registered; this is reported explicitly and considered in the limitations of the review.

### 2.1. Review Question and Population–Concept–Context Framework

The primary review question was: What biological evidence is available regarding vitamin, mineral, trace-element, and electrolyte status in individuals with AN, and how do reported abnormalities vary according to nutrient, biological matrix, clinical phenotype, treatment stage, and study design? Eligibility was operationalized using the Population–Concept–Context (PCC) framework. The Population comprised participants of any age or sex with AN diagnosed using contemporary or historical diagnostic criteria, or clearly identified by the original investigators as having AN. The Concept was biological micronutrient status, including measured concentrations, deficiency or excess according to study-specific reference ranges, functional biomarkers, urinary excretion, and clinically relevant changes during refeeding or nutritional rehabilitation. The Context was unrestricted and included inpatient, outpatient, community, and specialist eating-disorder settings in any country.

Because part of the literature combines AN with atypical AN, avoidant/restrictive food intake disorder (ARFID), bulimia nervosa, or other eating disorders, sources were distinguished in the revised synthesis by the directness of their AN evidence. Studies with AN-specific data or a separately extractable AN subgroup constituted the core evidence base. Mixed samples in which AN-specific micronutrient results were not fully separable, and studies focused only on atypical AN, were retained as contextual mapping evidence when they informed the scope of the field but were not used to make AN-specific prevalence claims or to override findings from AN-specific cohorts.

### 2.2. Eligibility Criteria

Original human studies were eligible when they (1) included participants with AN or a separately identifiable AN subgroup; (2) reported a quantitative biological measure of at least one vitamin, mineral, electrolyte, or trace element; and (3) provided data that could be interpreted in relation to AN at baseline, during acute illness, during refeeding/nutritional rehabilitation, or after weight restoration. Eligible biological matrices included serum, plasma, whole blood, erythrocytes, urine, hair, and validated functional or metabolite-based indices. Eligible designs included cross-sectional studies, case–control studies, prospective or retrospective cohorts, longitudinal clinical studies, interventional studies when eligible micronutrient data were reported, and case reports or case series describing uncommon but clinically informative abnormalities.

Studies were excluded when they were non-human; reviews, meta-analyses, editorials, or commentaries; conference abstracts without sufficient extractable data; did not contain a measurable biological micronutrient outcome; assessed dietary intake alone without a biological marker; contained no identifiable AN population; or reported only post-intervention values from which AN-related micronutrient status could not be interpreted. Publications in languages other than English were excluded at the full-text stage. When multiple publications described the same or overlapping cohort, reports were retained only when they contributed non-duplicate nutrient, subgroup, or time-point information; otherwise, the most complete report was prioritized.

### 2.3. Information Sources and Search Dates

PubMed/MEDLINE, Scopus, and Web of Science Core Collection were searched from database inception through 30 March 2026. Google Scholar was used as a supplementary discovery source to identify older, difficult-to-index, or potentially missed records. The reference lists of eligible studies and relevant reviews were screened manually for additional primary studies. No publication-year restriction was applied. Language was assessed during eligibility screening rather than used as an electronic search filter, which allowed the number of non-English full texts to be reported transparently in the study-selection flow.

### 2.4. Search Strategy and Limits of Reproducibility

The search combined three concept blocks: (1) AN; (2) micronutrients and individual nutrient names; and (3) biological status or measurement terms. Database-specific field tags and Boolean syntax were adapted to each platform. The nutrient block included broad terms (micronutrient*, vitamin*, mineral*, trace element*, electrolyte*) together with calcium, magnesium, phosphorus/phosphate, potassium, sodium, chloride, iron and iron-status markers, zinc, selenium, copper, manganese, vitamins A, B1, B2, B6, B9, B12, C, D, E, and K, and common biochemical synonyms. Biological-status terms included serum, plasma, blood, erythrocyte, urine/urinary, hair, concentration, level, status, deficiency, insufficiency, and biomarker. Appendix A presents illustrative database-specific strings and supplementary Google Scholar query families reconstructed retrospectively from the reported search concepts. The original platform search histories and verbatim queries were not preserved. Consequently, the Appendix A cannot establish the exact expressions, filters, platform settings, search sequence, or result sets used in the original searches and should not be interpreted as a fully reproducible or independently auditable account of search execution. Search reporting follows PRISMA-S principles.

Reference-list screening was conducted after full-text eligibility assessment. Candidate citations identified by backward citation searching were checked against the same eligibility criteria as database-derived records. These additional studies are reported separately from database searching in the study-selection flow to avoid conflating database records with records identified by other methods.

The 27 additional eligible sources identified by backward citation screening represented 19.9% of the 136 studies ultimately included. This substantial contribution raises the possibility that the electronic searches did not capture all relevant studies, for example because of differences in indexing or the terminology used in individual reports. However, the available extraction and citation-origin records do not identify the 27 sources individually as a traceable subset; consequently, their publication dates, indexing status, and distribution across nutrient categories cannot be assessed reliably. No claim is made that they were mainly older, poorly indexed, or confined to particular nutrients.

### 2.5. Study Selection

Duplicate citations were removed before screening. Selection then proceeded in two stages: title/abstract screening for obvious ineligibility, followed by full-text assessment against all eligibility criteria. At full text, eligibility was applied in a structured sequence covering population, biological micronutrient outcome, source type/design, timing of measurement, and language. For uncertain eligibility decisions, the reported process consisted of rechecking the full text against the stated criteria. The surviving records do not establish whether any disagreement was discussed or formally adjudicated by more than one reviewer. Full-text exclusions were assigned a single primary reason to permit transparent reporting in the PRISMA-style flow diagram. The available review records do not attribute title/abstract screening or full-text assessment to named individuals; nor do they document a second-reviewer verification step, independent duplicate screening, or a formal disagreement-resolution procedure. Consequently, we cannot reliably assign person-specific screening responsibilities or claim that decisions were reached by reviewer consensus.

At the full-text stage, each potentially eligible source was also classified as AN-specific/separately extractable or contextual mixed-sample evidence. Reports from overlapping cohorts were compared on sample characteristics, recruitment setting, dates where available, and outcome content to minimize double counting. Distinct reports from the same cohort could both be retained when they contributed different nutrients, time points, or clinically relevant analyses.

These reporting gaps relate to the review workflow rather than to the eligibility rules themselves. The study-selection steps and exclusions can be described from the available records, but the degree of independent verification and the identities of the decision-makers cannot be reconstructed with sufficient confidence for an author-level account.

### 2.6. Data Charting and Data Items

Data were charted into a structured evidence matrix and checked against the source publications. The charting framework was refined iteratively as recommended for scoping reviews. Extracted items included bibliographic information; study design; sample size and population characteristics; age and sex where reported; diagnostic group and AN subtype; comparator group; clinical setting; illness or treatment stage; exposure to refeeding or supplementation; nutrient or biochemical marker; biological matrix; assay or analytical method when reported; units and study-specific reference thresholds; central-tendency values; prevalence of low, normal, or elevated values; between-group or longitudinal differences; and reported associations with clinical variables.

The available source documentation likewise does not identify which author(s) initially charted individual study data, whether a second reviewer independently extracted or verified each entry, or how extraction discrepancies (if any) were resolved. The stated cross-checking against source publications should therefore be understood as a reported data-integrity activity, not evidence of documented independent double data extraction.

When a study reported multiple AN subtypes, age groups, biological matrices, or time points, these were charted separately whenever the publication allowed. Deficiency and insufficiency were defined according to the thresholds used by the original study rather than retrospectively harmonized, because assays, units, and reference ranges changed substantially across decades and laboratories. No unreported values were imputed.

### 2.7. Outcomes and Timing of Measurement

The primary outcome domain was biological micronutrient status in AN, encompassing vitamins, minerals, trace elements, and electrolytes. Outcomes included absolute concentrations, categorical deficiency/insufficiency or excess, comparison with healthy controls or laboratory reference intervals, urinary excretion or functional indices, and longitudinal changes. Secondary information included associations with body mass index, illness severity or duration, AN subtype, purging behavior, menstrual or endocrine status, bone health, hematological indices, inflammatory or metabolic markers, and treatment or weight-restoration status.

For interpretation, measurements were grouped conceptually into three clinical time windows whenever possible: (1) baseline/acute illness before substantial nutritional rehabilitation; (2) refeeding or active nutritional rehabilitation; and (3) post-restoration, remission, or follow-up. This distinction was applied in the revised synthesis because an electrolyte that is preserved at admission may behave differently during refeeding, and combining these stages can produce misleading conclusions.

For phosphorus, potassium, sodium, and magnesium, baseline status means the measurement obtained before meaningful nutritional restoration, with admission samples explicitly distinguished from samples already affected by intravenous fluid, phosphate/electrolyte replacement, or acute medical stabilization. Purging-associated losses and dehydration may be present at baseline but are not the same as chronic nutrient depletion from dietary restriction. New deficits detected after calories are reintroduced are labeled refeeding/emergent disturbances, rather than attributed retrospectively to the untreated baseline state. When source reports did not disclose sample timing or pre-analytic supplementation, stage was classified as uncertain rather than inferred.

### 2.8. Methodological Characterization and Interpretive Weighting

Formal risk-of-bias scoring was not used as an exclusion criterion because the objective was to map a broad evidence base rather than estimate an intervention effect; critical appraisal is optional in PRISMA-ScR and should be aligned with the purpose of the scoping review. Nevertheless, the editor’s concern regarding heterogeneous designs was addressed by explicitly stratifying sources according to design and AN specificity. Stratum A comprised AN-specific comparative studies and longitudinal/cohort studies, including mixed studies with separately extractable AN results. Stratum B comprised AN-specific uncontrolled clinical or cross-sectional cohorts. Stratum C comprised mixed eating-disorder or restrictive-eating-disorder samples in which AN-specific micronutrient results were only partly separable. Stratum D comprised case reports and case series.

This stratification is an interpretive framework rather than a numerical quality score. General statements about recurrent patterns, prevalence, or direction of findings were based primarily on strata A and B. Stratum C was used to provide contextual information and was not treated as direct AN-specific prevalence evidence. Stratum D was used to identify rare complications, extreme phenotypes, or hypothesis-generating observations and was not allowed to outweigh cohort-level findings.

When study findings conflicted, interpretive emphasis was guided first by directness of AN-specific data and comparability of the populations and measurement time points, then by study design (controlled or longitudinal evidence versus uncontrolled cross-sectional evidence), sample size, clinical setting and severity, AN subtype or purging behavior, supplementation and refeeding exposure, and the biological matrix and assay used. Larger, well-described AN-specific comparative or longitudinal cohorts were considered more informative for recurrent patterns than isolated small or diagnostically mixed samples. Visible methodological constraints, such as absent controls, selection bias, unclear assay methods, or unreported supplementation, were considered as reasons for caution. These were qualitative interpretive considerations only; no standardized risk-of-bias domains, formal certainty ratings, numerical study weights, or post hoc quality scores were applied.

### 2.9. Synthesis and Presentation of Results

Because studies differed markedly in diagnostic criteria, age, illness severity, treatment stage, biological matrix, laboratory assay, reference range, comparator, and statistical reporting, quantitative pooling was not considered appropriate. Results were therefore synthesized narratively and organized by nutrient. Within each nutrient, interpretation proceeded from study design and clinical timing to the direction and consistency of findings, with particular attention to whether abnormalities were present at baseline, emerged during refeeding, or persisted after weight restoration.

In practice, divergent results from studies differing in clinical setting or measurement window were described as potentially context-specific rather than pooled into a single direction. If comparable AN-specific studies remained discordant, the conclusion was presented as heterogeneous or inconclusive; direction-of-finding counts alone were not used to resolve the conflict. Case reports were reserved for uncommon manifestations and could not overturn patterns from AN-specific cohorts.

Synthesis is presented as an evidence-informed narrative rather than a tabulation of how many studies report low, normal, or high concentrations. We removed the unweighted direction counts formerly opening the 21 nutrient subsections, since one study may report several opposing results across clinical stages, diagnostic groups, or biological matrices. Evidence is interpreted using (in order) AN-specific extractability, admission versus refeeding versus follow-up sampling, comparative or longitudinal design, sample size, biological matrix, and clinical exposures (purging, fluid balance, supplements and treatment). Table 1 summarizes the pattern and its limitations; Supplementary Table S1 retains all 136 source-level entries. No numerical weighting, meta-analytic synthesis, or formal risk-of-bias assessment was performed.

### 2.10. Reference Management and Data Integrity

**Figure 1:**
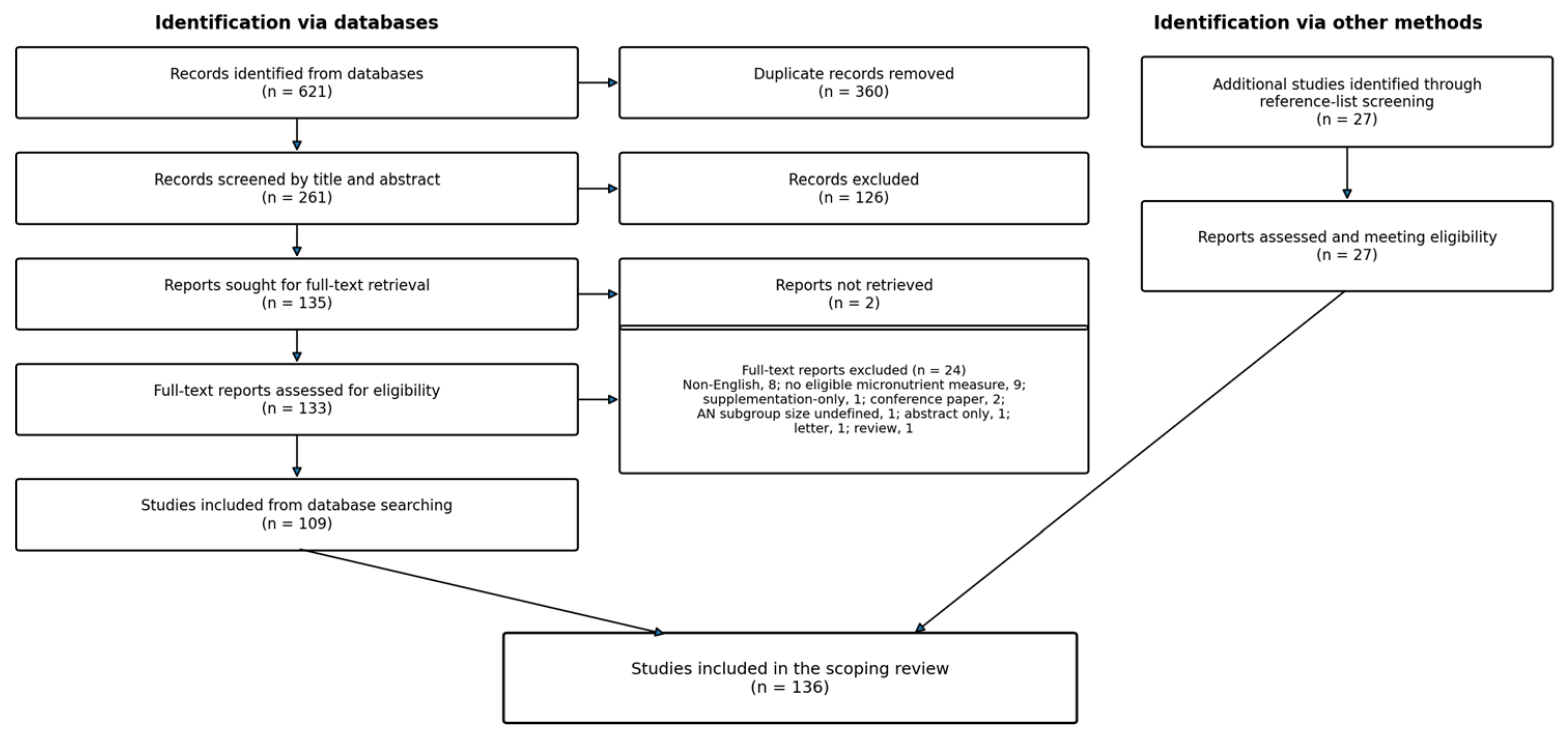
PRISMA-style flow diagram of study identification and selection. Database searching contributed 109 eligible studies; reference-list screening contributed 27 additional studies.

All included citations were checked against the original publications during preparation of the evidence table and narrative synthesis. Units, numerical values, study populations, and timing of measurement were cross-checked to reduce transcription error. Two reports sought for full-text assessment could not be retrieved and are reported separately as “not retrieved” in Figure 1. Illustrative reconstructed search strings (not the original execution histories) and a PRISMA-ScR reporting map are supplied in Appendix A and Appendix B, respectively.

## 3. Results

Figure 1 summarizes study selection. Database searching identified 621 records. After removal of 360 duplicates, 261 records underwent title/abstract screening and 126 were excluded. A total of 135 reports were sought for full-text retrieval; 2 reports could not be retrieved, leaving 133 full-text reports for eligibility assessment. Twenty-four full-text reports were excluded: eight were non-English, nine did not report an eligible micronutrient or vitamin measure, one reported only supplementation effects without an eligible status measure, two were conference papers, one did not define the number of participants with AN, one was available only as an abstract, one was a letter, and one was a review. Database searching therefore contributed 109 eligible studies. Backward reference-list screening identified 27 additional eligible studies, yielding 136 included sources in total [31,32,33,34,35,36,37,38,39,40,41,42,43,44,45,46,47,48,49,50,51,52,53,54,55,56,57,58,59,60,61,62,63,64,65,66,67,68,69,70,71,72,73,74,75,76,77,78,79,80,81,82,83,84,85,86,87,88,89,90,91,92,93,94,95,96,97,98,99,100,101,102,103,104,105,106,107,108,109,110,111,112,113,114,115,116,117,118,119,120,121,122,123,124,125,126,127,128,129,130,131,132,133,134,135,136,137,138,139,140,141,142,143,144,145,146,147,148,149,150,151,152,153,154,155,156,157,158,159,160,161,162,163,164,165,166].

Reference-list additions accounted for 27/136 included studies (19.9%). The study-level route of identification was not retained in a form allowing the citation-derived subset to be separately characterized by publication year, indexing, or nutrient; these subgroup characteristics therefore remain undetermined.

The concise Table 1 presents nutrient-level evidence types, principal patterns and key limitations. The original detailed evidence matrix has been relocated without removing any of its 136 study entries to Supplementary Table S1; it continues to document the individual populations, designs, biological measures and reported findings underlying this narrative synthesis.

The included evidence comprised AN-specific comparative and longitudinal cohorts, uncontrolled clinical cohorts, mixed eating-disorder cohorts, and case reports or case series. These designs were not treated as methodologically equivalent. General statements about the direction and frequency of micronutrient abnormalities were based primarily on AN-specific or separately extractable comparative/cohort evidence (strata A–B). Mixed samples with incompletely separable AN results and case reports/series (strata C–D) were used mainly for contextual, sentinel, or hypothesis-generating observations.

### 3.1. Phosphorus

Interpretive summary (AN-specific cohorts, admission versus treatment): Admission serum/plasma phosphate was commonly within laboratory limits in the 153-patient baseline study [31], whereas retrospective inpatient cohorts observed a new phosphate decline during early refeeding (99 patients [32]; hospitalized adolescents and young adults [33]). Repeated measurements and clinical stage, not the number of reports classed as low, carry the main inference. Baseline and nadir values are not interchangeable.

Phosphorus findings were strongly stage-dependent: admission values were often preserved, whereas hypophosphatemia frequently emerged during refeeding. For example, study [31] reported phosphorus deficiency in only 3.5% of adult female patients with anorexia nervosa, even though nearly half of the cohort had at least one micronutrient or vitamin deficiency. Similarly, study [78] found a mean admission phosphorus level of 4.2 mg/dL, with admission hypophosphatemia in only 4.2% of pediatric and adolescent patients, whereas study [82] reported that phosphorus was normal on admission in 84% of severely ill adult inpatients.

Refeeding cohorts consistently reported new-onset hypophosphatemia despite often-preserved admission values: 21.2% of admissions in [32], 28.7% of 300 adolescents and young adults in [33], and 33.3% of severely ill women in [34]. In a combined AN/ARFID cohort, more than one-third developed hypophosphatemia during hospitalization [83]. These estimates reflect different populations and definitions rather than a pooled prevalence.

Phosphate nadirs generally occurred early during nutritional rehabilitation. In [79], 75% of nadirs occurred in the first week (median day 4); [33] identified a mean onset around day 3.9 but occasional late events, and [32] observed events between days 1 and 10. Sampling time therefore substantially affects the measured frequency of hypophosphatemia.

Severity of malnutrition was one of the most consistent predictors of phosphorus vulnerability. In study [32], patients who developed refeeding hypophosphatemia had a significantly lower admission BMI than those who did not, 11.00 ± 2.34 kg/m^2^ versus 13.17 ± 2.24 kg/m^2^. Study [79] similarly found that adolescents who developed hypophosphatemia had a lower percentage of ideal body weight at admission than normophosphatemic patients, 68.4% versus 75.4%. Study [40], which included extremely underweight adult women with a mean BMI of 11.5 kg/m^2^, found that 6.8% were already hypophosphatemic at admission, and one patient had a critically low phosphate value below 0.5 mmol/L. In male patients with severe anorexia nervosa, study [44] also found admission hypophosphatemia in 29% of the cohort, despite relatively preserved calcium and magnesium.

Importantly, some adolescent studies showed that hypophosphatemia was common even when admission weight or recent weight loss did not fully explain risk. Study [75], which included adolescents with anorexia nervosa and atypical anorexia nervosa, reported hypophosphatemia in 41% of participants during admission, but found little evidence that it was strongly predicted by total weight loss, recent weight loss, or admission weight.

In monitored refeeding cohorts, higher-calorie protocols were not consistently associated with more severe phosphate complications [38,76,79]. Mild hypophosphatemia was observed, but severe clinical refeeding syndrome was not reported in these comparisons. Interpretation requires attention to protocol-specific monitoring and supplementation; these studies do not establish that monitoring can be omitted.

Not all studies showed phosphorus depletion. Some reported normal or even higher phosphorus concentrations in anorexia nervosa compared with controls. Study [71] found that acutely ill patients with anorexia nervosa had higher inorganic phosphate concentrations than both healthy controls and remitted patients. Study [60] similarly reported that serum phosphorus was significantly higher in women with anorexia nervosa than in controls, although still within the normal range; the authors suggested that this may relate to increased growth hormone levels associated with malnutrition. Study [55] also reported normal serum phosphorus in hospitalized adolescents with eating disorders, with no clear association between vitamin D deficiency and hypophosphatemia.

Individual cases demonstrated severe or prolonged phosphate decline after nutrition was restarted, including a nadir of 0.4 mg/dL [74], a recurrent disturbance after normal admission phosphate [80], and a fall to 1.9 mg/dL by day 20 [85]. These sentinel reports illustrate possible severity but cannot estimate its population frequency.

### 3.2. Iron

Interpretive summary (hematological cohorts): AN-specific clinical cohorts [45,46] demonstrate that anemia, serum iron, ferritin, and transferrin do not move in parallel. The 318-woman retrospective outpatient sample [45] is more informative for a typical hematological pattern than isolated hyperferritinemia case reports, but referral setting, exclusion criteria, inflammation, and non-equivalent iron biomarkers prevent a pooled inference about iron deficiency.

Iron status was generally preserved, and overt iron deficiency was uncommon despite frequent anemia and altered iron-related markers. In severely malnourished male patients, mean serum iron, iron-binding capacity, and transferrin saturation were all within reference ranges, and none of the six patients with available iron studies had iron deficiency, despite the extreme degree of underweight [44]. Similarly, in 53 adults with severe anorexia nervosa, anemia was very common, affecting 83% of patients. Still, iron deficiency was uncommon: only four patients with available iron studies were iron deficient, and only three of these were also anemic [46].

The 318-woman outpatient cohort [45] found low serum iron in only 6.2%, whereas ferritin was often high and transferrin sometimes low. A one-year adolescent follow-up found largely stable iron indices [95], and two inpatient adolescent studies reported mostly normal iron or improvement during nutritional rehabilitation [65,105]. Thus, anemia and abnormal iron-related proteins cannot be equated with straightforward iron deficiency.

Iron transport markers differed from serum iron. In hospitalized women, total iron-binding capacity was reduced despite normal serum iron and increased after nutritional treatment [112]. Another eating-disorder cohort reported lower binding capacity and higher ferritin in AN without significantly lower plasma iron or transferrin saturation [159]. These markers suggest altered iron handling but do not independently establish depleted body iron stores.

Ferritin findings were particularly heterogeneous. Some studies reported normal or elevated ferritin rather than depletion. In adolescents with restrictive anorexia nervosa, Papillard-Marechal et al. [116] found normal serum iron and preserved hemoglobin in most patients, but significantly elevated ferritin and hepcidin. This is a paradoxical pattern because severe undernutrition would intuitively be expected to reduce iron stores, yet ferritin and hepcidin were increased. The authors suggested that amenorrhea may help preserve iron stores by reducing menstrual iron loss, while starvation-related metabolic regulation may alter ferritin and hepcidin independently of classical inflammation. Case reports provide even stronger examples of disturbed iron regulation: one severely malnourished woman had extreme hyperferritinemia, with ferritin reaching 9096 µg/L.

In contrast, serum iron was low, and transferrin saturation was at the lower end of normal [158]. Another case showed elevated or high-normal serum iron, markedly elevated ferritin, and extremely low iron-binding capacity, again inconsistent with simple nutritional iron deficiency [160].

Only a small subset of studies reported a clearly reduced iron level. In a study of very young patients with anorexia nervosa, mean serum iron was markedly below the reference range, accompanied by reduced hematocrit and slightly reduced red blood cell count, suggesting compromised erythropoietic status [114]. In a single adolescent case, serum iron was slightly below the normal range, although zinc deficiency was the primary micronutrient abnormality, and iron indices were not explored in depth [104]. These findings show that iron deficiency or reduced iron availability can occur in anorexia nervosa, especially in selected patients. Still, it is not the most consistent biochemical abnormality across the literature. The overall pattern is therefore mixed: serum iron may be normal, low-normal, mildly reduced, or occasionally markedly reduced, whereas ferritin and iron-binding measures may be disproportionately altered.

### 3.3. Selenium

Interpretive summary (biochemical baseline status): In the 153-patient pre-treatment cohort [31], selenium was assessed in plasma and low results occurred in a substantial subgroup; the 374-inpatient cohort [48] also reported selenium deficiency in severe AN. Disease-severity associations [50] are observational and do not prove that selenium depletion causes psychiatric manifestations or that replacement alters clinical outcomes.

Selenium deficiency was a recurrent finding, especially in clinically severe or nutritionally compromised cohorts, although prevalence varied across studies. Still, individual-level analysis revealed that selenium deficiency was the most frequent trace-element abnormality, affecting 40.6% of patients [31]. Importantly, this deficiency was almost equally distributed between anorexia nervosa subtypes, occurring in 40.0% of patients with restrictive anorexia nervosa and 41.5% of those with binge–purging anorexia nervosa [31].

A similarly high prevalence was reported in the study by Strumila et al., which was specifically designed to evaluate selenium status in anorexia nervosa. In that cohort of 153 patients, selenium deficiency was defined as plasma selenium levels below 80 µg/L, a threshold commonly used and approximating the concentration required for optimal selenoprotein activity [50]. Using this criterion, 82 patients (53.6%) were selenium-deficient [50]. This makes selenium deficiency one of the most prominent micronutrient abnormalities reported in anorexia nervosa. Notably, low selenium was not simply explained by lower current BMI or lower lifetime minimum BMI, because these measures did not differ significantly between patients with low and normal selenium concentrations [50]. Instead, selenium deficiency appeared to identify a subgroup with more severe eating-disorder psychopathology. Patients with low selenium had higher EDE-Q and Body Shape Questionnaire scores, and in multivariate analyses, the EDE-Q total score remained significantly associated with low selenium concentration [50].

Studies of more severely malnourished hospitalized patients also support the clinical relevance of selenium deficiency, although the observed prevalence was somewhat lower than in the outpatient or mixed clinical cohorts. In a large cohort of 374 adults with extremely severe anorexia nervosa, selenium deficiency was present in 20.5% of patients [48]. This study is informative because it included patients with profound malnutrition and compared the restrictive and binge–purging subtypes. Unlike the earlier cohort in which selenium deficiency was nearly identical between subtypes, this study found that selenium concentrations were significantly lower in patients with binge–purging anorexia nervosa than in those with restrictive anorexia nervosa [48]. This may suggest that, under conditions of extreme malnutrition, binge–purging behaviors or associated dietary and metabolic instability can further compromise selenium status. Another cohort of 354 adults with extremely severe anorexia nervosa reported a very similar prevalence: selenium deficiency was found in 41 of 192 tested patients, or 21.3% [49]. These patients had an exceptionally low mean BMI of 12.2 kg/m^2^. They were frequently treated with enteral nutrition, suggesting that selenium depletion may be part of a broader micronutrient-deficiency profile in advanced anorexia nervosa, alongside deficiencies in vitamin D, zinc, copper, folate, and vitamin B12 [49].

Low selenium was associated with greater psychopathology and a history of suicide attempts in an AN cohort [50]. A longitudinal cohort of 658 patients with eating disorders (56.7% with AN) also linked lower baseline selenium with later suicide-attempt risk after adjustment for clinical covariates [70]. Because this latter sample was diagnostically mixed and both analyses were observational, the findings should be understood as associations rather than evidence of causality.

Rare cases linked marked selenium depletion to macrocytic anemia that had not responded to B12/folate replacement [85] and to myopathy with low glutathione peroxidase activity during parenteral nutrition [156]. Improvement after selenium replacement was described, but these uncontrolled cases cannot establish treatment efficacy or frequency of complications in AN.

### 3.4. Magnesium

Interpretive summary (baseline versus refeeding): Plasma magnesium was usually within range in the 153-patient pre-treatment cohort [31], but serial inpatient measurements identified new hypomagnesemia during nutritional rehabilitation [38]. A normal baseline serum/plasma magnesium value neither reflects intracellular stores directly nor predicts subsequent refeeding stability; purging and laxative exposure also require separate interpretation.

Baseline magnesium was commonly within reference ranges, including a 153-woman pre-treatment cohort with only 3.0% classified as deficient [31]. Severe male inpatients [44], other markedly underweight adults [39,82], and medically hospitalized adolescents [78] likewise often had normal admission measurements. These findings describe circulating magnesium at sampling, not necessarily intracellular stores.

However, studies that monitored patients during refeeding showed that magnesium disturbances may emerge dynamically and may therefore be underestimated by admission values alone. This was particularly clear in the study of hospitalized adolescents with restrictive eating disorders, where 15 of 171 participants, or 9%, developed hypomagnesemia during admission, defined as magnesium below 1.4 mEq/L or 0.70 mmol/L [75]. In the randomized refeeding trial by Golden et al., hypomagnesemia occurred in 47 of 310 adolescents and young adults (15.2%) during hospitalization. However, its frequency did not differ significantly between the lower- and higher-calorie refeeding groups [38]. The strongest evidence for clinically relevant magnesium instability came from an observational refeeding cohort of 50 adults with anorexia nervosa, in which serum magnesium was measured daily during the first 5 days and then repeatedly throughout the admission. In that study, 60% of patients developed low serum magnesium at some point during hospitalization; only 16% were already hypomagnesemic on admission, whereas 44% developed hypomagnesemia later during treatment [151].

The relationship between magnesium and refeeding complications was less consistent than that observed for phosphate. In the cohort of 123 women with severe anorexia nervosa admitted for medical stabilization, admission magnesium was within the reference range overall, with a median of 1.8 mEq/L. Magnesium differed statistically in the unadjusted comparison between patients who did and did not develop refeeding hypophosphatemia, but it did not remain an independent predictor in the final multivariable model [34]. This suggests that magnesium may contribute to the broader electrolyte instability of refeeding. Still, it was not as robust a predictor of refeeding hypophosphatemia as other markers, such as potassium, BMI, prealbumin, or hemoglobin, in that dataset. Similarly, in adolescents with anorexia nervosa or atypical anorexia nervosa, hypomagnesemia and magnesium nadir were not strongly associated with admission weight, total weight loss, or recent weight loss [75].

Subgroup findings did not establish a consistent sex- or AN-subtype pattern. In [36], higher measured magnesium in the binge–purge subtype was potentially confounded by magnesium-containing laxatives. A small atypical-AN comparison also found little hypomagnesemia and no clear group differences [86]. Purging, renal handling, laxatives, and replacement therapy remain important interpretive confounders.

Clinical cases ranged from mild hypomagnesemia in complicated illness [108] to preserved magnesium despite severe bone disease [121]. A case involving laxative and diuretic misuse reported low magnesium with refractory hypokalemia and hypocalcemia that improved after magnesium administration [122]. These observations identify possible clinical consequences without defining prevalence.

### 3.5. Potassium

Interpretive summary (subtype and treatment context): Admission serum potassium can be normal even in profound restriction [35,73]. AN-specific admissions data show substantially lower potassium in binge–purge than restricting presentations [36,37], while hospitalization and refeeding introduce additional changes [38]. Subtype, gastrointestinal/renal losses, medical instability, and the sampling time must be considered before attributing low potassium to chronic dietary restriction.

Admission potassium was often normal in restricting presentations, including severe AN cohorts [35,39,73], medically hospitalized young people [78], and an individual severely malnourished patient [80]. Consequently, profound underweight alone did not uniformly correspond to baseline hypokalemia; recent losses and clinical phenotype remain relevant.

AN subtype showed a clearer signal than overall underweight: mean potassium was lower in binge–purge than restricting AN in [36] (3.2 versus 4.0 mmol/L) and [37] (2.8 versus 3.8 mmol/L). The same direction was observed in [88]; in a large mixed severe eating-disorder inpatient cohort, hypokalemia affected 39%, with binge–purge patients overrepresented [82]. Vomiting, laxatives, diuretics, dehydration, and related renal losses may contribute to these differences.

Potassium abnormalities were also relevant in refeeding studies, although they were generally less dominant than phosphate disturbances. In adolescents with anorexia nervosa or atypical anorexia nervosa, study [75] found that only 5 of 171 patients, or 3%, developed hypokalemia during admission, and potassium nadir was not clearly associated with admission weight, total weight loss, or recent weight loss. In contrast, Golden et al. [38], in a larger cohort of hospitalized adolescents and young adults, reported that 62 of 310 patients (20.0%) developed hypokalemia, defined as potassium ≤ 3.5 mEq/L. Notably, electrolyte abnormalities did not differ significantly between lower-calorie and higher-calorie refeeding groups, and no patient developed full clinical refeeding syndrome. Study [34] provides a more mechanistic refeeding-risk interpretation: in 123 women with severe anorexia nervosa, the median admission potassium was 3.6 mmol/L overall, but patients who later developed refeeding hypophosphatemia had a lower admission potassium of 3.2 mmol/L than those who did not (3.7 mmol/L). In the adjusted model, higher potassium remained an independent protective factor against hypophosphatemia.

Selected severe cases documented admission potassium of 2.9 mmol/L [85], 2.8 mEq/L [93], and 2.7 mEq/L [121], often alongside other nutritional or renal disturbances. These cases illustrate clinical extremes but are not representative prevalence evidence.

Serum potassium may not reflect whole-body potassium availability. Although fasting serum values were near normal in some AN patients [96], another study found normal serum potassium but substantially lower 24 h urinary potassium excretion than in thin and normal-weight controls [111], consistent with adaptive conservation. An adolescent outpatient study similarly reported no overt hypokalemia [103]. Urinary and serum measures therefore cannot be interpreted interchangeably.

### 3.6. Sodium

Interpretive summary (fluid balance and acute illness): Sodium is predominantly described using single serum measurements at admission; severe hyponatremia is concentrated in selected medically unstable patients [39,40]. The small repeated-measures study of 14 severe AN patients [41] illustrates sodium and plasma-volume depletion despite potentially misleading serum concentration. Hydration, water loading, purging and intravenous fluids limit comparison between cohorts.

Sodium was usually preserved, but clinically important hyponatremia occurred in medically unstable, purging, dehydrated, or water-loading subgroups. In the small refeeding study by Gentile [73], patients were extremely undernourished, with a mean BMI of only 11.2 kg/m^2^, yet baseline sodium was preserved, with a mean value of 140.4 ± 2.8 mmol/L and a range of 136–145 mmol/L. This suggests that even in life-threatening malnutrition, sodium may appear normal on admission. However, the authors considered sodium and fluid balance clinically important because refeeding can promote sodium retention, edema, and cardiac complications. Similarly, in the severe anorexia nervosa cohort described in the study [39], sodium remained largely stable before treatment, with a median lowest value of 137 mmol/L. Nevertheless, one patient had severe hyponatremia below 120 mmol/L before treatment, showing that although sodium abnormalities were uncommon in the group as a whole, they could be profound in individual cases.

A similar pattern was observed in larger cohorts of extremely underweight patients undergoing medical stabilization or refeeding. In study [40], which included 103 adult female patients with very severe anorexia nervosa and a mean initial BMI of 11.5 ± 0.9 kg/m^2^, the mean sodium at admission was 138.7 ± 4.7 mmol/L, again within the normal range on average. However, the reported range was 112.0 to 152.0 mmol/L, and seven patients (6.8% of the sample) had pathological sodium values; one patient had critical hyponatremia below 125 mmol/L at admission. This shows that serum sodium may be normal at the cohort level while still identifying a subset of patients with potentially life-threatening hypo- or hypernatremia. In study [34], median admission sodium in 123 women with severe anorexia nervosa was 136 mmol/L, with an interquartile range of 134–140 mmol/L and a full range of 117–150 mmol/L. Patients who subsequently developed refeeding hypophosphatemia had lower admission sodium than those who did not, with median values of 134 versus 137 mmol/L. This association was statistically significant in bivariate analysis, but sodium did not remain an independent predictor after adjustment for other clinical and biochemical variables.

Among 545 severely ill inpatients with restrictive eating disorders, 25% had admission hyponatremia despite a near-normal mean sodium (136.9 mmol/L); reported abnormalities often resolved during early stabilization [82]. In 232 adults with AN, hyponatremia was more frequent in binge–purge than restricting presentations (57.7% versus 38.5%) [88]. Mixed diagnostic groups, fluid balance and purging behavior limit direct comparisons across cohorts.

Several smaller physiological and case-based studies further clarify the heterogeneity of sodium findings. In study [41], 14 severely malnourished female patients with anorexia nervosa had a mean admission sodium of 137.93 ± 6.83 mmol/L. Hyponatremia, defined as sodium below 135 mmol/L, was present in three patients and was associated with hypo-osmolality and hypovolemia, indicating hypotonic dehydration or hypovolemic hyponatremia rather than a simple dietary sodium deficiency. In the renal and osmoregulatory study [145], patients with anorexia nervosa had significantly lower plasma sodium than healthy controls, with values of 136 ± 0.9 versus 139 ± 0.6 mEq/L, as well as lower chloride and plasma osmolality. However, these values remained within the normal laboratory range, and the authors emphasized that there were no major electrolyte abnormalities.

Sodium was preserved in several adolescent and outpatient investigations [65,111] and in selected severe clinical cases despite concurrent disturbances of other electrolytes [85,121,123,148,149]. In [111], normal serum sodium and urinary sodium contrasted with reduced urinary potassium, underscoring distinct physiological regulation. These cases do not negate hyponatremia observed in unstable inpatient populations.

In many patients, especially adolescents, restricting-type cases, or medically monitored cohorts, serum sodium remains within the normal range despite severe undernutrition. However, hyponatremia appears repeatedly in subsets of patients with severe illness, purging behaviors, water-balance disturbance, hypovolemia, low solute intake, or acute medical instability. Large cohorts indicate that hyponatremia may affect a meaningful minority of hospitalized patients, ranging from approximately 7% in a community-dwelling anorexia nervosa cohort [127] to 25% in a large inpatient stabilization cohort [82], with even higher prevalence reported in severe binge–purge anorexia nervosa [88].

### 3.7. Calcium

Interpretive summary (regulated serum marker versus skeletal status): The AN-specific pre-treatment cohort of 153 patients [31] generally showed preserved serum/plasma calcium, whereas bone-focused studies [42,43] assessed different compartments or outcomes. Calcium concentration is regulated independently of bone mineral density; normal serum calcium cannot be used to infer adequate bone reserves or to evaluate supplementation efficacy.

Most AN cohorts reported normal circulating calcium. Hypocalcemia was uncommon in 153 women studied before treatment (1.3%) [31], and serum calcium was preserved in comparisons with healthy controls [71] and in severely malnourished male and adult clinical cohorts [39,44]. In 545 severely ill hospitalized patients, 95% had calcium within laboratory reference limits [82]. Normal values should not be treated as an indicator of skeletal sufficiency.

Normal calcium was also observed in studies of vitamin D and bone metabolism. Patients with AN and controls had similar calcium in [60] and [56], while a diagnostically mixed adolescent eating-disorder cohort reported no hypocalcemia despite suboptimal vitamin D status in a subgroup [55]. Thus, vitamin D abnormalities and bone-related disease may occur without changes in tightly regulated serum calcium.

However, the absence of low serum calcium should not be interpreted as evidence that calcium nutrition is adequate or that calcium-related skeletal risk is absent. Several studies show that serum calcium and calcium balance are not equivalent. In one study of 113 women with anorexia nervosa, mean serum calcium was normal at 93.64 ± 4.65 mg/L, ionized calcium averaged 50.36 ± 1.91 mg/L, serum phosphorus was also normal, and parathyroid hormone averaged 36.2 ± 17.6 pg/mL; nevertheless, dietary calcium intake was frequently insufficient, with a mean intake of 907.7 ± 317.36 mg/day and 78% of patients consuming less than 1200 mg/day [107]. This distinction is important because circulating calcium is maintained by renal, intestinal, parathyroid, vitamin D-dependent, and skeletal buffering mechanisms. Thus, normal serum calcium levels may coexist with inadequate calcium intake, low bone mineral density, amenorrhea, hypoestrogenism, altered IGF-I levels, and increased fracture risk [107]. A similar interpretation appears in the study of 29 women with anorexia nervosa, including 18 with bulimic symptoms, where serum calcium was normal in all patients, with a mean value of 4.5 ± 0.2 mEq/L within the reference range of 3.5–5.0 mEq/L. However, the authors emphasized that calcium intake was not reliably measured and that low calcium intake could still contribute to reduced bone mineral density [43]. In other words, serum calcium was normal, but bone mineral status was not necessarily protected [43].

Some studies provided more direct evidence that calcium handling may be altered even when serum calcium is normal. In the staged study of 73 postpubertal female patients with anorexia nervosa, calcium metabolism differed across active illness, weight recovery with persistent amenorrhea, and full recovery [120]. During active anorexia nervosa, patients had abnormally elevated urinary calcium excretion, with a mean calciuria of 6.6 mg/kg/day, above the normal value of less than 4.0 mg/kg/day [120]. The urinary calcium-to-creatinine ratio was also highest during the active undernourished phase, suggesting unfavorable calcium balance and increased renal calcium loss [120]. After weight recovery, calciuria improved markedly, falling to 3.1 mg/kg/day in both partially and fully recovered stages, while the calcium-to-creatinine ratio also decreased [120]. This suggests that calcium loss may be state-dependent and linked to active undernutrition, hypoestrogenism, low IGF-I, and broader metabolic adaptation rather than representing a fixed abnormality [120]. This shows that the calcium problem in anorexia nervosa may be expressed not as hypocalcemia, but as increased urinary calcium loss and impaired skeletal retention during the active disease phase.

During nutritional rehabilitation, serum calcium remained largely stable despite substantial calcium intake in an adolescent inpatient study [146]. A longer follow-up study also showed mostly normal calcium across treatment and subsequent years, even as bone-density abnormalities persisted [132]. These observations separate circulating calcium from skeletal recovery and do not isolate supplementation effects from overall rehabilitation.

Hypocalcemia was occasionally documented in severe, medically complicated AN: a prolonged low-calcium course during hospitalization [85] and hypocalcemia with hypoproteinemia, edema and zinc depletion [93]. By contrast, another medically unstable case had normal calcium despite marked nutritional abnormalities [108]. Case reports illustrate heterogeneity but cannot establish a typical AN calcium pattern.

### 3.8. Zinc

Interpretive summary (severity and diagnostic specificity): Severe adult AN inpatient cohorts reported a substantial burden of low circulating zinc [48,49], whereas the mixed AN/ARFID cohort [47] also found subtype differences and requires subgroup-aware reading. Studies using plasma, serum, urinary or other zinc indicators cannot be counted as interchangeable, and the absence of universal depletion should be maintained.

Zinc disturbance was common but heterogeneous, with the strongest deficiency signal in severe and hospitalized cohorts.

In contrast, patients with restricting-type anorexia nervosa had higher values, around 65.7 µg/dL, and those with ARFID had the highest mean values, around 69.0 µg/dL [47].

The high frequency of zinc deficiency was especially evident in large cohorts of extremely malnourished inpatients. In one large study of 374 patients with severe anorexia nervosa, zinc deficiency was the most common micronutrient abnormality, affecting 64.3% of patients. Importantly, zinc deficiency was frequent in both restricting and binge–purging anorexia nervosa, with no significant difference between subtypes. This finding indicates that zinc depletion may be a broad consequence of severe dietary restriction, chronic undernutrition, and advanced illness rather than a subtype-specific abnormality [48]. A similar pattern was observed in another large cohort of 354 adults with extremely severe anorexia nervosa, where the mean admission BMI was only 12.2 kg/m^2^. In this group, zinc deficiency was found in 56.8% of tested patients, making it one of the most frequent micronutrient deficiencies after vitamin D deficiency [49].

However, not all studies found high rates of zinc deficiency. In a retrospective study of 153 adult female patients with anorexia nervosa evaluated before nutritional treatment, mean plasma zinc values were largely within the normal range, and zinc deficiency was rare, occurring in only 2.1% of patients [31]. This contrasts sharply with the inpatient cohorts described above and suggests that the prevalence of zinc deficiency depends strongly on illness severity, population characteristics, the biological compartment measured, reference cutoffs, previous supplementation, and sampling timing. Similarly, in a study comparing acutely ill, remitted, and healthy female participants, acutely ill patients with anorexia nervosa did not have significantly lower serum zinc than controls. Instead, the most striking finding was that remitted patients had significantly higher serum zinc concentrations than both acutely ill patients and healthy controls, with mean values of 81.75 µg/dL in remitted patients, 62.23 µg/dL in acutely ill patients, and 59.95 µg/dL in controls [71]. Therefore, although low zinc is common in many anorexia nervosa cohorts, zinc findings are not universally reduced across all clinical samples.

Pediatric and adolescent evidence also identified low zinc in subsets of restrictive eating-disorder patients. In childhood-onset AN, zinc correlated with nutritional status [72]; zinc deficiency affected about one-quarter of a large hospitalized youth sample, including both sexes [81]. In an AN/ARFID comparison, 53.3% of AN patients and 46.2% of ARFID patients had low zinc, without a clear between-group difference [84]. These mixed populations warrant caution in AN-specific prevalence inferences.

Evidence from atypical anorexia nervosa further supports this point. In hospitalized male adolescents and young adults with atypical anorexia nervosa, mean plasma zinc was 64.7 µg/dL, and 31.6% had low zinc according to the cutoff of <55 µg/dL [86]. These patients were not underweight by conventional BMI criteria, with a mean percent median BMI of approximately 102%, but they had substantial weight suppression. This suggests that zinc deficiency may occur not only in classical underweight anorexia nervosa, but also in atypical anorexia nervosa, where recent or marked weight loss may produce biochemical nutritional risk despite apparently normal body weight.

Older biochemical studies provide a more complex picture, particularly because they assessed different zinc compartments. In adolescent patients with anorexia nervosa, one study found significantly reduced 24 h urinary zinc excretion, with mean values of 257.1 µg/24 h compared with 749.9 µg/24 h in controls, whereas serum zinc itself was not significantly lower, 73.9 versus 80.1 µg/dL [91]. This pattern suggests that serum zinc may remain apparently normal even when urinary zinc conservation indicates depleted or marginal zinc status. Another study of 24 patients with anorexia nervosa and 62 bulimic patients defined zinc deficiency using either low serum zinc or low urinary zinc excretion. On this combined basis, zinc deficiency was present in 54% of patients with anorexia nervosa and 40% of bulimic patients [92]. This finding is particularly relevant because it shows that zinc disturbance is not exclusive to severely underweight patients and may also occur in other eating-disorder phenotypes.

Other studies emphasized that plasma zinc alone may be an unreliable marker of the true zinc state. In a short report of 14 women with severe anorexia nervosa, the mean plasma zinc was within the normal laboratory range, and it did not differ clearly from controls, but individual abnormalities were still observed. Three patients had low plasma zinc, and three had low neutrophil zinc, a marker of tissue zinc state. Importantly, one patient had low neutrophil zinc despite normal plasma zinc and albumin, whereas another had low plasma zinc but apparently adequate neutrophil zinc. The authors therefore argued that plasma zinc alone may not accurately reflect tissue zinc status in anorexia nervosa [98]. Similarly, a zinc absorption study found that fasting serum zinc concentrations were similar between patients with anorexia nervosa and controls, but the rise in serum zinc after a zinc-supplemented meal was significantly blunted in patients with anorexia nervosa. This suggests that zinc metabolism, absorption, or postprandial handling may be abnormal even when fasting zinc appears normal [109].

Several studies also indicate that zinc status may change during nutritional rehabilitation. In adolescent girls with restricting-type anorexia nervosa followed for one year, serum zinc was not clearly depleted at admission, but zinc became significantly lower than that of controls at six months. Decreases in zinc were negatively correlated with increases in BMI, body weight, and percentage of ideal body weight, suggesting that zinc may be consumed or redistributed during anabolic recovery [95]. In another adolescent inpatient study, zinc deficiency was already very common at admission and persisted despite short-term weight gain: mean zinc was 64.5 µg/dL at admission and 65.7 µg/dL at discharge, both below the reference range of 80–120 µg/dL. Deficiency affected 84.1% of patients at admission and remained present in 73.4% at discharge [65]. These findings suggest that refeeding alone may not rapidly normalize zinc status and that zinc requirements may increase during tissue repair, weight restoration, and metabolic recovery.

Severe zinc depletion was reported in isolated AN cases with dermatitis, alopecia, edema, impaired taste or other complications [93,104,108]. These reports demonstrate possible presentations of profound deficiency but should not be used to estimate its frequency.

### 3.9. Chloride

Interpretive summary (purging and medical complications): Chloride abnormalities are most interpretable as disturbances of gastric losses, volume regulation, and acid–base physiology rather than as evidence of a distinct chronic micronutrient deficiency [41,51]. The evidence is dominated by small clinical series and individual medically complicated presentations; isolated values are strongly dependent on current purging and treatment.

Chloride was usually normal or only mildly reduced; clearer hypochloremia occurred mainly with purging, vomiting, laxative use, or broader fluid–electrolyte disturbance. For example, in one severely undernourished 19-year-old woman with anorexia nervosa, starvation, and laxative abuse, admission chloride was 100 mEq/L, while sodium, potassium, bicarbonate, calcium, magnesium, and phosphate were also initially within or close to reference ranges; the main subsequent abnormality was refeeding-related phosphate depletion rather than chloride disturbance [51]. Similarly, in a 16-year-old girl with extreme anorexia nervosa, chronic restriction, laxative abuse, and later severe hypophosphatemia after binge eating, admission chloride was also 100 mmol/L and was described together with other initially normal biochemical values, again showing that a single baseline chloride measurement may not fully reflect the severity of total-body depletion or later metabolic risk [123]. In another case report of a 35-year-old woman with long-standing, life-threatening anorexia nervosa, chloride remained broadly near-normal during the reported course, whereas phosphorus dysregulation and refeeding-associated hypophosphatemia were the central biochemical abnormalities [148].

However, other studies suggest that chloride can be significantly lower in anorexia nervosa or mixed eating-disorder samples, even when values remain within or near the normal laboratory range. In a study focused on renal function, water balance, osmoregulation, and antidiuretic hormone response, female patients with anorexia nervosa had significantly lower plasma chloride levels than healthy controls (101 ± 1.3 mEq/L versus 104 ± 0.7 mEq/L). This difference paralleled lower sodium and lower plasma osmolality in the anorexia nervosa group, suggesting a relative hypo-osmolar state rather than isolated chloride depletion. Importantly, the authors emphasized that major electrolyte abnormalities were not present, so the chloride reduction should be interpreted as a statistically detectable but clinically modest shift in the context of altered water and sodium–chloride regulation [145]. A broader biochemical study by Mira et al. also found significantly lower chloride in women with eating disorders than in controls. In that sample, mean chloride was 102.6 mmol/L in patients compared with 106.1 mmol/L in controls, and individual patient values reached as low as 85 mmol/L [147]. This range indicates that although the group mean suggested only a moderate reduction, a subset of patients had clear hypochloremia. Because the same study also reported lower potassium and phosphate, and because chloride depletion in eating disorders is often linked to purging-related loss of gastric hydrochloric acid, these findings support the view that low chloride is most clinically relevant in patients with purging physiology, vomiting, dehydration, or acid–base disturbance rather than in all patients with restrictive anorexia nervosa.

One longitudinal case report provides useful detail on how chloride may change during nutritional stabilization. In an 18-year-old female with extremely severe anorexia nervosa, chloride was low at admission, 98 mmol/L, relative to the stated normal range of 101–108 mmol/L. During hospitalization, it increased to 102 mmol/L by day 20, then reached 109 mmol/L on day 40, 108 mmol/L on day 60 and day 66, 106 mmol/L on day 73, 104 mmol/L on day 83, and 101 mmol/L on day 90 [85].

### 3.10. Copper

Interpretive summary (AN-specific admission studies): Copper deficiency was documented in severely malnourished AN inpatient cohorts [48,49], while the 153-patient assessment [31] showed a less uniform pattern. Serum/plasma copper and hair copper reflect different biological compartments; treatment setting and acute-phase responses restrict generalization from one matrix to another.

Copper status was severity-dependent, with more frequent hypocupremia in severely malnourished inpatients and more preserved values in milder or mixed cohorts. Importantly, this study also reported a subtype-related difference: copper levels were significantly lower in patients with restricting anorexia nervosa than in those with binge-eating/purging anorexia nervosa, suggesting that copper depletion may be particularly associated with prolonged restrictive undernutrition rather than purging behavior alone [48]. A similar pattern was observed in another cohort of 354 adults with extremely severe anorexia nervosa, where mean BMI at admission was only 12.2 ± 1.6 kg/m^2^. In this highly compromised population, copper deficiency was found in 44 of 146 tested patients, corresponding to 30.1%. This places copper among the more frequent micronutrient deficiencies in severe anorexia nervosa, after vitamin D and zinc, but before selenium, folate, and vitamin B12 [49].

Earlier biochemical studies also support the view that copper can be selectively reduced in anorexia nervosa. Casper et al. [112] reported significantly lower plasma copper concentrations in hospitalized female patients with anorexia nervosa than in controls, with mean values of approximately 84 µg/100 mL in patients compared with about 106 µg/100 mL in healthy women. This reduction occurred alongside reduced zinc, but ceruloplasmin was not significantly depressed, suggesting that low plasma copper was not fully explained by impaired ceruloplasmin synthesis. The authors interpreted the parallel reductions in zinc and copper as likely consequences of dietary restriction, with the copper deficit generally moderate rather than severe. Hemoglobin synthesis did not appear broadly impaired, although two patients with leukopenia had low copper concentrations, suggesting that hypocupremia may contribute to hematological vulnerability in selected individuals [112]. Similarly, in the related study summarized in [113], plasma copper was significantly lower in patients with anorexia nervosa than in controls, with mean concentrations of 85.5 ± 17.9 µg/dL versus 123.2 ± 3.9 µg/dL, respectively. Interestingly, hair copper was increased in a subset of patients, suggesting that anorexia nervosa may involve altered copper distribution between plasma and tissues rather than simple whole-body copper depletion. This distinction is important because serum or plasma copper reflects circulating transport and protein-binding dynamics, not necessarily total-body copper stores [113].

The pediatric and adolescent literature also suggests that copper deficiency may occur in a clinically meaningful subgroup, although findings are not uniform. In one study of 13 very young patients with anorexia nervosa, the mean serum copper was 82.8 ± 26 µg/dL, but 6 of 13 patients, or 46%, had copper concentrations below the normal reference range of 70–150 µg/dL [114]. This indicates that nearly half of this small sample showed biochemical hypocupremia, even though zinc remained normal on average. The contrast between copper and zinc suggests that trace-element abnormalities in anorexia nervosa do not affect all elements equally. In another adolescent study, however, copper concentrations did not differ significantly between patients with anorexia nervosa and controls. Mean serum copper was 79.7 ± 17.9 µg/dL in adolescents with anorexia nervosa and 87.9 ± 11.9 µg/dL in controls, indicating a numerically lower but statistically non-significant difference [91].

Other studies reported preserved copper status, especially in less severely malnourished or diagnostically mixed populations. In a retrospective study of 153 adult female patients with anorexia nervosa assessed before nutritional treatment, copper deficiency was not observed, even though 43.7% of patients had at least one trace-element deficiency [31]. In this cohort, selenium deficiency was the dominant trace-element abnormality, whereas zinc deficiency was rare and copper deficiency was absent. This finding contrasts sharply with the large severe-inpatient cohorts, suggesting that copper deficiency may be strongly influenced by the degree of malnutrition and clinical severity. A single case study of a 16-year-old girl with anorexia nervosa also reported normal serum copper, with a value of 120 µg/dL within the reference range of 89–147 µg/dL, despite severe weight loss, hypogeusia, low alkaline phosphatase activity, borderline-low zinc, and slightly low iron [104]. Similarly, Mira, Stewart, and Abraham [53], in an outpatient sample of women with eating disorders that included anorexia nervosa, bulimia nervosa, and atypical eating disorders, found no significant differences in plasma copper between controls, non-supplemented eating-disorder patients, and supplemented eating-disorder patients. Median plasma copper values were very similar across groups: 15.5 µmol/L in controls, 15.4 µmol/L in non-supplemented patients, and 14.8 µmol/L in supplemented patients. The authors suggested that copper deficiency may be unusual outside the context of prolonged and severe nutritional deficiency, which likely explains why it was not prominent in this outpatient, diagnostically mixed cohort [53].

Overall, the copper findings suggest that hypocupremia is not a consistent biochemical feature of all patients with anorexia nervosa, but it becomes increasingly evident in severe, chronic, and hospitalized cases. Across the available studies, copper deficiency ranges from absent in some adult female cohorts [31] and outpatient mixed eating-disorder samples [53], to approximately 30–37% in large cohorts of severely malnourished inpatients [48,49], and up to 46% in a small pediatric sample [114]. Earlier controlled studies further show that mean plasma or serum copper may be significantly reduced in anorexia nervosa even when other nutritional indices, vitamins, albumin, or ceruloplasmin are relatively preserved [112,113,115]. Therefore, copper status in anorexia nervosa should be interpreted as severity-dependent and compartment-sensitive. Normal copper values in milder or outpatient samples do not exclude clinically relevant hypocupremia in more severely malnourished patients. Conversely, reduced plasma copper does not always prove global copper depletion, because altered ceruloplasmin binding, hepatic protein synthesis, redistribution, inflammation, supplementation history, and refeeding state may all influence circulating copper concentrations. Clinically, these findings support routine monitoring of copper, particularly in patients with very low BMI, long illness duration, restrictive eating patterns, recurrent hospitalization, hematological abnormalities, or signs of severe micronutrient depletion.

### 3.11. Manganese

Interpretive summary (sparse mixed-diagnosis evidence): Available manganese measurements chiefly derive from one heterogeneous eating-disorder investigation [52], with limited extractable AN-specific information. Results should be treated as preliminary evidence about circulating manganese, not as an established AN-specific prevalence pattern or a basis for routine manganese supplementation.

In the study [52], the most striking abnormality was manganese deficiency. The total-group mean manganese level was 8.3 nmol/L, far below the reference interval of 10.0–45.0 nmol/L and the population midpoint of 27.5 nmol/L. The range was 4.0 to 21.0 nmol/L, and the difference from the general population was extremely significant. In reference-interval analysis, 76 of 106 patients had manganese levels outside the reference interval, making manganese the parameter with the largest number of most abnormal results. The low manganese pattern was present in all subgroups: AN 7.79 nmol/L, BN 7.19 nmol/L, EDNOS 8.17 nmol/L, and AN/BN 11.4 nmol/L, with all subgroup differences statistically significant. The authors emphasize that manganese deficiency is rarely reported in humans, which makes the finding particularly notable.

### 3.12. Vitamin A

Interpretive summary (different analytes): In the 153-patient baseline sample [31], a subset had low circulating retinol; several other studies described relatively preserved retinol despite abnormal carotenoids or related markers [53,54]. Retinol, carotenoids, and retinoid fractions must not be combined into one deficiency or excess direction, and altered hepatic handling may contribute.

Vitamin A findings were heterogeneous: frank deficiency occurred in some patients, whereas preserved retinol and elevated carotenoid or retinoid markers were also common.

The clearest evidence for vitamin A deficiency came from the large retrospective clinical study [31], which assessed baseline micronutrient status in 153 female patients with anorexia nervosa before nutritional treatment. Although mean plasma vitamin A concentrations were mostly within the normal range at the group level, individual-level analysis showed that vitamin A deficiency was the most frequent vitamin deficit, affecting 32.8% of patients. Deficiency was numerically more frequent in restrictive anorexia nervosa than in the binge–purging subtype, with rates of 35.7% and 28.3%, respectively, although this subtype difference was not statistically significant. These findings are important because they show that normal group averages may conceal a substantial subgroup of patients with clinically relevant vitamin A deficiency. They also suggest that anorexia nervosa subtype alone is not a reliable predictor of vitamin A status, since both restrictive and binge–purging patients showed measurable risk of deficiency.

In contrast, several older biochemical studies did not find reduced circulating retinol in anorexia nervosa. Pettersson et al. [77], in a three-year follow-up study of young women previously hospitalized for severe restrictive anorexia nervosa, found that serum retinol was adequate on average, with a mean of 2.2 µmol/L and a range of 1.2–3.7 µmol/L; none of the participants were below the reference range. This preservation of serum retinol occurred despite persistent dietary inadequacy and incomplete nutritional recovery, suggesting that circulating vitamin A may remain normal even when dietary intake is vulnerable. Similarly, Langan and Farrell [101] reported normal plasma retinol in 17 hospitalized patients with anorexia nervosa. Mean retinol was 58.7 ± 19.8 µg/dL in anorexia nervosa compared with 53.7 ± 14.7 µg/dL in controls, with no significant difference. The authors further noted that retinol values were uniformly within the normal range and well above concentrations considered deficient. Rock and Vasantharajan [128] also found preserved plasma retinol in patients with eating disorders, most of whom had a history of anorexia nervosa, with a mean value of 1.90 ± 0.71 µmol/L.

Several studies instead highlighted hypercarotenemia as a characteristic vitamin A-related abnormality in anorexia nervosa. In study [96], serum carotene was 194 ± 33 µg/dL, close to the upper limit of the normal range of 70–200 µg/dL. Study [104], a case report of a severely undernourished 16-year-old girl with anorexia nervosa, found elevated serum carotene at 484 µg/dL, above the reference range of 80–400 µg/dL, together with yellowish discoloration of the palms and soles, whereas serum vitamin A itself remained normal at 865 ng/mL. Casper et al. [112] similarly reported significantly elevated plasma carotene in hospitalized female patients with anorexia nervosa, averaging 214.4 ± 129 µg/100 mL compared with approximately 135 ± 48 µg/100 mL in controls. In that study, plasma vitamin A and retinol-binding protein were normal, and zinc did not correlate significantly with carotene, vitamin A, or retinol-binding protein. Therefore, the authors did not support a simple mechanism in which zinc deficiency reduces retinol-binding protein synthesis and thereby causes impaired vitamin A transport. Instead, hypercarotenemia was interpreted as potentially reflecting high intake of carotenoid-rich foods, altered metabolism, altered conversion of carotene to vitamin A, or other starvation-related mechanisms. A related study [113] also found significantly elevated carotene in anorexia nervosa, with mean values of approximately 218 ± 200 µg/dL compared with 132 ± 39.2 µg/dL in controls, while vitamin A and retinol-binding protein were not significantly reduced.

More detailed vitamin A fractionation studies showed that anorexia nervosa may involve altered distribution of vitamin A-related compounds rather than simple retinol deficiency. In study [99], patients with anorexia nervosa or associated eating disorders were divided according to serum carotene status. Serum retinol and retinol-binding protein were normal and did not differ significantly from controls, but retinyl esters were elevated in the hypercarotenemic anorexic and bulimic groups. In anorexic patients with normal carotene, mean retinol was 99.5 µg/dL and retinyl esters were 4.21 µg/dL; in anorexic patients with elevated carotene, mean retinol was 66.8 µg/dL and retinyl esters rose markedly to 29.04 µg/dL; in controls, mean retinol was 91.6 µg/dL and retinyl esters were not detected. Carotenoid concentrations were also substantially elevated in the anorexic hypercarotenemic group, with total carotenoids of 306.3 µg/dL compared with 78.5 µg/dL in controls.

Study [100] reported an even more pronounced pattern of increased vitamin A-related compounds. In eight female patients with anorexia nervosa, total serum carotene values were markedly elevated in every patient, ranging from 520 to 1859 µg/100 mL, compared with a listed normal range of 50–250 µg/100 mL. Mean β-carotene was 98.86 ± 17.22 µg/100 mL in anorexia nervosa compared with 21.08 ± 5.63 µg/100 mL in controls, and this difference was highly significant. Retinyl esters were also elevated, with a mean of 17.92 ± 2.74 µg/100 mL compared with 10.25 ± 2.86 µg/100 mL in controls. In addition, mean serum retinol was increased in anorexia nervosa, reaching 152.74 ± 56 µg/100 mL compared with 85.92 ± 44 µg/100 mL in controls, and retinoic acid was also significantly higher, whereas retinal did not differ significantly.

Adolescent data also suggest that carotenoid abnormalities may occur without frank vitamin A deficiency. In study [65], alpha-carotene and beta-carotene were generally within reference ranges at the group level, but elevated values were present in a subset of patients. At admission, 20.6% had elevated alpha-carotene and 9.5% had elevated beta-carotene; these proportions decreased to 14.1% and 1.6%, respectively, at discharge. The authors stated that, apart from folic acid, no absolute vitamin deficiency was found.

Finally, Philipp et al. [63], who studied women with anorexia nervosa and bulimia nervosa, found that reduced vitamin intake did not translate directly into low plasma vitamin concentrations. Retinol values were generally within the reference range, and several patients had very high, nearly toxic retinol concentrations. Some patients with high retinol showed symptoms compatible with hypervitaminosis A, including hair loss, dry or fissured lips, and dermatitis, whereas patients with low-normal retinol sometimes showed skin changes such as dryness, roughness, papular eruptions, and follicular hyperkeratosis.

### 3.13. Vitamin D

Interpretive summary (cohort and marker variability): Vitamin D was frequently low in severe hospitalized AN cohorts [48,49], while other studies assessed 25-hydroxyvitamin D in different settings and seasons [55,56,58]. An AN/ARFID refeeding analysis [83] addressed phosphate nadir rather than treatment benefit. Evidence is observational and strongly affected by supplementation, season, assay threshold, and clinical severity.

Low serum 25-hydroxyvitamin D [25(OH)D] was common in some severely malnourished AN cohorts but was not universal. Among 374 hospitalized adults, 54.2% were classified as deficient [48]; among 354 extremely underweight adults, deficiency affected 117 of 186 tested patients (62.9%) [49]. Low vitamin D was also reported in a small male AN cohort [44]. These estimates depend on assay cutoffs, selection of tested patients, season, supplementation, and illness severity, and cannot be pooled without harmonization.

Other inpatient studies reported lower proportions with low vitamin D. In a mixed severe restrictive eating-disorder cohort, 25% had low 25(OH)D [82], while a separate AN/ARFID analysis classified 7% as deficient and 23% as insufficient [83]. The latter study did not establish vitamin D as an independent predictor of refeeding phosphate nadir. Differences in diagnosis, cutoffs and supplementation complicate comparisons.

Several adolescent and young adult cohorts also showed clinically meaningful vitamin D inadequacy. Modan-Moses et al. [55] reported a mean 25(OH)D concentration of 24.4 ± 7.4 ng/mL in 87 adolescent inpatients with eating disorders, including 64 patients with anorexia nervosa. Using pediatric criteria, 8% were vitamin D deficient and 23% insufficient, meaning that about 30% had values below 20 ng/mL. The authors also emphasized that only 17% had 25(OH)D levels above 32 ng/mL, suggesting that if a stricter threshold for optimal skeletal health were applied, most patients would have non-optimal vitamin D status. This study also demonstrated a clear seasonal effect: winter values were significantly lower than summer values, with deficiency occurring only in winter, supporting the importance of sunlight exposure as a determinant of vitamin D status in eating-disorder populations. In the German ANDI multicenter cohort [94], 93 female adolescents with anorexia nervosa had a mean 25(OH)D of 60.3 ± 19.0 nmol/L at admission, but 30% were deficient below 50 nmol/L. Vitamin D status remained relatively stable at discharge, but worsened at one-year follow-up, when 45% were deficient.

Some studies, however, found preserved or even relatively adequate vitamin D status in anorexia nervosa. Pettersson et al. [77], in a 3-year follow-up of young women previously hospitalized for severe restrictive anorexia nervosa, found a mean serum 25(OH)D of 88.8 nmol/L, with only 20% below 50 nmol/L; the anorexia nervosa group even had higher vitamin D levels than the healthy reference group, probably because several participants used calcium/vitamin D or multivitamin-mineral supplements. DiVasta et al. [56] similarly found that malnourished adolescent and young adult females with anorexia nervosa did not have lower baseline 25(OH)D than controls: values were 41 ± 11 ng/mL in anorexia nervosa and 38 ± 11 ng/mL in controls, with vitamin D insufficiency present in 17% and 25%, respectively. This study is informative because it tested the bioavailability of vitamin D2 after a 50,000 IU oral dose of ergocalciferol and found that severe malnutrition did not meaningfully impair overall vitamin D2 bioavailability, although the early 6 h vitamin D2 peak was lower in anorexia nervosa. Interestingly, despite preserved 25(OH)D, patients with anorexia nervosa had lower 1,25-dihydroxyvitamin D at baseline, suggesting that vitamin D stores and active vitamin D hormone regulation may not always move in parallel. Likewise, study [102] found no evidence of vitamin D deficiency in young Swedish women with anorexia nervosa: total 25(OH)D and free 25(OH)D were not lower than in controls, only two patients had total 25(OH)D below 50 nmol/L, and none were receiving vitamin D supplementation. The authors therefore concluded that vitamin D deficiency was unlikely to explain the reduced bone mineral density observed in that cohort.

Several studies reported intermediate or context-dependent findings. In Schorr et al. [42], men with anorexia nervosa had a mean 25(OH)D of 28.3 ± 16.2 ng/mL, and vitamin D deficiency below 20 ng/mL was present in 24% of men with anorexia nervosa and 21% of men with atypical anorexia nervosa. At the same time, none of the small ARFID groups met the criteria for deficiency. Nagata et al. [86] showed that low vitamin D status can also be prominent in atypical anorexia nervosa: among hospitalized males with atypical anorexia nervosa, mean 25(OH)D was 28.8 ± 7.4 ng/mL, 42.1% had insufficiency, and 10.5% had deficiency, so 52.6% had low vitamin D despite not being underweight by conventional BMI criteria. This supports the broader point that recent weight loss, restrictive intake, and illness physiology may be more informative than BMI alone. In the multicentric study [90], mean vitamin D was 30.4 ± 14.14 ng/mL and was not significantly correlated with BMI, while in Caldiroli et al. [117], mean vitamin D was 24.69 ± 8.58 ng/mL and was numerically lower in moderate-to-extreme anorexia nervosa than in mild anorexia nervosa, but this difference was not statistically significant.

The strongest evidence linking vitamin D deficiency to skeletal and endocrine consequences comes from studies focused on bone metabolism. Urano et al. [60] found that the mean 25(OH)D was lower in anorexia nervosa than in controls and was especially low in binge-eating/purging anorexia nervosa. Using 20 ng/mL as the deficiency threshold, vitamin D deficiency occurred in 51% of restricting-type patients and 72% of binge-eating/purging-type patients, compared with 33% of controls. The active form, 1,25-dihydroxyvitamin D, was also reduced in anorexia nervosa, particularly in the binge/purge subtype. However, serum calcium remained normal, and serum phosphorus was mildly higher than in controls, indicating that vitamin D deficiency did not necessarily result in overt hypocalcemia. Gatti et al. [57] reported that untreated anorexia nervosa was associated with a high frequency of low vitamin D status: 16.9% had severe deficiency below 12 ng/mL, 19.1% had deficiency between 12 and below 20 ng/mL, and 22.4% had insufficiency between 20 and below 30 ng/mL. Therefore, 58.4% had 25(OH)D below 30 ng/mL. Importantly, patients with 25(OH)D below 20 ng/mL had higher parathyroid hormone than those with higher vitamin D levels, suggesting a biologically meaningful endocrine response that may contribute to bone resorption and skeletal fragility. In study [107], 41% of women with anorexia nervosa had vitamin D deficiency below 20 ng/mL, but serum calcium, phosphorus, ionized calcium, and PTH were largely preserved, and vitamin D was not a major determinant of bone mineral density in regression models.

### 3.14. Vitamin E

Interpretive summary (circulating versus functional markers): Plasma vitamin E was usually within range in the 153-patient AN cohort [31], while selected reports evaluated other tocopherol fractions or compartments [53,59]. Assay methods, lipid transport and supplementation history may change apparent status; these data do not establish a consistent clinically important vitamin E deficit.

Vitamin E was usually preserved in plasma or serum, although compartment-specific or severe-case abnormalities were reported. In the large retrospective cohort of 153 adult female patients with anorexia nervosa, mean plasma vitamin E concentrations were mostly within the normal range, and overt vitamin E deficiency was rare, occurring in only 1.5% of patients [31]. However, this study also found that vitamin E concentrations differed by anorexia nervosa subtype: vitamin E was significantly lower in the restrictive subtype than in the binge–purging subtype, even though actual deficiency remained uncommon in both groups [31]. This suggests that restrictive anorexia nervosa may be associated with relatively lower vitamin E availability, probably reflecting lower fat intake or lower intake of vitamin-E-containing foods, but that frank biochemical depletion is not frequent when vitamin E is assessed by standard plasma measures.

A similar pattern of largely preserved vitamin E status was reported in the 3-year follow-up study by Pettersson et al. [77]. In young women previously hospitalized for severe restrictive anorexia nervosa, serum tocopherol was adequate on average, with a mean of 32 µmol/L and a range of 21–56 µmol/L, and no participant had values below the laboratory reference range [77]. Dietary vitamin E intake averaged 12 mg/day, which was above the recommended intake of 8 mg/day; nevertheless, 25% of participants consumed less than the recommended amount, and 11% were below the lower intake level [77]. Thus, although serum vitamin E concentrations were maintained, the intake data indicated that a subgroup remained nutritionally vulnerable.

Langan and Farrell [101] provided a more detailed analysis of vitamin E status by measuring plasma tocopherol isomers in 17 hospitalized patients with anorexia nervosa. Alpha-tocopherol, the main biologically active form of vitamin E, was not significantly lower in patients with anorexia nervosa than in controls; in fact, mean alpha-tocopherol was 1.21 ± 0.51 mg/dL in the anorexia nervosa group compared with 1.01 ± 0.16 mg/dL in controls [101]. Total tocopherol was also not significantly different between groups, with values of 1.39 ± 0.51 mg/dL in anorexia nervosa and 1.35 ± 0.17 mg/dL in controls [101]. However, the beta-plus-gamma-tocopherol fraction was significantly reduced in patients with anorexia nervosa, at 0.16 ± 0.06 mg/dL compared with 0.25 ± 0.51 mg/dL in controls [101]. This distinction is clinically relevant because beta- and gamma-tocopherols are derived from food sources, whereas alpha-tocopherol is commonly present in supplements.

In adolescents with anorexia nervosa, vitamin E also appeared relatively preserved in the study [65]. Patients were assessed at admission and again after short-term inpatient nutritional rehabilitation. Vitamin E concentrations were reported as normal and did not change significantly during hospitalization [65]. This finding fits the broader pattern of the study, in which most vitamins, apart from folate, remained within reference ranges despite marked undernutrition. However, the absence of a major change during refeeding also suggests that short-term weight gain does not necessarily produce large shifts in circulating vitamin E, especially when baseline values are already within normal limits. Dowd et al. [115] likewise reported no significant vitamin E depletion in anorexia nervosa. Mean vitamin E was 961 ± 292 µg/100 mL in patients and 1031 ± 172 µg/100 mL in controls [115]. Therefore, even in patients with clear anthropometric evidence of wasting, including reduced body weight, triceps skinfold, mid-arm circumference, and muscle circumference, vitamin E was not systematically reduced [115].

Philipp et al. [63] also found that plasma tocopherol concentrations were generally within the reference range in hospitalized women with anorexia nervosa and bulimia nervosa, even though dietary intake was often inadequate, particularly in anorexia nervosa. The authors observed that tocopherol intake was strongly correlated with fat intake, as expected because vitamin E is lipid-soluble and is mainly supplied through fat-containing foods [63]. Thus, low dietary fat intake in anorexia nervosa may create a risk for reduced vitamin E supply over time, even when plasma tocopherol remains normal at the time of measurement. In the outpatient study by Mira, Stewart, and Abraham [53], vitamin E deficiency was again not the dominant pattern. Median plasma vitamin E was 16.5 µmol/L in controls, 19.5 µmol/L in eating-disorder patients not taking supplements, and 21.0 µmol/L in supplemented eating-disorder patients [53]. The gradient was statistically significant, with the highest values observed in supplemented patients [53].

Rock and Vasantharajan [128] reported a more complex pattern. In 13 patients with eating disorders, most of whom had met criteria for anorexia nervosa at some point, the mean plasma alpha-tocopherol was 23.68 ± 6.34 µmol/L. However, two patients, representing 15% of the sample, had low alpha-tocopherol concentrations ranging from 11.8 to 16.3 µmol/L [128]. Importantly, alpha-tocopherol was positively associated with cholesterol and total lipids, and gamma-tocopherol was also associated with total lipids [128]. This indicates that the interpretation of vitamin E in anorexia nervosa is complicated by altered lipid metabolism, as tocopherols circulate within lipoprotein fractions. Vitamin E interpretation in anorexia nervosa is therefore complicated by altered lipid metabolism. Absolute plasma vitamin E values may therefore underestimate or overestimate true tissue vitamin E status depending on lipid concentrations. The authors also found associations among alpha-tocopherol, albumin, cholesterol, total lipids, relative body weight, and BMI, reinforcing the idea that vitamin E biomarkers should be interpreted alongside lipid and protein-transport markers rather than in isolation [128].

Clinically important vitamin E deficiency was reported mainly in more severe or highly selected contexts. In the case report [156], a 28-year-old woman with long-standing anorexia nervosa, extreme emaciation, and parenteral nutrition developed severe selenium-deficiency myopathy. In this patient, serum vitamin E was also markedly reduced, at 0.35 mg/dL, below the normal range of 0.75–1.41 mg/dL, despite vitamin E being added to the parenteral nutrition [156]. The combination of selenium deficiency and vitamin E deficiencies was interpreted as a combined antioxidant-deficiency state that may have contributed to skeletal-muscle oxidative injury [156]. Although this case cannot estimate prevalence, it demonstrates that severe anorexia nervosa, especially when complicated by prolonged malnutrition, parenteral nutrition, or inadequate antioxidant replacement, can be associated with clinically meaningful vitamin E depletion.

Moyano et al. [59] provided additional evidence that vitamin E abnormalities may be more detectable when erythrocyte tocopherol is measured instead of standard plasma tocopherol. In 82 female adolescents with anorexia nervosa, erythrocyte alpha-tocopherol was significantly lower than reference values, with a median of 22.3 nmol/g protein compared with 25.3 nmol/g protein in healthy adolescents [59]. Moreover, 21% of patients with anorexia nervosa had tocopherol concentrations below the reference interval [59]. This suggests that vitamin E insufficiency may be underestimated when only plasma vitamin E is measured, particularly because erythrocyte tocopherol may better reflect membrane antioxidant protection. Moyano et al. interpreted the reduced tocopherol as potentially reflecting insufficient antioxidant-vitamin intake, increased tocopherol consumption due to oxidative stress, or both [59].

### 3.15. Vitamin K

Interpretive summary (limited functional-marker evidence): The central comparative study [60] enrolled 54 amenorrheic women with AN and 15 controls and used serum undercarboxylated osteocalcin as a functional marker related to vitamin K-dependent carboxylation. This is distinct from direct serum vitamin K measurement and is influenced by bone metabolism; it cannot by itself establish treatment efficacy.

Direct evidence on vitamin K status in AN was sparse. Urano et al. [60] compared 54 amenorrheic women with AN and 15 healthy controls using serum undercarboxylated osteocalcin (ucOC), a functional indicator of vitamin K-dependent bone protein carboxylation rather than a direct vitamin K concentration. Mean ucOC was higher in AN (3.9 versus 2.3 ng/mL). Using ucOC ≥ 4.5 ng/mL, abnormal values occurred in 17% of restricting-type and 40% of binge-eating/purging-type AN; mean ucOC was also higher in the latter subtype (5.0 versus 2.9 ng/mL). These results suggest subtype-related differences in a bone-associated functional marker but cannot independently establish depleted vitamin K stores, mechanisms, or clinical benefit from supplementation.

### 3.16. Vitamin B1

Interpretive summary (functional status and clinical phase): A controlled study assessed erythrocyte transketolase activation in 37 patients meeting all or some AN criteria and 50 controls [61], while other reports used whole-blood or clinical markers. Different assays and nutritional-treatment exposures explain part of the discrepancy. Thiamine requirements during refeeding are a clinical management issue distinct from the prevalence of pre-treatment biochemical deficiency.

Thiamine status was heterogeneous and depended strongly on assay type, severity, treatment stage, and prior supplementation.

In the large cohort of severely malnourished hospitalized adults described in the study [48], vitamin B1 deficiency was present in 15% of patients with anorexia nervosa. This finding places thiamine deficiency below zinc, vitamin D, copper, and selenium deficiencies in frequency, but still identifies it as a relevant abnormality in a clinically high-risk inpatient population. Vitamin B1 levels did not differ significantly between restricting and binge-eating/purging subtypes, suggesting that deficiency was not specifically driven by purging but may reflect severe malnutrition, chronic restriction, prolonged illness, or inconsistent intake.

A different pattern was observed in adolescents with anorexia nervosa in a study [65]. In this sample of 61 patients aged 10–19 years, vitamin B1 concentrations were within the reference range both at admission and after short-term inpatient nutritional rehabilitation. Mean thiamine increased significantly from 51.2 µg/L at admission to 57.3 µg/L at discharge, while both values remained within the reference interval of 35–71 µg/L. Thus, unlike folate and zinc, which were clearly abnormal and persistent problems in this adolescent cohort, vitamin B1 did not show evidence of frank deficiency. This may reflect several factors, including the younger age of the patients, the relatively shorter mean illness duration, homeostatic preservation of some biochemical markers, or the possibility that short-term nutritional rehabilitation was sufficient to maintain or improve circulating thiamine levels.

Functional evidence of thiamine insufficiency was reported in the study by Philipp et al. [63], which assessed vitamin status in hospitalized women with anorexia nervosa and bulimia nervosa. This study is important because vitamin B1 status was evaluated not only through dietary intake or circulating vitamin concentrations, but also through erythrocyte transketolase activation after the addition of thiamine pyrophosphate. Since transketolase is thiamine-dependent, increased activation after coenzyme addition suggests poorer intracellular thiamine saturation. Most patients appeared to have sufficient vitamin B1 status, but several showed enzyme-activation patterns compatible with possible B-vitamin insufficiency, including vitamin B1. The study therefore supports the idea that plasma vitamin levels may appear normal while functional intracellular markers reveal subclinical deficiency.

The strongest evidence for clinically relevant thiamine deficiency comes from a study [61], which specifically focused on vitamin B1 status in anorexia nervosa. Winston and colleagues assessed 37 patients with anorexia nervosa using erythrocyte transketolase activation, a functional assay of thiamine status, rather than relying only on serum thiamine concentration. Patients with anorexia nervosa had much higher mean transketolase activation than non-supplemented controls: 13.0% versus 1.7%, indicating poorer thiamine-dependent enzyme saturation. Using a cutoff of more than 12.6% activation, defined as more than two standard deviations above the non-supplemented control mean, 14 of 37 patients (approximately 38%) had biochemical evidence of thiamine deficiency. Even when a stricter cutoff of more than 32% activation was applied, 7 of 37 patients, or approximately 19% still met the criteria for deficiency. No control participants exceeded either threshold.

In contrast, study [62] found preserved thiamine status in a very severely malnourished adult sample. This study included 24 adults with extreme anorexia nervosa admitted with a mean BMI of only 12.54 kg/m^2^ and an ideal body weight below 70%. Whole-blood thiamine diphosphate, the biologically active form of thiamine, was measured by high-performance liquid chromatography. Despite the extreme degree of malnutrition, mean admission thiamine diphosphate was 134.46 ± 35.16 nmol/L, within the reference range of 70–180 nmol/L, and was not significantly different from controls. No participant showed clinical concern for Wernicke’s encephalopathy or other manifestations of thiamine deficiency. Subtype analysis also did not show a significant difference between restricting and binge-eating/purging anorexia nervosa, although the binge-eating/purging group had numerically lower thiamine diphosphate levels. However, interpretation of this study requires caution because all patients received 200 mg of oral thiamine on arrival, and the admission blood sample was usually drawn the next morning, more than 12 h after the first dose. Therefore, the measured “admission” value may have partly reflected early supplementation rather than completely untreated baseline thiamine stores.

### 3.17. Vitamin B2

Interpretive summary (multiple biological matrices): The 17-patient versus 17-control study [64] simultaneously assessed erythrocyte riboflavin, plasma cofactors and urinary metabolites during a feeding program and reported different directions across measures. Small sample size, thyroid adaptation and active treatment mean that a single low/normal/high label would misrepresent this nutrient.

Philipp et al. [63] assessed riboflavin using erythrocyte glutathione-reductase activation by flavin adenine dinucleotide (FAD) in a mixed eating-disorder sample. Functional abnormalities were identified in some participants despite largely preserved circulating vitamin measures. Mild dermatological complaints were described but were not specific for riboflavin deficiency; AN-specific conclusions are limited by the mixed sample.

In a small mixed eating-disorder cohort (*n* = 13), four participants (31%) had abnormal erythrocyte glutathione-reductase activation consistent with functional riboflavin insufficiency [128]. Those with abnormal results had lower relative body weight, but sample size and incomplete diagnostic separation preclude a generalizable AN-specific prevalence estimate.

A case–control study of 17 adolescent girls with AN and 17 controls measured riboflavin and its coenzymes across compartments [64]. Plasma FAD was lower in AN (45.6 ± 23.9 versus 78.5 ± 54.3 nmol/L), whereas plasma riboflavin and erythrocyte FAD/FMN were not significantly different; erythrocyte-free riboflavin was higher. The discordance indicates that a single circulating measure cannot capture all aspects of riboflavin metabolism.

### 3.18. Vitamin B6

Interpretive summary (functional versus circulating measures): Vitamin B6 assessments include circulating concentrations and enzyme-based functional indices from selected small or mixed eating-disorder samples [63,65]. Apparent preservation and functional insufficiency may coexist, and illness stage, protein binding and supplementation are incompletely controlled. AN-specific inference is therefore limited.

Vitamin B6 was usually preserved in circulation, but functional insufficiency occurred in a subset of more underweight or clinically severe patients. Importantly, this study did not identify clinically relevant vitamin B6 deficiency, and the authors emphasized that, apart from folic acid, no absolute vitamin deficiency was detected in this adolescent cohort with anorexia nervosa [65]. This suggests that circulating vitamin B6 may remain preserved in many young patients with relatively short illness duration, possibly because of homeostatic regulation, selective dietary patterns, previous supplementation, or the fact that overt biochemical depletion may require longer or more severe malnutrition.

Functional erythrocyte enzyme-activation testing in a mixed AN/bulimia sample identified possible B6 insufficiency in some participants despite often preserved plasma vitamin values [63]. Dermatological manifestations were nonspecific, and the mixed sample prevents AN-specific prevalence estimates.

Similarly, Rock and Vasantharajan found that vitamin B6 abnormalities were present in a clinically meaningful subset of low-weight patients with eating disorders. Using erythrocyte glutamic pyruvic transaminase activity with and without added pyridoxal phosphate, they reported abnormal vitamin B6 indices in 4 of 13 patients (31%) [128].

Mira, Stewart, and Abraham also assessed vitamin B6 functionally by measuring the percentage increase in erythrocyte aspartate transaminase activity after the addition of pyridoxal-5-phosphate. In their outpatient eating-disorder sample, which included anorexia nervosa, bulimia nervosa, and atypical eating disorders, non-supplemented patients did not differ significantly from healthy controls. Median activation was 53% in controls and 56% in non-supplemented eating-disorder patients, suggesting no generalized vitamin B6 deficiency across the whole outpatient group. However, supplemented eating-disorder patients showed lower activation, with a median of 42%, indicating better vitamin B6 activity, and this group differed significantly from both controls and non-supplemented patients. The most clinically relevant finding for anorexia nervosa was that low actual body weight significantly predicted upper-quartile vitamin B6 activation. Because higher activation reflects poorer vitamin B6 status, this indicates that the most underweight patients were more likely to show functional evidence of vitamin B6 insufficiency [53]. Thus, although vitamin B6 deficiency was not a general feature of all eating-disorder patients, low body weight appeared to identify a higher-risk subgroup.

One smaller anorexia nervosa study reported the opposite pattern, showing higher rather than lower vitamin B6 concentrations in patients. In this study of 11 women with anorexia nervosa and 12 healthy controls, vitamin B6 levels were significantly higher in the anorexia nervosa group, with a mean of 37.2 ± 24.7 nmol/L in patients compared with 14.7 ± 6.3 nmol/L in controls [166]. The same study found no deficiency of folic acid or vitamin B12, and the authors explicitly stated that vitamin B6, vitamin B12, and folic acid were higher or tended to be higher in anorexia nervosa rather than reduced. This finding reinforces the idea that vitamin B6 status in anorexia nervosa cannot be assumed to mirror caloric restriction in a simple linear way. Circulating levels may be normal or elevated, whereas functional indices may still detect suboptimal intracellular status in some patients.

### 3.19. Vitamin B9

Interpretive summary (serum versus erythrocyte folate): The 153-patient AN cohort [31] reported some low circulating folate, while male and other severe cohorts measured red-cell folate or related one-carbon indicators [44,46,66]. Different sample matrices, recent intake and treatment exposure preclude treating folate results as a single comparable prevalence estimate.

Folate status was heterogeneous, with preserved circulating values in many adults but clinically relevant deficiency in selected cohorts, particularly when red-cell folate was assessed. For example, in a cohort of severely malnourished male patients with anorexia nervosa, red blood cell folate was available for seven patients and averaged 840 ± 456 ng/mL, with values ranging from 320 to 1654 ng/mL against a reference range of 280–790 ng/mL; therefore, no folate deficiency was identified despite profound protein-energy malnutrition [44]. Similarly, in another cohort of patients with severe anorexia nervosa, red blood cell folate was measured in 25 patients and averaged 653 ng/mL, within the reference range, and the authors explicitly reported that no patient had folate or vitamin B12 deficiency, even though anemia was very common [46]. These findings are important because they show that hematological abnormalities in anorexia nervosa cannot automatically be attributed to classical megaloblastic vitamin deficiency; in severe starvation, bone marrow suppression, low BMI, and refeeding-related physiological changes may be more important explanations than folate depletion alone.

Nevertheless, other studies found that folate deficiency is clinically relevant in a meaningful subgroup of patients. In one large cohort of 374 hospitalized adults with severe anorexia nervosa, vitamin B9 deficiency was present in 8.9% of patients. This prevalence was lower than that observed for zinc, vitamin D, copper, selenium, and thiamine, but it still indicates that folate deficiency is not rare in medically compromised anorexia nervosa [48]. Another retrospective study of 153 adult female patients found that mean plasma folate concentrations were mostly within the normal ranges at the population level, but individual-level deficits were frequent: vitamin B9/folate deficiency was present in 15.9% of patients, making it one of the most common vitamin deficiencies after vitamin A deficiency in that cohort [31]. In that study, folate deficiency was numerically more frequent in the binge–purging subtype than in the restrictive subtype (18.9% versus 14.1%), although this difference was not statistically significant. This suggests that purging behavior may contribute to vulnerability in some patients, but anorexia nervosa subtype alone is not a sufficiently reliable predictor of folate status.

An adolescent inpatient study [65] measured both serum and red-cell folate before and after rehabilitation. Low serum folate increased from 21.9% at admission to 34.4% at discharge, whereas low erythrocyte folate was present in 54.0% and 51.6%, respectively. This matrix-specific finding suggests that short-term weight restoration does not necessarily normalize longer-term folate markers; however, supplementation and treatment-stage differences restrict causal interpretation.

Several investigations reported preserved serum folate despite low dietary intake. A three-year follow-up found normal serum folate in all assessed patients [77], while a mixed eating-disorder investigation showed frequently inadequate folate intake but generally preserved plasma concentrations [63]. Other adult or mixed-sample reports described broad variability without a uniform depletion pattern [90,112,117,165,166].

Taken together, baseline folate status cannot be inferred from BMI or serum values alone. Results depend on age, supplementation, treatment stage and whether serum or erythrocyte folate is measured. The evidence supports interpreting abnormal results in their clinical context, rather than assuming universal deficiency or establishing an untested supplementation strategy.

### 3.20. Vitamin B12

Interpretive summary (pre-treatment serum B12): Baseline AN inpatient cohorts found overt deficiency uncommon [31,48]; a retrospective 71-woman AN-specific study before nutritional therapy observed low B12 in 2 and elevated B12 in 22 patients [67]. Serum cobalamin is not equivalent to intracellular availability, and liver function can confound high values. These findings cannot establish a need for or against a specific supplement protocol.

Vitamin B12 deficiency was uncommon; normal or elevated circulating B12-related markers were more typical. In the large cohort of extremely malnourished hospitalized adults in the study [48], vitamin B12 deficiency was present in only 4.7% of patients, making it one of the least frequent deficiencies among the measured micronutrients. Nevertheless, vitamin B12 concentrations were significantly lower in the binge-eating/purging subtype than in the restricting subtype, suggesting that although frank deficiency was rare, subtype-related differences in B12 status may exist in severe anorexia nervosa. A similar pattern was reported in study [31], in which mean plasma vitamin B12 concentrations were mostly within normal ranges, and vitamin B12 deficiency was found in only 2.2% of patients. Thus, in both relatively large adult cohorts, vitamin B12 depletion was not a dominant biochemical abnormality, even though many patients had at least one vitamin or trace-element deficiency. Study [49], which examined adults with extremely severe anorexia nervosa and a mean BMI of only 12.2 kg/m^2^, confirmed this general conclusion: vitamin B12 deficiency was the least frequent deficiency among the measured nutrients, occurring in 12 of 281 tested patients, or 4.3%. Therefore, even in very advanced starvation, vitamin B12 deficiency appeared much less frequent than vitamin D, zinc, copper, selenium, or folate deficiency.

Several studies even reported normal or elevated vitamin B12 values rather than deficiency. In the small cohort of severely malnourished males in the study [44], vitamin B12 was available for nine patients and showed a mean value of 847 ± 238 pg/mL, with a range of 431–1298 pg/mL; the authors explicitly stated that none of the patients had vitamin B12 or folate deficiency. Similarly, study [46], which included 53 adults with severe anorexia nervosa admitted for medical stabilization, found no folate or vitamin B12 deficiency among those tested, despite a very high prevalence of anemia. Vitamin B12 was measured in 27 patients and had a median value of 717 pg/mL, with an interquartile range of 448–1095 pg/mL. This is clinically important because it indicates that anemia in severe anorexia nervosa is often not a classical megaloblastic anemia caused by B12 or folate depletion, but may instead reflect starvation-mediated bone marrow suppression or broader hematopoietic dysfunction. Similar preservation of B12 was seen in adolescent or mixed cohorts. In study [65], vitamin B12 remained within the reference range both at admission and discharge, although it decreased significantly from 566.7 pg/mL to 453 pg/mL during short-term inpatient rehabilitation. This decline suggests that B12 may change dynamically during refeeding, but the values did not indicate frank deficiency. In study [116], adolescents with restrictive anorexia nervosa had a median vitamin B12 concentration of 747 ng/L compared with 633 ng/L in controls, with no significant difference and no evidence that B12 deficiency was a major feature of the cohort. Study [105] also reported mean serum cobalamin within the normal range, 500 ± 245 pmol/L, and did not identify B12 deficiency as a prominent biochemical abnormality.

A particularly important finding emerging from several studies is the paradoxical elevation of vitamin B12 in anorexia nervosa. Study [66], which focused on one-carbon metabolism, found that women with active anorexia nervosa had significantly higher plasma B12 concentrations than women without eating disorders, with mean values of 702.67 versus 371.53, respectively. Women remitted from anorexia nervosa also had elevated B12, with a mean value of 641.50, suggesting that the abnormality may not be restricted to the acute underweight phase. Within the active anorexia nervosa group, lower BMI was associated with higher B12, indicating that elevated circulating B12 may track illness severity rather than nutritional adequacy. Study [90] similarly found that the mean plasma vitamin B12 was within the normal adult reference range, 529.4 ± 325.68 pg/mL, but higher B12 was significantly associated with lower BMI. The correlation between BMI and B12 was negative and statistically significant, and this association remained significant in regression analysis. Study [117] showed the same direction of effect: patients with moderate-to-extreme anorexia nervosa had higher mean vitamin B12 levels than patients with mild anorexia nervosa, 573.38 ± 326.91 pg/mL versus 437.00 ± 129.80 pg/mL, although this difference reached only a trend level. Importantly, this study excluded patients taking vitamin B12 supplements, making it less likely that elevated values were attributable simply to supplementation.

The most detailed evidence for elevated B12 as a pathophysiological marker rather than a nutritional marker comes from studies examining liver-related mechanisms. Study [89] measured total vitamin B12, holotranscobalamin, methylmalonic acid, and a combined B12-status marker in 77 underweight female patients with acute anorexia nervosa and 63 healthy controls, with reassessment after short-term weight restoration. The study did not show classical B12 deficiency. Instead, acute anorexia nervosa was characterized by higher holotranscobalamin, the biologically active B12 fraction, with a median of 73 pmol/L compared with 54 pmol/L in controls. Total B12 was also numerically higher in acute anorexia nervosa, although the clearest acute difference concerned holotranscobalamin. Methylmalonic acid decreased after weight restoration, while the combined B12 marker did not differ significantly between acute anorexia nervosa and controls. This pattern suggests that elevated circulating B12-related markers may reflect altered metabolism, hepatic release, or starvation-related liver involvement rather than true B12 excess or better nutritional status. Study [152] further supports this interpretation even more directly. In 70 female adolescents with restrictive-type anorexia nervosa, the mean plasma vitamin B12 was 643 ± 333 pg/mL, close to the upper limit of the reference range. Of 70 patients, 27 (38.5%) had B12 values above the upper normal limit, while none had values below the lower limit. The authors interpreted frequent hypercobalaminemia not as evidence of good nutritional reserve, but as a possible signal of liver involvement, because vitamin B12 is stored in the liver and may be released into circulation when hepatocytes are damaged. The same study found that high-B12 patients also had higher folate values, although folate deficiency and folate elevation were both uncommon compared with B12 elevation.

Clinically relevant functional B12 deficiency was described despite initially normal serum B12 in a patient with neurological symptoms [153]. Other severe AN cases had normal or high B12 alongside macrocytosis, scurvy or electrolyte complications [68,85,110,149]. These isolated observations reinforce that serum B12 alone does not resolve the cause of hematological or neurological findings, but they cannot quantify deficiency frequency.

### 3.21. Vitamin C

Interpretive summary (clinical extremes versus cohort averages): Circulating vitamin C was not uniformly low in cohort studies [49,53], but reports of clinically manifest scurvy in severely restrictive AN [68,69] identify a potentially serious minority phenotype. Case reports are sentinel observations, not prevalence evidence or controlled tests of a supplementation regimen.

Vitamin C was generally preserved in group studies but could become severely deficient in patients with extreme or highly selective restriction.

In adolescent inpatients, vitamin C concentrations were generally within the laboratory range at admission and discharge [65]. A controlled study also reported broadly similar leukocyte vitamin C in AN and healthy controls despite marked anthropometric malnutrition [115]. Thus, systemic underweight did not consistently correspond to biochemical vitamin C depletion in these cohorts.

However, other studies have shown that vitamin C may be among the more vulnerable water-soluble vitamins in selected patients with eating disorders. Philipp et al. [63], who assessed women with anorexia nervosa and bulimia nervosa using dietary records and biochemical vitamin measurements, reported that plasma concentrations of most vitamins investigated were generally within reference ranges, but vitamin C was an exception because reduced ascorbic acid concentrations were found in several patients. Importantly, these low values were not necessarily accompanied by overt clinical symptoms of scurvy. The study also highlighted substantial interindividual variability in plasma ascorbic acid concentrations, which the authors linked to the highly variable eating habits of patients with eating disorders. Some patients, especially those with anorexia nervosa, may consume relatively large amounts of vegetables, salads, and fruits, thereby maintaining or even increasing vitamin C intake despite severe restriction of energy, fat, protein, or other nutrients. The positive association between ascorbic acid intake and plasma ascorbic acid concentration in this study suggests that vitamin C may reflect recent dietary intake more directly than some fat-soluble vitamins or vitamins with larger body stores [63].

The clearest evidence of clinically important vitamin C deficiency came from case reports and from the most severely malnourished clinical cohorts. In the large adult cohort of 354 patients with extremely severe anorexia nervosa [49], the mean BMI at admission was only 12.2 ± 1.6 kg/m^2^, and many patients were in a state of advanced, chronic, marasmic undernutrition. In this population, cutaneous lesions consistent with scurvy were reported in 4 of 354 patients, corresponding to 1.1%, and these patients had low blood vitamin C levels. Although this prevalence was low compared with deficiencies such as vitamin D, zinc, copper, selenium, or folate, the finding is clinically important because it shows that frank vitamin C deficiency can occur in the most medically compromised patients with anorexia nervosa. The low prevalence also indicates that scurvy is not a common biochemical finding across all patients with anorexia nervosa, but rather a severe complication that may emerge in a vulnerable subgroup with extreme malnutrition, inadequate intake, and medical instability [49].

A patient with severe restrictive AN developed purpura, impaired wound healing and arthralgia compatible with scurvy; a low urinary ascorbate response after a loading dose supported depleted vitamin C status [110]. The clinical presentation occurred despite some fruit and vegetable intake, indicating that highly restricted total intake may still be insufficient.

A second adolescent case report [68] described scurvy in a 16-year-old female patient with restrictive-type anorexia nervosa. The patient had restricted food intake for 7–8 months, had experienced recent complete food refusal, and had adopted a vegetarian diet in the context of wanting to become “healthy.” Her serum vitamin C was clearly below the reference range: 21 µmol/L (0.23 mg/dL), compared with a reported reference interval of 25–114 µmol/L (0.28–1.29 mg/dL). The biochemical deficiency was accompanied by characteristic dermatological signs, confirming clinically visible scurvy. Vitamin A and vitamin B12 were reported as normal, indicating that the skin findings were not better explained by these other vitamin results. This case reinforces the idea that vitamin C deficiency in anorexia nervosa may be highly specific and clinically prominent even when not all measured vitamins are abnormal [68].

Another severely malnourished adolescent with restrictive AN developed petechiae and ecchymoses suggestive of scurvy that improved after vitamin C administration [69]. Because multivitamin treatment had preceded assessment, the diagnosis was clinical rather than based on an untreated plasma concentration. This limitation is relevant when interpreting measurements collected after supplementation.

## 4. Discussion

The present synthesis shows that biochemical evidence for frank micronutrient deficiency in AN is inconsistent. Although dietary restriction clearly creates a risk of inadequate intake, a circulating micronutrient concentration is not a direct inventory of total-body stores. Serum and plasma biomarkers can be modified by inflammation, hydration and plasma-volume changes, carrier-protein concentrations, hepatic and renal function, redistribution between compartments, recent intake, and supplementation; accordingly, nutritional biomarkers should be interpreted in their clinical and metabolic context [28,29,30]. This distinction is particularly important in AN, where starvation, purging behaviors, altered body composition, and the transition from catabolism to anabolism during refeeding can change biochemical values without producing a simple one-to-one relationship between intake and blood concentration [8,10,16]. The Discussion therefore interprets the findings as context-dependent physiological patterns rather than as evidence that a normal blood value necessarily excludes tissue depletion or functional insufficiency.

The recent World Federation of Societies of Biological Psychiatry (WFSBP) consensus statement on candidate biomarkers for anorexia nervosa by Himmerich et al. [167] evaluated vitamins, coenzymes, and nutritional markers within a broader framework of biological indicators, emphasizing the heterogeneity of existing findings and the lack of sufficiently validated diagnostic and prognostic biomarkers. The present scoping review complements and extends this consensus by providing a detailed, nutrient-specific synthesis of 136 primary studies examining vitamins, minerals, trace elements, and electrolytes in anorexia nervosa. Unlike the broader biomarker-focused consensus statement, our review distinguishes findings according to biological matrix, study design, illness severity, clinical subtype, and treatment stage, particularly separating baseline micronutrient status from abnormalities emerging during purging, medical instability, or refeeding. Our findings support the recognition of selenium, zinc, vitamin D, and selected electrolyte disturbances as potentially relevant indicators of nutritional and medical vulnerability, while demonstrating that other biomarkers, including vitamin B12, iron-related indices, and circulating calcium, may remain normal or exhibit paradoxical elevations despite severe undernutrition. Accordingly, this review contributes to the evidence base underpinning the WFSBP consensus by clarifying the context-dependent interpretation of biochemical abnormalities and identifying methodological gaps that limit their clinical application. Nevertheless, the heterogeneity of the available evidence, the absence of validated diagnostic thresholds and predictive performance estimates, and the lack of intervention-focused synthesis prevent the establishment of a new clinical consensus regarding universal micronutrient screening or supplementation. Rather, our findings reinforce the need for standardized, longitudinal investigations to determine which nutritional biomarkers have reliable clinical, prognostic, or treatment-monitoring value in anorexia nervosa.

### 4.1. Phosphorus Status

The present findings suggest that phosphorus disturbances in AN are best interpreted as a dynamic marker of metabolic vulnerability during nutritional rehabilitation rather than solely as a baseline deficiency. Across the included studies, serum phosphorus was often normal at admission, whereas hypophosphatemia became more frequent after refeeding began. This pattern is biologically plausible because serum phosphate represents only a small extracellular fraction of total-body phosphorus, and intracellular depletion may remain clinically silent during prolonged undernutrition [16,168,169].

Phosphate is required for ATP generation, phosphorylation reactions, membrane phospholipids, acid–base buffering, and erythrocyte 2,3-diphosphoglycerate. During starvation, insulin concentrations and anabolic flux fall and metabolism shifts toward endogenous fat and protein stores; carbohydrate reintroduction then increases insulin secretion, cellular glucose uptake, and the synthesis of glycogen, fat, and protein. These anabolic processes consume phosphate and simultaneously promote intracellular shifts in phosphate, potassium, and magnesium, creating the characteristic biochemical pattern of refeeding syndrome [16,168,169].

This mechanism explains why serial phosphate measurements during early refeeding are more informative than a single admission value. During catabolism, extracellular phosphate can be temporarily preserved despite reduced total-body stores, whereas the sudden increase in anabolic demand after calorie delivery may expose that depletion [168,169]. Thus, a normal baseline concentration followed by a rapid decline is physiologically coherent rather than contradictory.

Clinically important hypophosphatemia can impair skeletal, respiratory, and myocardial function and can contribute to weakness, rhabdomyolysis, arrhythmias, seizures, altered consciousness, and respiratory failure. Reduced erythrocyte 2,3-diphosphoglycerate can also increase hemoglobin oxygen affinity and thereby compromise tissue oxygen delivery [168]. For this reason, phosphate decline during refeeding should be treated as a potentially systemic complication rather than an isolated laboratory abnormality [16,169].

Low BMI or a low percentage of expected body weight may identify greater risk, but weight alone is not sufficient for risk stratification. The likelihood and severity of refeeding-related electrolyte shifts also depend on the duration and recent intensity of restriction, recent weight loss, the carbohydrate and energy load delivered, pre-existing electrolyte depletion, comorbidity, and the speed of nutritional escalation [16,169]. Consequently, relatively preserved body weight or normal admission phosphate does not eliminate refeeding risk.

### 4.2. Iron Status

The iron findings argue against treating anemia in AN as synonymous with iron deficiency. In the reviewed literature, serum iron was often preserved, whereas ferritin, transferrin, total iron-binding capacity (TIBC), and hepcidin showed more complex patterns. This is consistent with the broader biology of iron biomarkers: ferritin reflects storage iron but is also influenced by inflammation and cellular stress, whereas transferrin/TIBC and circulating iron are affected by hepatic protein synthesis, nutritional state, and iron trafficking [28,29,170].

Systemic iron availability is controlled principally by the hepcidin–ferroportin axis. Hepcidin reduces intestinal iron absorption and limits iron export from macrophages and hepatocytes by inducing ferroportin internalization; its expression is shaped by iron stores, inflammation, hypoxia, and erythropoietic demand [171]. Starvation and recovery can alter several of these regulatory inputs at the same time, so serum iron concentration alone is insufficient to distinguish iron deficiency from redistribution or altered utilization [170,171].

Transferrin and TIBC also require cautious interpretation. Classical absolute iron deficiency is usually characterized by depleted storage iron and increased iron-binding capacity, whereas inflammatory or protein-energy states may lower transferrin and alter ferritin independently of total-body iron [28,29,170]. A low TIBC in a severely malnourished patient can therefore reflect reduced hepatic protein synthesis rather than iron sufficiency or iron overload.

The hematological phenotype of severe starvation can additionally reflect impaired marrow function. Gelatinous transformation and hypocellularity of bone marrow are recognized in severe nutritional and catabolic states and can produce anemia, leukopenia, thrombocytopenia, or pancytopenia without requiring a single micronutrient deficiency as the proximate cause [172]. This supports interpreting iron indices together with the complete blood count, inflammatory markers, liver tests, and the clinical stage of starvation or refeeding rather than assuming that iron replacement alone will normalize starvation-related cytopenias.

### 4.3. Selenium Status

Selenium emerged as one of the more recurrent trace-element abnormalities in the present review, particularly in severe or hospitalized populations. The clinical meaning of a low circulating selenium value nevertheless depends on the marker used, habitual intake, geography and food sources, protein status, and inflammatory state [173,174]. It is therefore more appropriate to view low selenium as a potential marker of reduced antioxidant and metabolic reserve than as an isolated explanation for the multisystem manifestations of AN.

Selenium is incorporated into selenoproteins including glutathione peroxidases, thioredoxin reductases, iodothyronine deiodinases, and selenoprotein P. Through these proteins, selenium contributes to redox regulation, thyroid-hormone metabolism, immune function, and cellular protection from oxidative injury [18,173]. Severe dietary restriction could compromise these systems, particularly when low selenium coexists with protein-energy malnutrition and other antioxidant deficiencies.

Marked selenium deficiency has been associated outside AN with myopathy and cardiomyopathic phenotypes, illustrating why selenium status may become clinically relevant in patients who already have reduced muscle mass or limited cardiovascular reserve [173]. However, these complications should not be inferred from a modestly reduced plasma selenium concentration alone.

The deiodinase role is especially relevant to starvation physiology because selenium-dependent enzymes participate in activation and inactivation of thyroid hormones [18,173]. Low selenium could therefore modify thyroid-hormone handling, but it should not be treated as a complete explanation for the adaptive low-triiodothyronine state that accompanies prolonged undernutrition.

Selenium has also been investigated in relation to neuropsychological and immune outcomes, but these associations are susceptible to confounding by overall disease burden and nutritional status [173]. In AN, fatigue, weakness, mood symptoms, or immune abnormalities are therefore unlikely to be selenium-specific and should be interpreted as potentially multifactorial.

Biomarker choice is a major limitation. Plasma or serum selenium, whole-blood selenium, selenoprotein P, and glutathione peroxidase activity do not provide interchangeable information, and their responsiveness varies with baseline status and intake [174]. Future studies in AN would be strengthened by reporting the biological matrix, assay method, inflammatory state, albumin or protein status, supplement exposure, and treatment stage alongside selenium concentrations.

Clinically, the strongest rationale for targeted selenium assessment is in severe or prolonged malnutrition, highly restricted diets, unexplained muscle weakness, cardiomyopathic features, or multiple concurrent deficiencies. Routine high-dose supplementation without biochemical or dietary justification is not supported by the heterogeneity of the present evidence and is additionally constrained by the narrow margin between selenium adequacy and excess [173].

### 4.4. Magnesium Status

Magnesium was often preserved at baseline in the reviewed cohorts, while clinically important abnormalities were more concentrated around severe malnutrition, purging, medication misuse, renal losses, or refeeding. This pattern is compatible with magnesium physiology because less than 1% of total-body magnesium is present in the extracellular fluid and a normal serum value does not necessarily demonstrate normal intracellular or skeletal stores [175].

During refeeding, insulin-driven cellular uptake and renewed ATP-dependent metabolism can increase intracellular magnesium requirements at the same time that pre-existing depletion is being unmasked [169,175]. This helps explain why magnesium may decline after nutritional rehabilitation begins even if the admission concentration was within the reference interval.

Magnesium participates in hundreds of enzymatic reactions, stabilizes ATP, influences membrane excitability, and interacts closely with potassium and calcium homeostasis [13,175]. Clinically, magnesium depletion can promote renal potassium wasting and make hypokalemia difficult to correct; it can also impair parathyroid-hormone secretion or action and contribute to hypocalcemia [13,175]. Neuromuscular symptoms and ventricular arrhythmias become more concerning when hypomagnesemia coexists with hypokalemia or other refeeding abnormalities [11,12,13].

Accordingly, magnesium should be interpreted dynamically and in relation to potassium, calcium, phosphate, renal function, gastrointestinal losses, and refeeding stage. A single normal serum magnesium value should not be considered proof of complete magnesium repletion in a severely malnourished patient [175].

### 4.5. Potassium Status

Potassium abnormalities in AN appear strongly dependent on clinical phenotype. Restricting-type patients may have normal serum potassium despite undernutrition, whereas vomiting, laxative or diuretic misuse, dehydration, renal dysfunction, and refeeding substantially increase the risk of hypokalemia [14,176,177]. This makes potassium more a marker of fluid, renal, gastrointestinal, and behavioral physiology than a simple measure of dietary potassium intake.

Most body potassium is intracellular, so serum potassium can remain within the reference range despite a reduced total-body potassium pool [14]. Renal conservation during chronic low intake can further preserve the extracellular concentration until an additional stressor—such as vomiting, diarrhea, medication misuse, or insulin-mediated intracellular uptake—reveals the deficit [14,169].

For sodium, a concentration within reference limits does not establish normal total-body sodium: hemoconcentration, water loading, dehydration or intravenous fluid can conceal or produce clinically important changes. The 14-patient severe-AN study by Caregaro et al. [41] assessed fluid and sodium balance across admission, fluid administration and discharge, illustrating that dynamic fluid status is essential to interpret this marker.

Repeated vomiting removes hydrogen and chloride and causes extracellular-volume contraction. The resulting activation of the renin–angiotensin–aldosterone system promotes renal potassium and hydrogen loss and helps maintain a hypochloremic metabolic alkalosis; the same physiology contributes to the edema-prone pseudo-Bartter state after abrupt cessation of purging [176,177,178]. Laxative-associated diarrhea, by contrast, may produce direct gastrointestinal potassium loss and a different acid–base pattern [177].

Refeeding adds a second mechanism because insulin promotes cellular potassium uptake together with phosphate and magnesium [169]. In a patient with already reduced stores, the resulting fall in serum potassium can contribute to weakness and cardiac electrical instability [12,169].

Because hypokalemia can prolong repolarization and predispose to ventricular arrhythmias, its significance increases when it coexists with bradycardia, hypomagnesemia, dehydration, or other medical complications of AN [11,12,13]. Interpretation should therefore integrate magnesium, phosphate, bicarbonate, chloride, renal function, volume status, purging history, and the electrocardiogram rather than treating potassium as an isolated nutritional marker [14,176,177,178].

### 4.6. Sodium Status

Sodium was also heterogeneous across the reviewed studies. Hyponatremia in AN is better conceptualized as a disorder of water balance and effective circulating volume than as evidence of insufficient sodium intake alone. Purging, hypovolemia, excess free-water intake, reduced solute intake, impaired renal water excretion, and medical instability can all contribute [176,179].

Serum sodium reflects the relationship between body water and exchangeable sodium plus potassium. Consequently, hyponatremia usually indicates relative water excess rather than simple sodium depletion [179]. Very low dietary protein and solute intake can reduce renal free-water excretory capacity, while deliberate water loading can further lower serum sodium in susceptible patients [176,179].

Volume depletion from vomiting, diarrhea, or diuretics can also provoke non-osmotic vasopressin release, causing the kidney to retain water even when serum osmolality is low [179]. This mechanism explains why a patient can simultaneously be clinically volume depleted and biochemically hyponatremic.

Chronic purging additionally activates the renin–angiotensin–aldosterone system. When purging stops, persistent aldosterone activity can transiently promote sodium and water retention and edema, a pattern described as pseudo-Bartter syndrome [176]. Refeeding itself can further expand extracellular volume through changes in insulin and renal sodium handling [16,168].

Severe or rapidly developing hyponatremia can cause cerebral edema, confusion, seizures, and coma, whereas overly rapid correction of chronic hyponatremia can cause osmotic demyelination [179]. Evaluation should therefore include volume status, serum and urine osmolality, urine sodium, renal function, recent fluid intake, purging behaviors, and the timing of nutritional rehabilitation.

### 4.7. Calcium Status

Calcium illustrates why a normal circulating concentration cannot be equated with normal mineral balance. Serum calcium was commonly preserved in the reviewed studies, yet AN is strongly associated with impaired bone health. Calcium concentration is tightly regulated through coordinated actions of the intestine, kidney, parathyroid glands, vitamin D, and skeleton; these homeostatic systems can maintain serum calcium within a narrow range even when dietary calcium intake or skeletal balance is unfavorable [180].

When calcium intake or intestinal absorption falls, parathyroid hormone and vitamin D-dependent mechanisms increase renal conservation and can mobilize mineral from bone to defend extracellular calcium [180]. Thus, normal serum calcium is an insensitive marker of long-term calcium sufficiency and should not be used to exclude negative calcium balance.

Bone loss in AN is multifactorial and involves low body weight, reduced mechanical loading, hypogonadism and amenorrhea, reduced IGF-1, altered cortisol and other endocrine signals, and suppression of bone formation [9,181]. Vitamin D insufficiency or low calcium intake may add to this burden, but neither is sufficient to explain the skeletal phenotype on its own [25,181].

Clinical assessment of bone risk should therefore integrate weight trajectory, menstrual or gonadal status, vitamin D, calcium intake, fracture history, and bone-density measures when indicated. Weight restoration and endocrine recovery remain central to skeletal improvement; calcium and vitamin D are supportive components rather than substitutes for nutritional rehabilitation [181].

### 4.8. Zinc Status

Zinc findings were among the most variable in the present review: marked deficiency was common in some severely malnourished cohorts, whereas other studies reported normal or even higher circulating values. Such heterogeneity is expected because plasma zinc is an imperfect biomarker and is affected by fasting status, recent meals, time of day, inflammation, hormones, albumin, and redistribution between plasma and tissues [182,183]. A normal plasma zinc concentration therefore does not definitively exclude insufficient zinc availability.

Zinc is required as a catalytic, structural, or regulatory component of numerous proteins and contributes to DNA and protein synthesis, cellular proliferation, epithelial integrity, wound healing, immunity, and antioxidant defense [19,183]. These functions become particularly relevant during nutritional rehabilitation, when tissue synthesis and repair accelerate.

Zinc also has recognized links to gustatory function, and deficiency can be accompanied by altered taste, reduced appetite, skin lesions, alopecia, impaired healing, and immune dysfunction [19,184]. These features could plausibly amplify nutritional difficulties in selected patients, but they should not be interpreted as evidence that zinc deficiency is a primary cause of AN.

Dietary bioavailability is important because zinc absorption is lower from phytate-rich diets and generally higher from animal-source foods; therefore, food selection can matter independently of total zinc intake [183]. Functional status may also diverge from plasma concentration, reinforcing the value of combining biochemical results with diet history and clinical signs [182,183].

During weight restoration, increased requirements for protein synthesis, immune recovery, skin and mucosal repair, and bone remodeling may make marginal zinc status more clinically visible. Future studies should therefore standardize fasting status, sampling time, inflammation markers, albumin, supplement exposure, and treatment stage when comparing zinc concentrations [182,183].

### 4.9. Chloride Status

Chloride was less frequently studied than sodium or potassium, but its abnormalities are clinically informative because chloride is a major extracellular anion involved in electroneutrality, extracellular-volume regulation, and acid–base physiology [185]. In AN, an isolated chloride concentration is therefore less informative than its relationship with sodium, potassium, bicarbonate, hydration, renal function, and purging behavior.

Serum chloride can remain normal despite changes in total-body chloride because concentration is influenced by water balance and renal handling [185]. Conversely, hypochloremia can signal substantial gastrointestinal or renal chloride loss rather than a primary dietary chloride deficiency.

Repeated vomiting causes loss of hydrochloric acid and chloride, generating metabolic alkalosis and extracellular-volume contraction. Volume depletion, hypochloremia, potassium deficiency, and aldosterone activity then impair renal bicarbonate excretion and help maintain the alkalosis [178]. The combination of low chloride, low potassium, and high bicarbonate is therefore particularly suggestive of vomiting or diuretic-related physiology [176,178].

Chloride should also be interpreted with sodium and osmolality when water loading or dilution is suspected. Although chloride is not usually the defining electrolyte of refeeding syndrome, changes in fluid administration, extracellular volume, and renal sodium-chloride handling can alter its concentration during medical stabilization [16,185].

### 4.10. Copper Status

Copper findings were heterogeneous, with some studies suggesting substantial deficiency and others showing preserved values. Interpretation requires attention to ceruloplasmin, because much circulating copper is protein-bound and serum copper can vary with hepatic function, inflammation, estrogen exposure, and nutritional status [17]. Thus, a serum copper value alone does not fully characterize cellular copper availability.

Copper is required for enzymes involved in mitochondrial energy metabolism, antioxidant defense, catecholamine synthesis, connective-tissue cross-linking, and iron handling [17]. Clinically significant copper deficiency can cause anemia and neutropenia and, when prolonged, can produce neurological dysfunction including sensory ataxia and myelopathy [17]. These manifestations provide a mechanistic reason to consider copper in otherwise unexplained cytopenias or neurological findings during severe malnutrition.

Copper also contributes to redox defense through copper/zinc superoxide dismutase, while ceruloplasmin participates in iron mobilization and oxidation. Consequently, copper deficiency can mimic or compound iron-related hematological abnormalities rather than producing a completely separate phenotype [17].

Because ceruloplasmin is an acute-phase protein and because high zinc exposure can interfere with copper absorption, copper should be assessed in relation to inflammation, liver function, supplement use, zinc exposure, blood counts, and neurological symptoms [17]. Routine copper supplementation is not justified by a normal or low-normal result in isolation.

### 4.11. Manganese Status

Manganese is the least characterized trace element in this review, so any interpretation should remain cautious. The available AN-specific evidence is too limited to define a reproducible deficiency or excess pattern, and serum or whole-blood manganese concentrations do not necessarily provide a complete measure of tissue status.

Biologically, manganese is an essential cofactor for enzymes involved in antioxidant defense, amino-acid and carbohydrate metabolism, gluconeogenesis, nitrogen handling, and matrix synthesis. Manganese-dependent enzymes include mitochondrial superoxide dismutase, arginase, pyruvate carboxylase, and several glycosyltransferases [186].

These functions provide plausible links to mitochondrial redox balance and skeletal or connective-tissue biology, but they should not be extrapolated into AN-specific clinical effects without direct evidence [186]. At present, manganese is best regarded as a research gap rather than a routinely actionable biomarker in AN.

Excess manganese is also neurotoxic and can accumulate in basal-ganglia circuitry, producing a parkinsonian syndrome after sufficiently high or prolonged exposure [186]. This narrow rationale for avoiding unnecessary high-dose supplementation is especially relevant when evidence of deficiency is absent.

### 4.12. Vitamin A Status

Vitamin A findings did not follow a simple deficiency model. Circulating retinol, carotenoids, and retinyl esters may move in different directions because they reflect different aspects of intake, hepatic storage, transport, and metabolism. Serum retinol is under substantial homeostatic control and can remain relatively stable until liver stores are markedly altered, while inflammation and changes in retinol-binding protein can lower circulating retinol independently of total-body stores [187].

Most vitamin A is stored in the liver, and circulating retinol is transported primarily in association with retinol-binding protein. For this reason, a normal serum retinol value cannot be assumed to prove optimal stores, while a low value must be interpreted in relation to inflammation, protein status, and hepatic function [187].

The recurrent hypercarotenemia reported in AN is compatible with the fact that carotenoid concentrations reflect both diet composition and lipid transport rather than vitamin A status alone. High carotenoid intake from vegetables can coexist with low total energy or fat intake, and conversion of provitamin A carotenoids to retinol is regulated rather than automatic [187].

Retinoic acid regulates gene transcription involved in epithelial differentiation, immune biology, growth, and bone metabolism, while preformed vitamin A in excess can itself become toxic [187]. This bidirectional risk argues against indiscriminate vitamin A supplementation in patients whose biochemical profile is not clearly deficient.

Clinically significant vitamin A deficiency can impair epithelial and mucosal integrity, immunity, vision, and tissue repair, whereas chronic excess can contribute to hepatic abnormalities, alopecia, mucocutaneous symptoms, and skeletal effects [187]. Dietary history, supplement use, liver function, and appropriate biochemical assessment should therefore guide treatment decisions.

### 4.13. Vitamin D Status

Vitamin D insufficiency or deficiency was relatively common in several included cohorts, especially in severe or hospitalized AN, but it was not universal. Variation across studies is expected because 25-hydroxyvitamin D is influenced by season, sunlight exposure, latitude, age, supplementation, dietary intake, and treatment stage in addition to body weight [25]. Vitamin D status should therefore not be inferred from BMI alone.

Vitamin D promotes intestinal calcium and phosphate absorption and interacts with parathyroid hormone to maintain mineral homeostasis [25,180]. When vitamin D is insufficient, serum calcium may remain normal because compensatory endocrine mechanisms increase renal calcium conservation and, when necessary, skeletal mineral mobilization [180]. This helps explain why low vitamin D and normal serum calcium can coexist.

In AN, low vitamin D is only one component of a much broader osteometabolic disorder. Low body weight, reduced mechanical loading, hypogonadism, amenorrhea, low IGF-1, altered cortisol signaling, and impaired bone formation all contribute to reduced bone mass [9,181]. Consequently, correcting vitamin D alone cannot be expected to reverse anorexia-related bone loss [181].

Vitamin D should be interpreted together with calcium intake, phosphate, parathyroid hormone, bone turnover markers, menstrual or gonadal status, weight trajectory, season, sunlight exposure, and supplementation. Its major actionable role in this context is as a modifiable contributor to calcium–bone physiology within a broader strategy centered on nutritional and endocrine recovery [25,181].

### 4.14. Vitamin E Status

Vitamin E status was generally preserved in the present review, although low intake or abnormal tocopherol fractions occurred in selected patients. This apparent preservation must be interpreted carefully because vitamin E is lipid-soluble and plasma α-tocopherol is transported in lipoproteins; its concentration therefore depends partly on circulating lipid concentrations and lipoprotein metabolism [188].

α-tocopherol is a major lipid-phase antioxidant that protects polyunsaturated membrane lipids from peroxidation and functions within a wider antioxidant network involving vitamin C, glutathione, and selenium-dependent systems [189]. In severe undernutrition, alterations in fat intake, lipid transport, and antioxidant availability could therefore change the biological meaning of an apparently normal absolute plasma concentration.

Because plasma α-tocopherol rises and falls with lipid carriers, lipid-adjusted indices can be more informative than an uncorrected concentration when cholesterol or triglycerides are abnormal [188]. This is particularly relevant in AN, where lipid profiles can change substantially during starvation and refeeding.

The body also preferentially retains and traffics α-tocopherol through hepatic α-tocopherol transfer protein, while other tocopherol forms are metabolized differently [189]. Consequently, one tocopherol fraction cannot be assumed to represent the entire vitamin E system.

Severe vitamin E deficiency is uncommon but can cause peripheral neuropathy, ataxia, muscle dysfunction, retinal disease, and hemolytic phenomena, especially when fat absorption or transport is impaired [188,189]. Targeted assessment is therefore most relevant in prolonged severe malnutrition, malabsorptive states, neurological symptoms, or multiple concurrent antioxidant deficiencies.

### 4.15. Vitamin K Status

Vitamin K abnormalities in the present review appeared to involve selected subgroups rather than the whole AN population. Evidence based on undercarboxylated osteocalcin is particularly interesting because this marker reflects vitamin K-dependent carboxylation in bone rather than merely a circulating vitamin concentration [20].

Vitamin K is required for γ-carboxylation of osteocalcin and other vitamin K-dependent proteins, enabling calcium binding and contributing to bone-matrix biology [20]. In AN, any impairment in vitamin K function would occur on top of established skeletal stressors such as low body weight, hypogonadism, low IGF-1, and impaired bone formation [181].

As a fat-soluble vitamin, vitamin K status can be influenced by dietary pattern, fat absorption, liver function, and medications that alter vitamin K metabolism. These variables should be considered before attributing an abnormal functional marker to deficient intake alone [20].

Elevated undercarboxylated osteocalcin indicates reduced vitamin K-dependent carboxylation but does not identify whether the underlying cause is low intake, absorption, hepatic handling, or altered bone turnover [20]. Vitamin K findings should therefore be interpreted within the broader assessment of skeletal and nutritional status.

Supplementation can improve biochemical markers of vitamin K status, but randomized evidence on bone mineral density and fracture prevention is heterogeneous and does not justify treating vitamin K as a stand-alone therapy for AN-related bone disease [190]. Weight restoration, endocrine recovery, adequate energy and protein, and sufficient calcium and vitamin D remain the central components of skeletal rehabilitation [181].

### 4.16. Vitamin B1 Status

Vitamin B1 (thiamine) status in AN appears heterogeneous rather than uniformly deficient. The clinically important issue is that thiamine requirement rises when carbohydrate metabolism is reactivated, so a marginal pre-refeeding state can become consequential during nutritional rehabilitation [169,191]. This is why refeeding guidance emphasizes thiamine assessment or supplementation in patients at risk even when a baseline circulating value is not clearly low [169].

Thiamine diphosphate is an essential cofactor for enzymes involved in oxidative carbohydrate metabolism and the pentose-phosphate pathway, including pyruvate dehydrogenase, α-ketoglutarate dehydrogenase, and transketolase [23,191]. During starvation these pathways are relatively downregulated; after carbohydrate delivery they are rapidly re-engaged, increasing thiamine-dependent metabolic flux [168,191].

Functional assessment is challenging because no single biomarker perfectly captures tissue thiamine sufficiency. Whole-blood thiamine diphosphate reflects circulating status, whereas erythrocyte transketolase activity provides a functional measure of thiamine-dependent enzyme activity [191]. This helps explain why apparently normal blood measurements do not always eliminate clinical concern in a high-risk refeeding context.

Severe thiamine deficiency can cause Wernicke encephalopathy, peripheral neuropathy, cardiac dysfunction, and lactic acidosis; the neurological presentation may be incomplete and can overlap with nonspecific manifestations of starvation [191]. Clinical risk assessment should therefore incorporate the duration of restriction, recent vomiting, prior supplementation, neurological findings, and the intensity of planned refeeding [169,191].

### 4.17. Vitamin B2 Status

Vitamin B2 findings suggest that functional riboflavin metabolism may be more informative than a single circulating concentration. Riboflavin is converted to flavin mononucleotide (FMN) and flavin adenine dinucleotide (FAD), coenzymes central to redox reactions, mitochondrial energy production, fatty-acid oxidation, and the metabolism of other vitamins [192].

Because many flavoproteins are involved in oxidative metabolism, marginal riboflavin status could theoretically amplify energetic and redox stress during severe undernutrition or recovery, although AN-specific clinical consequences remain insufficiently defined [192].

The erythrocyte glutathione reductase activation coefficient (EGRac) is a validated functional biomarker of riboflavin status because glutathione reductase is FAD-dependent. Supplementation studies show that EGRac responds to changes in riboflavin intake and can identify functional insufficiency that may not be evident from a simple circulating vitamin concentration [193].

Riboflavin also intersects with vitamin B6, folate, and iron metabolism [192]. Future studies in AN should therefore prioritize standardized functional measures such as EGRac, report supplement exposure, and interpret riboflavin in relation to the broader B-vitamin and hematological context rather than relying on one plasma value [192,193].

### 4.18. Vitamin B6 Status

Vitamin B6 status was likewise heterogeneous in the included literature, and this may partly reflect the difference between direct and functional biomarkers. Plasma pyridoxal-5′-phosphate (PLP) is the most widely used direct marker, whereas erythrocyte transaminase activity and metabolite ratios provide information about PLP-dependent function [22,194].

PLP serves as a cofactor for numerous reactions in amino-acid metabolism, transamination, glycogen metabolism, gluconeogenesis, neurotransmitter synthesis, one-carbon metabolism, and transsulfuration [22,194]. Severe malnutrition can alter protein intake, albumin, inflammation, renal function, and tissue distribution, all of which can complicate interpretation of plasma PLP [194].

Vitamin B6 is required for enzymatic steps involved in synthesis or metabolism of neurotransmitters including serotonin, dopamine, GABA, and histamine [22]. Functional insufficiency could therefore interact with neuropsychological symptoms, but current evidence does not support attributing the core psychopathology of AN to vitamin B6 status.

PLP also contributes to homocysteine transsulfuration and interacts metabolically with folate and vitamin B12 [194]. Because inflammation, albumin, renal function, supplementation, and other biochemical variables can alter B6 biomarkers, future studies should combine PLP with dietary exposure and, where feasible, functional metabolic indicators [194].

### 4.19. Vitamin B9 Status

Folate status in AN appears heterogeneous rather than uniformly deficient. The distinction between serum or plasma folate and red-blood-cell folate is important: serum folate is more responsive to recent intake, whereas erythrocyte folate integrates exposure over the lifespan of circulating red cells and is therefore more reflective of longer-term status [195].

Folate is required for one-carbon transfer reactions, nucleotide synthesis, and normal erythropoiesis. Deficiency can cause ineffective erythropoiesis, macrocytosis, and megaloblastic anemia [24,195]. However, cytopenias in severe AN can also result from starvation-related marrow suppression and gelatinous transformation, so abnormal blood counts should not automatically be assigned to folate deficiency [172].

During refeeding, renewed cell proliferation and tissue synthesis may increase demand for one-carbon metabolism, but the magnitude of this effect in AN has not been well quantified. Folate-dependent remethylation pathways also interact with vitamin B12 and vitamin B6, while homocysteine is additionally affected by renal function and other metabolic factors [24,194,195].

A normal serum folate concentration may reflect recent food intake or supplementation and does not provide exactly the same information as erythrocyte folate [195]. Assessment should therefore consider diet, supplement exposure, serum and red-cell folate where appropriate, vitamin B12, homocysteine, blood-count indices, and the stage of nutritional rehabilitation.

### 4.20. Vitamin B12 Status

Overt vitamin B12 deficiency was uncommon in the present synthesis, and several studies reported normal or elevated circulating concentrations. Total serum B12 is nevertheless an imperfect proxy for intracellular B12 function because it represents vitamin bound to transport proteins and can be influenced by supplementation, hepatic release, clearance, and binding-protein abnormalities [196,197]. Thus, an elevated value should not automatically be interpreted as superior nutritional status.

Vitamin B12 is required for methionine synthase and methylmalonyl-CoA mutase activity and thereby supports one-carbon metabolism, DNA synthesis, erythropoiesis, and neurological function [24,196]. True deficiency can cause megaloblastic anemia, neuropathy, gait disturbance, paresthesias, and cognitive or neuropsychiatric symptoms; because hepatic stores are relatively large, deficiency may develop slowly despite reduced intake [196].

When total B12 is discordant with the clinical picture, functional biomarkers can improve interpretation. Holotranscobalamin reflects the fraction available for cellular uptake, whereas methylmalonic acid and homocysteine accumulate when B12-dependent metabolism is impaired, although renal function and other factors also affect these markers [196]. Elevated total B12 can occur through increased binding proteins, hepatic release, or impaired clearance and therefore warrants contextual interpretation rather than reassurance by itself [197].

Anemia or macrocytosis in severe AN can also reflect folate deficiency, copper deficiency, inflammation, protein-energy malnutrition, or starvation-related marrow pathology rather than B12 deficiency alone [17,172]. B12 should therefore be interpreted together with the complete blood count, folate, liver and renal function, methylmalonic acid or homocysteine when indicated, and neurological findings [196,197].

### 4.21. Vitamin C Status

Vitamin C status was highly variable in the present review. Group-level concentrations were often preserved, whereas clinically important deficiency or scurvy was concentrated in patients with severe, prolonged, or highly selective restriction. This variability is plausible because plasma vitamin C is sensitive to recent intake and may not reflect tissue stores as well as leukocyte measurements [21,198].

Vitamin C is required for collagen hydroxylation, connective-tissue integrity, wound healing, antioxidant defense, and multiple enzymatic reactions, and it also contributes to immune-cell function [21]. Deficiency can produce fatigue, perifollicular petechiae, purpura, ecchymoses, gingival bleeding, musculoskeletal pain, and impaired wound healing—the clinical syndrome of scurvy [21].

Vitamin C also helps regenerate oxidized vitamin E and accumulates in leukocytes, linking it to antioxidant and immune defense [21]. These functions provide biological relevance during starvation and refeeding, but vitamin C deficiency should not be assumed to explain the broader immune or oxidative abnormalities of AN on its own.

Because plasma ascorbate can change rapidly with recent intake or supplementation, biochemical testing is most informative when interpreted with dietary history and physical examination [198]. When supplementation has already begun, a normal plasma result may be difficult to interpret; clinical signs and the timing of sampling should therefore be documented carefully [21,198].

### 4.22. Methodological Strengths and Limitations

The former low/normal/high direction counts also risked creating an impression of vote counting despite heterogeneous sample sizes and multiple non-independent outcomes per publication. Removing these counts reduces this risk but does not eliminate selection bias, lack of formal appraisal, non-independent cohorts or matrix/assay heterogeneity. The streamlined Table 1 is a structured qualitative summary, and the full evidence table has been moved to Supplementary Table S1 to retain traceability.

A major strength of the revised review is the explicit separation of evidence by study design, AN specificity, biological matrix, and clinical timing. The revised Methods also provide database-specific reconstructed search examples, explicitly distinguished from the unarchived original searches, a transparent PCC-based eligibility framework, a corrected study-selection flow, and a PRISMA-ScR reporting map. This structure reduces the risk that isolated case reports, mixed eating-disorder samples, or refeeding observations are interpreted as equivalent to AN-specific comparative or cohort evidence.

Crucially, the database-specific strings in Appendix A were reconstructed retrospectively because the original platform search histories were unavailable. These examples show the concepts intended for searching but do not preserve the exact executed queries, filters, dates of individual search runs, or original retrieved-record sets. Accordingly, the precise electronic search process cannot be independently reproduced or audited, and incomplete retrieval remains a plausible source of selection bias. The phrase “reconstructed search examples” is therefore used deliberately instead of “reproducible original search strategies”.

Clinical implications are limited to interpreting measured biomarkers in relation to baseline illness, purging or medical instability, and active refeeding; the review supports vigilance and targeted monitoring, not a universal screening panel or any particular micronutrient supplementation regimen. No conclusions about dosing, comparative effectiveness, safety or long-term benefit of supplementation can be drawn from this scoping synthesis.

Several limitations remain. First, no protocol was prospectively registered, so the final methodological framework was formalized retrospectively. Second, only English-language full texts were included, creating a risk of language bias. Third, Google Scholar is dynamic and its relevance ranking is not fully reproducible. Fourth, the evidence base spans several decades and includes substantial heterogeneity in diagnostic criteria, AN subtype definitions, illness severity, supplementation, fasting status, assay methods, biological matrices, and laboratory reference ranges. These features preclude direct quantitative pooling and complicate comparisons across studies. Fifth, the original review records did not document independent duplicate screening or duplicate data charting; this limitation should be considered when interpreting the review process and should be addressed prospectively in any future update.

A further limitation is incomplete documentation of screening and data-charting responsibilities. Available records do not show who performed title/abstract screening, full-text decisions, and extraction for each record, whether second-reviewer checks occurred, or how disagreements were resolved. Rechecking an uncertain full text against eligibility criteria and cross-checking extracted values against source reports do not establish independent dual review. These uncertainties increase the possibility of undetected selection or extraction errors and cannot be corrected by retrospective descriptions alone.

The addition of 27 studies by backward reference-list screening (19.9% of all 136 included studies) also suggests that the electronic searches may have lacked sensitivity for part of this literature. Although older publication dates, incomplete indexing, or nutrient-specific terminology are plausible explanations, the available records do not identify the 27 studies as a separate auditable group. We therefore cannot determine which explanation predominated or whether these sources were concentrated in particular nutrient domains. Prospective archiving of complete platform histories and an item-level source-of-identification field would help address this issue in an update.

In addition, some sources contained mixed eating-disorder populations or atypical AN and were therefore treated as contextual rather than direct AN evidence; residual indirectness is unavoidable when older reports do not provide subgroup-level data. Case reports and case series are valuable for rare manifestations but cannot estimate prevalence. A formal risk-of-bias or certainty-of-evidence grading system was not applied because the objective was evidence mapping rather than effect estimation, although design-based interpretive weighting was used to prevent low-directness sources from driving general conclusions. Finally, circulating micronutrient concentrations do not necessarily represent tissue stores or functional sufficiency, and selective publication of unusual deficiencies may overrepresent rare abnormalities.

### 4.23. Clinical Monitoring Versus Supplementation Efficacy

The evidence mapped here supports clinical awareness and context-appropriate biochemical assessment, particularly serial evaluation of phosphate and other electrolytes during nutritional rehabilitation and individual evaluation of severe restriction, purging, volume instability or suspected deficiency [16,32,36,38,41]. These observations identify when measurements may change and which phenotypes warrant attention; they do not estimate the benefit of a laboratory surveillance schedule, establish uniform test frequencies, or demonstrate that testing every micronutrient in every patient improves outcomes. Such choices remain dependent on clinical assessment and applicable management guidelines.

Evidence for treatment must be distinguished from evidence for abnormal laboratory markers. This scoping review did not systematically evaluate controlled supplementation trials, compare vitamin/mineral formulations, establish doses or duration, or measure patient-important outcomes attributable to supplementation. A finding of low zinc, selenium, vitamin D or another biomarker is not itself proof that routine empiric supplementation improves weight restoration, symptoms, bone outcomes or survival. Replacement of established deficiencies and prevention or treatment of refeeding complications should follow existing clinical protocols and individual medical judgment, rather than be claimed as an efficacy conclusion of this review. Specific supplement effects require dedicated controlled trials or intervention-focused systematic reviews. Observational or single-arm reports involving supplementation were used here only for status/trajectory mapping.

Data Availability Statement: No new primary data were generated. A condensed qualitative nutrient-level synthesis is provided in Table 1, and the full original 136-study evidence matrix has been moved to Supplementary Table S1 (separate DOCX). Illustrative reconstructed search strings (not archived search histories) and the PRISMA-ScR reporting map remain in Appendix A and Appendix B.

## 5. Conclusions

This scoping review maps a broad and methodologically heterogeneous evidence base rather than estimating a pooled prevalence of micronutrient deficiency. Across AN-specific cohort and comparative evidence, biochemical micronutrient status was not uniformly depleted. Many circulating vitamins, minerals, trace elements, and electrolytes were preserved or inconsistently altered despite severe dietary restriction, while clinically important abnormalities clustered in particular nutrients, phenotypes, and treatment stages. Normal serum or plasma values therefore should not be interpreted automatically as evidence of adequate tissue stores or absence of nutritional risk.

The clearest stage-dependent pattern concerned phosphorus during nutritional rehabilitation: admission values were often normal, yet hypophosphatemia frequently emerged during refeeding. Potassium, sodium, and magnesium disturbances were most relevant in severe malnutrition, purging, laxative or diuretic exposure, altered water balance, or acute medical instability. Serum calcium was commonly maintained despite substantial skeletal compromise, illustrating the limits of using a tightly regulated circulating marker as a proxy for whole-body mineral status.

Among micronutrients, recurrent abnormalities were reported most consistently for selenium, zinc, and vitamin D in several clinical cohorts, while copper, folate, and fat-soluble-vitamin findings were more variable and context dependent. The direction and apparent prevalence of abnormalities differed with illness severity, AN subtype, supplementation, treatment stage, biological matrix, assay method, and reference range. Accordingly, individual measurements should be interpreted in clinical context rather than generalized from isolated studies or case reports.

Overall, anorexia nervosa should be viewed as a state of complex nutritional and metabolic dysregulation rather than universal measurable micronutrient depletion. Future studies should use standardized sampling, clearly report supplementation and refeeding status, separate clinical subtypes and illness stages, and include functional markers in addition to serum or plasma concentrations. Such work is needed to clarify which abnormalities represent true deficiency, adaptive starvation physiology, redistribution, or acute treatment-related risk.

## Figures and Tables

**Table 1:** Condensed nutrient-level synthesis by evidence type, principal biochemical pattern, and interpretive limitation. Illustrative citations refer to the main reference list. This is a qualitative scoping map, not a pooled analysis or an unweighted vote count; the complete 136-source study-level matrix is available as Supplementary Table S1.

Nutrient	Evidence Type/Biological Matrix	Main Pattern	Key Limitations
Phosphorus	AN-specific inpatient longitudinal/retrospective series; serum phosphate: [31,32,33,34]	Admission often preserved; early refeeding nadir may be low (99 female inpatients [32]).	Timing and preventive phosphate use; no harmonized nadir/threshold.
Potassium	Inpatient cohorts, subtype comparisons; serum: [35,36,37,38]	Lower with binge–purge phenotype; may decline in stabilization/refeeding.	Purging, renal losses, diuretics/laxatives and sampling stage.
Sodium	Admission cohorts; small repeated-measures fluid study (*n* = 14): [39,40,41]	Usually preserved in serum; depletion, hyponatremia or fluid dysregulation in selected unstable patients.	Hydration, water loading, infusion/edema; concentration ≠ total sodium.
Magnesium	Baseline plasma and serial hospital serum: [31,36,38]	Often normal pre-treatment; new deficits possible during refeeding.	Intracellular depletion not assessed; laxative exposure and renal handling.
Calcium	Baseline plasma/serum and skeletal [31,32,33,34] studies: [31,42,43]	Serum often preserved despite poor bone health.	Serum calcium is not a proxy for bone stores; endocrine confounding.
Iron	AN hematological outpatient cohort *n* = 318 and inpatient reports: [44,45,46]	Anemia may coexist with preserved iron and high ferritin/low transferrin.	Inflammation, starvation, selection and distinct iron biomarkers.
Zinc	Severe AN cohorts and mixed AN/ARFID comparative groups: [47,48,49]	Low circulating zinc in some severely malnourished/subtype-defined groups.	Mixed diagnoses, inflammation, binding proteins, serum vs. plasma.
Selenium	AN baseline cohort *n* = 153; severe inpatient *n* = 374: [31,48,50]	Low plasma/serum selenium in clinically important subgroups.	Observational severity associations; thresholds, diet and setting.
Copper	Severe AN inpatient and baseline cohorts: [31,48,49]	Decreased circulating copper in some severe patients; not universal.	Acute-phase response, assay and hair vs. circulating measures.
Chloride	Medical admission series and individual cases: [41,51]	Hypochloremia chiefly in purging/volume-disturbed presentations.	Mostly small/selected evidence; acid–base and current losses.
Manganese	Heterogeneous eating-disorder cohort: [52]	Low circulating manganese reported in a mixed clinical sample.	Insufficient AN-specific replication, small AN strata, one main source.
Vitamin A	AN cohort *n* = 153 and biochemical studies: [31,53,54]	Retinol often maintained; some deficiency, high carotenoids.	Different analytes, diet, liver transport and assay methods.
Vitamin D	Severe AN cohorts and controlled/longitudinal samples: [48,49,55,56,57,58]	Low 25(OH)D in some severe clinical settings; not universal.	Season, supplements, thresholds, age and illness severity.
Vitamin E	Baseline AN and antioxidant studies: [31,53,59]	Circulating tocopherol often normal; compartment-specific changes.	Lipid carriage, supplement use, small heterogeneous studies.
Vitamin K	AN vs. controls (54 vs. 15), functional marker: [60]	Undercarboxylated osteocalcin elevated in some patients.	Functional surrogate, bone turnover; little direct vitamin K data.
Vitamin B1	Functional case–control (37 vs. 50), cohorts: [61,62]	Thiamine biomarker findings depend on assay and treatment phase.	Erythrocyte activity vs. whole blood; different supplement exposures.
Vitamin B2	AN vs. controls (17 vs. 17), biological compartments: [63,64]	Plasma FAD, erythrocyte riboflavin and urinary products diverged.	Very small sample and thyroid/feeding confounding.
Vitamin B6	Selected AN/mixed cohorts and functional testing: [63,65]	Plasma values may appear preserved despite altered functional markers.	Mixed diagnoses, matrix discordance, sparse AN-specific evidence.
Vitamin B9/folate	AN cohorts; serum and red-cell markers: [31,44,46,66]	Deficiency in subsets but preserved/higher results elsewhere.	Recent intake, supplement history, serum ≠ erythrocyte folate.
Vitamin B12	Severe AN cohorts and pre-treatment retrospective *n* = 71: [31,48,67]	Deficiency uncommon; serum cobalamin sometimes elevated.	Liver dysfunction; absence of functional MMA/holoTC in many studies.
Vitamin C	Severe AN cohort and rare scurvy reports: [49,68,69]	Often preserved overall, but severe deficiency in selectively restricted cases.	Case reports cannot quantify frequency; recent intake affects plasma.

## Data Availability

No new data were created or analyzed in this study. Data sharing is not applicable to this article.

## Supplementary Materials

The following supporting information can be downloaded at: https://www.mdpi.com/article/10.3390/nu18203450/s1, Supplementary Table S1. Complete study-by-study evidence matrix (136 sources). Supplement to: Beyond Body Weight: Mapping Vitamin, Mineral, and Trace-Element Status in Anorexia Nervosa—Scoping Review. The 136 evidence rows are reproduced from the submitted manuscript, with original study findings and interpretations preserved. These rows are not equivalent in design or diagnostic specificity. Refs. [31,32,33,34,35,36,37,38,39,40,41,42,43,44,45,46,47,48,49,50,51,52,53,54,55,56,57,58,59,60,61,62,63,64,65,66,67,68,69,70,71,72,73,74,75,76,77,78,79,80,81,82,83,84,85,86,87,88,89,90,91,92,93,94,95,96,97,98,99,100,101,102,103,104,105,106,107,108,109,110,111,112,113,114,115,116,117,118,119,120,121,122,123,124,125,126,127,128,129,130,131,132,133,134,135,136,137,138,139,140,141,142,143,144,145,146,147,148,149,150,151,152,153,154,155,156,157,158,159,160,161,162,163,164,165,166] correspond to the main article reference list. The table is a descriptive evidence map, not an assessment of supplementation efficacy.

## Appendix A. Reconstructed Electronic Search Examples (Not Archived Search Histories)

The searches covered database inception through 30 March 2026. No publication-year limit and no language filter were applied electronically; language eligibility was assessed at full text. The database-specific examples below were reconstructed retrospectively from the three search concept blocks described in the manuscript (AN, micronutrients, and biological status). They illustrate how these concepts could be operationalized; they are not verbatim archived search-history exports, and they cannot reproduce or audit the precise original searches, search settings, or retrieved results. Platform syntax may require minor interface-specific formatting if a database updates its search engine.

**Source**	**Illustrative Reconstructed Query/Procedure (not the Recorded Original Execution)**
PubMed/MEDLINE	(“Anorexia Nervosa”[Mesh] OR “anorexia nervosa”[tiab]) AND (micronutrient*[tiab] OR vitamin*[tiab] OR mineral*[tiab] OR “trace element*”[tiab] OR electrolyte*[tiab] OR calcium[tiab] OR magnesium[tiab] OR phosphorus[tiab] OR phosphate[tiab] OR potassium[tiab] OR sodium[tiab] OR chloride[tiab] OR iron[tiab] OR ferritin[tiab] OR transferrin[tiab] OR hepcidin[tiab] OR zinc[tiab] OR selenium[tiab] OR copper[tiab] OR manganese[tiab] OR retinol[tiab] OR carotene[tiab] OR “vitamin A”[tiab] OR thiamin*[tiab] OR “vitamin B1”[tiab] OR riboflavin[tiab] OR “vitamin B2”[tiab] OR pyridoxin*[tiab] OR “vitamin B6”[tiab] OR folate[tiab] OR “folic acid”[tiab] OR “vitamin B9”[tiab] OR cobalamin[tiab] OR “vitamin B12”[tiab] OR “ascorbic acid”[tiab] OR “vitamin C”[tiab] OR “vitamin D”[tiab] OR “25-hydroxyvitamin D”[tiab] OR calcidiol[tiab] OR tocopherol*[tiab] OR “vitamin E”[tiab] OR “vitamin K”[tiab]) AND (serum[tiab] OR plasma[tiab] OR blood[tiab] OR erythrocyte*[tiab] OR urine[tiab] OR urinary[tiab] OR hair[tiab] OR concentration*[tiab] OR level*[tiab] OR status[tiab] OR deficien*[tiab] OR insufficien*[tiab] OR biomarker*[tiab] OR hypophosphat*[tiab] OR hypokal*[tiab] OR hyponatr*[tiab] OR hypomagnes*[tiab])
Scopus	TITLE-ABS-KEY(“anorexia nervosa”) AND TITLE-ABS-KEY(micronutrient* OR vitamin* OR mineral* OR “trace element*” OR electrolyte* OR calcium OR magnesium OR phosphorus OR phosphate OR potassium OR sodium OR chloride OR iron OR ferritin OR transferrin OR hepcidin OR zinc OR selenium OR copper OR manganese OR retinol OR carotene OR “vitamin A” OR thiamin* OR “vitamin B1” OR riboflavin OR “vitamin B2” OR pyridoxin* OR “vitamin B6” OR folate OR “folic acid” OR “vitamin B9” OR cobalamin OR “vitamin B12” OR “ascorbic acid” OR “vitamin C” OR “vitamin D” OR “25-hydroxyvitamin D” OR calcidiol OR tocopherol* OR “vitamin E” OR “vitamin K”) AND TITLE-ABS-KEY(serum OR plasma OR blood OR erythrocyte* OR urine OR urinary OR hair OR concentration* OR level* OR status OR deficien* OR insufficien* OR biomarker* OR hypophosphat* OR hypokal* OR hyponatr* OR hypomagnes*)
Web of Science Core Collection	TS = (“anorexia nervosa”) AND TS = (micronutrient* OR vitamin* OR mineral* OR “trace element*” OR electrolyte* OR calcium OR magnesium OR phosphorus OR phosphate OR potassium OR sodium OR chloride OR iron OR ferritin OR transferrin OR hepcidin OR zinc OR selenium OR copper OR manganese OR retinol OR carotene OR “vitamin A” OR thiamin* OR “vitamin B1” OR riboflavin OR “vitamin B2” OR pyridoxin* OR “vitamin B6” OR folate OR “folic acid” OR “vitamin B9” OR cobalamin OR “vitamin B12” OR “ascorbic acid” OR “vitamin C” OR “vitamin D” OR “25-hydroxyvitamin D” OR calcidiol OR tocopherol* OR “vitamin E” OR “vitamin K”) AND TS = (serum OR plasma OR blood OR erythrocyte* OR urine OR urinary OR hair OR concentration* OR level* OR status OR deficien* OR insufficien* OR biomarker* OR hypophosphat* OR hypokal* OR hyponatr* OR hypomagnes*)
Google Scholar (Supplementary)	Query families: “anorexia nervosa” micronutrient serum; “anorexia nervosa” vitamin plasma deficiency; “anorexia nervosa” mineral electrolyte blood; “anorexia nervosa” zinc selenium copper iron; “anorexia nervosa” phosphorus magnesium potassium sodium calcium; “anorexia nervosa” thiamine riboflavin pyridoxine folate cobalamin; “anorexia nervosa” “vitamin A” “vitamin D” “vitamin E” “vitamin K” “vitamin C”. Google Scholar was used for supplementary discovery and citation verification rather than as the sole source for any conclusion.
Other methods	Backward reference-list screening of all eligible studies and relevant reviews; candidate primary studies were assessed using the same eligibility criteria as database-derived records.

## Appendix B. PRISMA-ScR Reporting Map

The table below maps the PRISMA-ScR checklist items to the revised manuscript. Item descriptions are abbreviated; item numbering follows Tricco et al. [199].

**Item**	**Abbreviated PRISMA-ScR Domain**	**Where Addressed in Revised Manuscript**
1	Title	Title identifies the work as a scoping review.
2	Structured summary	Structured Abstract: Introduction, Materials and Methods, Results, Conclusions.
3	Rationale	Introduction: fragmentation and heterogeneity of micronutrient evidence.
4	Objectives	Introduction and Methods 2.1: explicit mapping objectives and review question.
5	Protocol/registration	Methods 2 and 4.22: no prospective protocol registration; reported transparently.
6	Eligibility criteria	Methods 2.1–2.2: PCC framework, source types, mixed-sample rules, exclusions.
7	Information sources	Methods 2.3: databases, Google Scholar, citation searching, end date.
8	Search	Methods 2.4 and Appendix A: reconstructed examples; original search execution not reproducible from preserved records.
9	Selection of sources	Methods 2.5: two-stage screening, rechecking of uncertain records; no documented formal consensus process, exclusion reasons.
10	Data charting	Methods 2.6: structured, iterative charting and source checks; individual roles and independent verification not documented.
11	Data items	Methods 2.6–2.7: design, population, matrix, timing, thresholds, numerical and clinical variables.
12	Critical appraisal	Methods 2.8: no formal risk-of-bias score; author-defined A–D stratification and qualitative interpretive factors; no formal critical appraisal.
13	Synthesis	Methods 2.9: narrative synthesis, timing and matrix considered, descriptive direction counts defined.
14	Selection results	Results and Figure 1: database records, retrieval, exclusions, citation searching, final included total.
15	Characteristics of sources	Table 1: population/design, nutrient markers, results, interpretation; contextual sources marked †.
16	Critical appraisal within sources	Not formally performed; rationale and interpretive weighting stated in Methods 2.8.
17	Results of individual sources	Table 1 and nutrient-specific Results subsections.
18	Synthesis of results	Results subsections and Discussion, organized by nutrient and clinical stage.
19	Summary of evidence	Discussion and Conclusions.
20	Limitations	Discussion 4.22: unarchived searches, undocumented reviewer roles, 27 citation-derived studies, author-defined A–D weighting, and other limitations.
21	Conclusions	Section 5: implications and research priorities.
22	Funding	Funding statement: no external funding.
