# Supplementary material for: Beyond Body Weight: Mapping Vitamin, Mineral, and Trace-Element Status in Anorexia Nervosa—Scoping Review
Source: Nutrients. 2026 Oct 21;18(20):3450. doi: 10.3390/nu18203450 (PMC13708111; doi:10.3390/nu18203450)
Supplement: Supplementary file 1 [file nutrients-18-03450-s001.zip › nutrients-4606497-Supplementary.pdf]

Supplementary Table S1. Complete study-by-study evidence matrix (136 sources)

Supplement to: Beyond Body Weight: Mapping Vitamin, Mineral, and Trace-Element Status in Anorexia Nervosa – Scoping Review.

The 136 evidence rows are reproduced from the submitted manuscript, with original study findings and interpretations preserved. These rows are not equivalent in design or diagnostic specificity. References [31–166] correspond to the main article reference list. The table is a descriptive evidence map, not an assessment of supplementation efficacy.

Study	Population / design	Nutrients or markers assessed	Main results relevant to micronutrient status	Overall interpretation
[47]	Adults with severe eating/feeding disorders: AN-R, AN-BP, ARFID; controls without eating disorders	Serum zinc	Mean admission zinc was lower in cases than controls: 63.28 µg/dL vs 73.40 µg/dL. Low zinc occurred in 40.2% of cases vs 4.3% of controls. AN-BP had the lowest zinc levels, followed by AN-R and ARFID. Zinc was significantly higher in AN-R than AN-BP.	Zinc deficiency was frequent in severe restrictive eating disorders, especially in AN-BP, suggesting that purging behavior may contribute to lower zinc status.
[70]†	Longitudinal cohort of patients with eating disorders, including AN, BN, and other eating disorders	Plasma selenium	Median selenium was 82.9 µg/L. Low selenium was defined as ≤83 µg/L. The low-selenium group showed greater clinical severity and was associated with later suicide attempt risk.	Selenium status may reflect broader biological and clinical vulnerability in eating disorders, although the sample was not limited to AN.
[48]	Large cohort of extremely malnourished hospitalized adults with AN-R and AN-BP	Zinc, vitamin D, copper, selenium, vitamin B1, vitamin B9, vitamin B12	92.8% had at least one micronutrient deficiency. Zinc deficiency was most common, affecting 64.3%. Vitamin D deficiency occurred in 54.2%, copper deficiency in 37.1%, selenium deficiency in 20.5%, vitamin B1 deficiency in 15%, vitamin B9 deficiency in 8.9%, and vitamin B12 deficiency in 4.7%. Copper was lower in AN-R, whereas selenium and vitamin B12 were lower in AN-BP.	This large cohort indicates that severe AN is associated with multiple micronutrient deficiencies, especially zinc and vitamin D. Subtype differences were present for selected nutrients but not for all deficiencies.
[31]	Retrospective study of 153 adult female AN patients before nutritional treatment	Zinc, copper, phosphorus, magnesium, calcium, selenium, vitamin E, vitamin B12, folate, vitamin A	Mean concentrations were mostly within normal ranges. However, 43.7% had at least one trace-element deficiency and 45.7% had at least one vitamin deficiency. Selenium deficiency was most frequent among trace elements, affecting 40.6%. Vitamin A deficiency occurred in 32.8%, folate deficiency in 15.9%, zinc deficiency in 2.1%, phosphorus deficiency in 3.5%, magnesium deficiency in 3.0%, calcium deficiency in 1.3%, vitamin E deficiency in 1.5%, and vitamin B12 deficiency in 2.2%.	Although group means were often normal, individual deficiencies were common. Selenium, vitamin A, and folate appeared most vulnerable in this cohort.
[71]	Acutely ill AN, remitted AN, and healthy controls	Serum zinc, calcium, inorganic phosphate	Remitted patients had higher zinc than both acutely ill AN patients and controls. Acutely ill AN patients did not differ significantly from controls in zinc. Acutely ill patients had higher inorganic phosphate than controls and remitted patients and slightly higher calcium than controls.	This study did not support universal zinc deficiency in acute AN; instead, zinc status differed unexpectedly by recovery/remission state.

Study	Population / design	Nutrients or markers assessed	Main results relevant to micronutrient status	Overall interpretation
[72]	Childhood-onset AN	Plasma zinc, urinary zinc excretion	Zinc deficiency was common. Fasting plasma zinc correlated significantly with malnutrition severity.	Zinc depletion may be clinically relevant in childhood-onset AN and may partly track undernutrition.
[32]	Japanese female inpatients with AN and BMI <17.5 kg/m²	Serum phosphate during refeeding	Refeeding hypophosphatemia occurred in 21.2% of admissions. It appeared early during admission and resolved by Day 19. Patients who developed hypophosphatemia had lower admission BMI.	Phosphate disturbance during refeeding was common and was related to malnutrition severity.
[33]	Hospitalized adolescents and young adults with AN	Phosphorus during hospitalization	Hypophosphatemia developed in 28.7% of patients. Mean phosphorus nadir was 3.2 mg/dL overall and 2.5 mg/dL among those with hypophosphatemia.	Phosphorus decline was a frequent refeeding-related abnormality in adolescent/young adult AN, often occurring early but sometimes later.
[35]	Patients with extremely severe AN, BMI <13 kg/m²	Phosphorus, magnesium, potassium	Admission phosphorus, magnesium, and potassium did not differ significantly between hypoglycemia and non-hypoglycemia groups.	Despite extreme malnutrition, baseline electrolyte values were not consistently abnormal, indicating that normal admission values may not exclude severe nutritional risk.
[49]	354 adults with extremely severe AN admitted to a specialized nutrition/eating-disorders unit	Folate, vitamin B12, vitamin D, zinc, copper, selenium, vitamin C/scurvy lesions	Vitamin D deficiency was present in 62.9% of tested patients, zinc deficiency in 56.8%, copper deficiency in 30.1%, selenium deficiency in 21.3%, folate deficiency in 10.6%, and vitamin B12 deficiency in 4.3%. Scurvy-like cutaneous lesions with low vitamin C were reported in 1.1%.	Severe AN was associated with a high burden of micronutrient deficiencies, particularly vitamin D and zinc, with additional copper and selenium depletion.
[73]	Ten extremely undernourished AN patients undergoing refeeding	Phosphate, potassium, magnesium, sodium, calcium, chloride, bicarbonate	Baseline phosphorus averaged 3.4 mg/dL; three patients already had hypophosphatemia before treatment. Baseline potassium, magnesium, sodium, and chloride were monitored as refeeding-relevant electrolytes.	Phosphate was the key mineral abnormality, with some patients already depleted before refeeding.
[74]	Case report of a 15-year-old girl with AN and severe weight loss	Serum phosphate	Severe hypophosphatemia developed during nutritional support, with phosphate falling to 0.4 mg/dL.	The case illustrates severe refeeding-related phosphate collapse in adolescent AN.
[44]	Fourteen males hospitalized for severe AN	Phosphorus, magnesium, calcium, vitamin D, vitamin B12, folate, iron indices	Four patients, or 29%, had low phosphorus at admission. Magnesium and calcium were generally preserved. Vitamin D was low in approximately 64–65%. No vitamin B12, folate, or iron deficiency was found.	In severely malnourished males with AN, vitamin D deficiency and hypophosphatemia were prominent, whereas B12, folate, iron, calcium, and magnesium were relatively preserved.
[75]†	Hospitalized adolescents with AN or atypical AN	Phosphate, potassium, magnesium	Hypophosphatemia occurred in 41%. Hypokalemia occurred in 3%, and hypomagnesemia in 9%. Hypophosphatemia was not strongly predicted by admission weight or weight-loss measures.	Phosphate disturbance was common even in restrictive eating disorders that included atypical AN, while potassium and magnesium abnormalities were less frequent.
[45]	318 female AN patients; study focused on hematology and iron metabolism	Serum iron, ferritin, transferrin, total iron-binding capacity	Mean serum iron was 84.5 μg/dL. Low serum iron occurred in 6.2%, elevated serum iron in 1.9%. Ferritin was not low because low-ferritin patients were excluded; 33.2% had elevated ferritin. Transferrin was low in 24.2%.	Iron deficiency was not the major explanation for anemia in this cohort; iron markers were often normal or altered in ways not consistent with simple deficiency.

Study	Population / design	Nutrients or markers assessed	Main results relevant to micronutrient status	Overall interpretation
[39]	65 patients with severe AN undergoing refeeding	Phosphate, magnesium, potassium, sodium, calcium	Median lowest phosphate before treatment was 1.10 mmol/L, within normal range. Magnesium, potassium, sodium, and calcium were largely within reference ranges before treatment. Severe hyponatremia occurred in one patient before treatment.	Baseline electrolyte values were mostly preserved despite severe malnutrition, but monitoring remained clinically important during refeeding.
[76]	125 Asian adolescents with AN admitted for inpatient stabilization	Serum phosphate	Baseline phosphate was similar between lower- and higher-calorie refeeding groups. Mild hypophosphatemia occurred in 38% of the higher-calorie group and 17% of the lower-calorie group. Moderate hypophosphatemia occurred in 10% and 7%, respectively. No severe hypophosphatemia occurred.	Higher-calorie refeeding increased mild phosphate decline but did not produce severe hypophosphatemia in this monitored cohort.
[77]	Three-year follow-up of young women previously hospitalized for severe restrictive AN	Ferritin, hemoglobin, folate, retinol, tocopherol, 25-hydroxyvitamin D	Serum folate, retinol, tocopherol, and vitamin D were adequate on average. However, 20% had vitamin D below 50 nmol/L, 25% had ferritin below reference range, and 5% had low hemoglobin. Serum retinol and tocopherol were not below reference range.	After intensive treatment, biochemical vitamin status was mostly restored, but vitamin D insufficiency and low ferritin persisted in a subset.
[36]	Hospital admissions of patients with AN; restrictive and binge–purge subtypes	Potassium, phosphorus, magnesium	Mean admission potassium was 3.6 mmol/L overall. AN-BP had significantly lower potassium than AN-R: 3.2 vs 4.0 mmol/L. Mean phosphorus was 3.9 mg/dL. Albumin-corrected magnesium averaged 2.1 mg/dL, with higher values in AN-BP, possibly confounded by magnesium oxide laxative use.	Potassium disturbance was particularly associated with binge–purge subtype, while phosphorus and magnesium were less clearly depleted at admission.
[78]†	Children and adolescents with eating disorders undergoing inpatient nutritional rehabilitation	Phosphorus, potassium, magnesium	Admission potassium averaged 4.2 mmol/L, phosphorus 4.2 mg/dL, and magnesium 2.1 mg/dL. Admission hypokalemia occurred in 1.9%, hypophosphatemia in 4.2%, and hypomagnesemia in 0%.	Most patients did not present with low phosphorus, potassium, or magnesium at admission, despite clinical malnutrition.
[40]	103 extremely underweight adult female AN patients	Phosphate, sodium	Mean admission phosphate was 1.15 mmol/L. Hypophosphatemia below 0.75 mmol/L occurred in 6.8%, with one critically low value. Mean sodium was 138.7 mmol/L; pathological sodium values occurred in 6.8%, including one critical hyponatremia case.	Admission phosphate and sodium were usually preserved, but clinically important abnormalities occurred in a minority of extremely underweight patients.
[79]	46 admissions involving adolescents with AN	Serum phosphorus	Hypophosphatemia developed in 38% of admissions. All cases were mild; no moderate or severe hypophosphatemia and no clinical refeeding syndrome occurred. Lower percentage ideal body weight was associated with hypophosphatemia.	Mild phosphate decline during refeeding was common and related to severity of malnutrition.

Study	Population / design	Nutrients or markers assessed	Main results relevant to micronutrient status	Overall interpretation
[37]	Japanese patients with severe AN and very low BMI	Phosphorus, potassium	Mean admission phosphorus was 3.9 mg/dL, within or slightly above reference range. Mean potassium was 3.5 mmol/L, slightly below reference range. AN-BP had much lower potassium than AN-R: 2.8 vs 3.8 mmol/L.	Potassium depletion was prominent in binge–purge AN, while admission phosphorus was often normal despite severe malnutrition.
[34]	123 women with severe AN admitted for medical stabilization	Phosphorus, potassium, magnesium, sodium	Refeeding hypophosphatemia occurred in 33.3%. Median admission phosphorus was 3.3 mg/dL. Lower admission potassium was associated with later hypophosphatemia and remained an independent protective factor in adjusted analysis. Magnesium and sodium differed in bivariate analyses but were not independent predictors.	Normal baseline phosphorus did not exclude later refeeding hypophosphatemia. Potassium status may help identify patients at higher phosphate-related refeeding risk.
[80]	Case report of severe AN restricting type	Phosphorus, potassium	Admission phosphorus and potassium were normal, but the patient developed severe, recurrent, persistent phosphorus disturbance during nutritional rehabilitation.	The case emphasizes that normal baseline phosphate does not exclude severe refeeding-related phosphate instability.
[38]	310 adolescents and young adults with AN hospitalized for medical instability	Phosphorus, magnesium, potassium	Hypophosphatemia developed in 15.8%, hypomagnesemia in 15.2%, and hypokalemia in 20.0%. Rates did not differ significantly between lower- and higher-calorie refeeding groups. No full clinical refeeding syndrome occurred.	Electrolyte abnormalities were common during refeeding, but higher-calorie initiation did not significantly increase phosphorus, magnesium, or potassium complications under monitoring.
[81]	537 hospitalized young people with eating disorders; AN subgroup analyzed	Plasma zinc	Mean plasma zinc was 63.98 µg/dL. Zinc deficiency, defined as <55 µg/dL, occurred in 24.77%. Zinc deficiency was similar in males and females. In the AN subgroup, zinc deficiency occurred in 24.32% of males and 23.40% of females. Zinc deficiency was associated with anemia in males in the full eating-disorder sample.	Zinc deficiency affected roughly one-quarter of hospitalized young patients and was not sex-specific.
[82]†	545 adults with severe restrictive eating disorders: AN-R, AN-BP, ARFID	Phosphorus, potassium, sodium, magnesium, calcium, vitamin D	Phosphorus was normal in 84%, low in 11%, and high in 5%. Hypokalemia occurred in 39%, especially in AN-BP. Hyponatremia occurred in 25%. Magnesium was low in 2%, calcium low in 4%. Vitamin D was normal in 71%, low in 25%, and high in 4%.	Electrolyte disturbances, especially potassium and sodium abnormalities, were common; vitamin D deficiency affected about one-quarter of patients.
[83]	307 medically ill inpatients with AN or ARFID	25-hydroxyvitamin D, phosphorus	Vitamin D deficiency occurred in 7%, insufficiency in 23%, and sufficiency in 70%. Around 30% had suboptimal vitamin D. ARFID had lower mean vitamin D than AN. Hypophosphatemia occurred in approximately 35.3–35.5% during admission. No clear direct association between vitamin D and phosphorus nadir was demonstrated.	Vitamin D insufficiency/deficiency and refeeding hypophosphatemia were both common, but their relationship was not straightforward.

Study	Population / design	Nutrients or markers assessed	Main results relevant to micronutrient status	Overall interpretation
[84]	Adolescents with AN or ARFID	Serum zinc	Low zinc occurred in 50% of the total sample: 53.3% of AN and 46.2% of ARFID. Mean zinc was numerically lower in AN than ARFID, but the difference was a trend rather than statistically significant.	Zinc deficiency was very common in adolescents with restrictive eating disorders and was not fully explained by BMI or percent median body weight.
[42]	Men with AN, atypical AN, ARFID, and healthy controls; study focused mainly on bone outcomes	25-hydroxyvitamin D; calcium intake	Mean 25(OH)D was 28.3 ± 16.2 ng/mL in AN, 33.0 ± 14.0 ng/mL in ARFID, and 25.6 ± 8.5 ng/mL in atypical AN. Vitamin D deficiency, defined as <20 ng/mL, occurred in 24% of men with AN, 0% with ARFID, and 21% with atypical AN. Calcium intake was assessed in a subset.	Vitamin D deficiency was present in a clinically relevant minority of males with AN and atypical AN, although the study was not a broad micronutrient survey.
[60]	Amenorrheic women with AN-R and AN-BP compared with healthy controls	Vitamin K status using undercarboxylated osteocalcin; 25(OH)D; 1,25(OH)$_2$D; calcium; phosphorus	Vitamin K deficiency, defined by elevated ucOC, occurred in 17% of AN-R and 40% of AN-BP. Vitamin D deficiency was also common: 51% in AN-R and 72% in AN-BP. Serum calcium was normal, while phosphorus was higher in AN than controls but remained within the normal range.	The study suggests functional vitamin K deficiency and frequent vitamin D deficiency, especially in binge/purge AN, despite preserved serum calcium.
[85]	Case report of an 18-year-old female with severe AN	Selenium, vitamin B12, folate, phosphorus, calcium, potassium, sodium, chloride	Selenium was low on day 60 and normalized after supplementation. Vitamin B12 and folate were elevated rather than deficient. Phosphorus fell to 1.9 mg/dL during hospitalization, calcium remained low for much of early admission, and potassium was low at admission.	The case highlights selenium deficiency as a possible contributor to macrocytic anemia, together with refeeding-related hypophosphatemia, hypocalcemia, and hypokalemia.
[50]	153 patients with AN; selenium-focused study	Plasma selenium; vitamin B12	Selenium deficiency, defined as <80 µg/L, occurred in 53.6% of patients. Selenium-deficient patients also had significantly lower vitamin B12 concentrations than those with normal selenium.	Selenium deficiency appeared highly prevalent and was not explained simply by current BMI, suggesting that selenium may capture a specific micronutrient vulnerability in AN.
[86]†	Hospitalized male adolescents and young adults with atypical AN, compared with other restrictive eating-disorder groups	25(OH)D, zinc, magnesium, phosphorus, potassium	In males with atypical AN, vitamin D insufficiency occurred in 42.1% and deficiency in 10.5%; combined low vitamin D status occurred in 52.6%. Zinc deficiency occurred in 31.6%. Hypokalemia occurred in 28.6%, while hypophosphatemia was absent at admission and hypomagnesemia occurred in 4.8%.	Atypical AN in males was associated with substantial vitamin D and zinc abnormalities despite normal-range body weight.
[55]†	87 adolescent inpatients with eating disorders, mostly AN	25(OH)D, calcium, phosphorus	Mean 25(OH)D was 24.4 ± 7.4 ng/mL. About 8% were vitamin D deficient and 23% insufficient using the study's cutoffs. Calcium and phosphorus were within normal ranges. Vitamin D was lower in winter than summer.	Vitamin D insufficiency/deficiency was common, but serum calcium and phosphorus were preserved. Seasonal variation was important.

Study	Population / design	Nutrients or markers assessed	Main results relevant to micronutrient status	Overall interpretation
[56]	12 malnourished females with AN and 12 healthy controls; vitamin D2 bioavailability study	25(OH)D, vitamin D2, vitamin D3, 1,25(OH)$_2$D, calcium, phosphorus, PTH	Baseline 25(OH)D did not differ between AN and controls. Vitamin D insufficiency occurred in 17% of AN and 25% of controls. After oral vitamin D2, the 6-hour peak was lower in AN, but 24-hour and later values were similar. AN had lower baseline 1,25(OH)$_2$D, while calcium, phosphorus, and PTH were normal.	Severe malnutrition did not produce clear 25(OH)D deficiency or major impairment of oral vitamin D2 bioavailability, although active vitamin D hormone was lower.
[57]	89 female patients with untreated AN	25(OH)D, PTH, bone-related mineral markers	Severe vitamin D deficiency occurred in 16.9%, vitamin D deficiency in 19.1%, and insufficiency in 22.4%. Overall, 58.4% had 25(OH)D below 30 ng/mL. Vitamin D-deficient patients had higher PTH.	Low vitamin D status was common and was associated with endocrine changes relevant to bone metabolism.
[41]	14 severely malnourished female AN patients	Sodium, potassium, phosphate, magnesium	Hyponatremia was present in three patients and associated with hypo-osmolality and hypovolemia. Hypokalemia was present in two purging-type patients. Phosphate and magnesium abnormalities were mentioned but were not the central focus.	The study emphasizes fluid and electrolyte disturbances, especially sodium and potassium abnormalities, in severe AN.
[87]	181 adults hospitalized for severe AN	Phosphorus during refeeding	Refeeding hypophosphatemia was more common in patients with severely elevated liver enzymes: 56.8% vs 25.7% in the rest of the cohort.	Hypophosphatemia was an important refeeding-related mineral complication, especially in patients with greater medical instability.
[88]	232 adults with severe AN-BP or AN-R	Sodium, potassium, magnesium, phosphorus	AN-BP showed a stronger electrolyte-disturbance pattern. Hyponatremia occurred in 57.7% of AN-BP vs 38.5% of AN-R. Median potassium was much lower in AN-BP, 3.0 mmol/L vs 3.8 mmol/L in AN-R. Magnesium was also lower in AN-BP, while phosphorus did not differ significantly between subtypes.	Purging behavior was strongly associated with sodium, potassium, and magnesium abnormalities, whereas phosphorus was not subtype-specific at admission.
[66]	Women with active AN, remitted AN, and controls	Vitamin B12, folate, choline, betaine, methionine	Active AN did not show reduced folate or B12. Instead, vitamin B12 was elevated in active and remitted AN compared with controls, and lower BMI was associated with higher B12 in active AN. Folate did not show a clear deficiency pattern.	One-carbon metabolism markers did not follow a simple deficiency model; elevated B12 may reflect altered starvation physiology rather than nutritional adequacy.
[46]	53 adults with severe AN admitted for medical stabilization	Folate, vitamin B12, iron indices, phosphorus, indirect vitamin K status via INR	No patient had folate or B12 deficiency where measured. Iron deficiency was uncommon and did not explain most anemia. Refeeding hypophosphatemia was associated with lower initial BMI. Elevated INR in some patients suggested possible vitamin K deficiency or malabsorption, although vitamin K was not directly measured.	Severe AN-related anemia was usually not due to folate, B12, or iron deficiency. Phosphorus instability and possible vitamin K-related coagulation changes were more relevant.

Study	Population / design	Nutrients or markers assessed	Main results relevant to micronutrient status	Overall interpretation
[89]	77 underweight females with acute AN and 63 controls; reassessment after weight restoration	Total vitamin B12, holotranscobalamin, methylmalonic acid, combined B12 marker	Acute AN did not show classical B12 deficiency. Holotranscobalamin was higher in acute AN than controls. Methylmalonic acid decreased after weight restoration, and the combined B12 marker did not clearly indicate deficiency.	B12-related markers were altered but not in the direction of simple deficiency; elevations may relate to hepatic or starvation-associated physiology.
[90]	78 patients with AN in a multicentric cross-sectional study	Vitamin B12, vitamin D, folate, iron	Mean B12, vitamin D, folate, and iron were within reported reference ranges. Higher B12 was significantly associated with lower BMI. Vitamin D, folate, and iron were not significantly associated with BMI.	The study suggests that B12 may behave as a severity-associated biochemical marker, while vitamin D, folate, and iron did not clearly track BMI severity.
[91]	15 adolescents with AN and 20 matched controls	Zinc, iron, copper, magnesium, calcium	Urinary zinc excretion was significantly lower in AN, suggesting zinc conservation or depletion. Serum zinc was not significantly lower than controls. Serum iron, copper, magnesium, and calcium did not differ significantly between groups.	Zinc deficiency may be better detected through urinary zinc conservation than serum zinc alone; other measured minerals were preserved.
[92]	24 AN patients and 62 bulimia patients at admission	Serum zinc and 24-hour urinary zinc	Zinc deficiency was defined as serum zinc <70 μg/dL or urinary zinc <200 μg/24 h. Overall, 54% of AN patients met zinc-deficiency criteria. Zinc deficiency was also frequent in bulimia.	Zinc deficiency was common in AN and was detectable using combined serum and urinary criteria.
[93]	Case report of a 26-year-old woman with AN and severe dietary restriction	Zinc, potassium, calcium	Serum zinc was repeatedly below 40 μg/100 mL, indicating severe hypozincemia. The patient also had hypokalemia and hypocalcemia. Clinical signs included dermatitis, alopecia, edema, and hair discoloration.	The case illustrates severe zinc deficiency with dermatologic manifestations, alongside potassium and calcium abnormalities.
[94]	93 adolescent females with AN followed from admission to discharge and one-year follow-up	25(OH)D	At admission, 30% were vitamin D deficient. Deficiency remained similar at discharge, 29%, but increased to 45% at one-year follow-up. Mean vitamin D declined significantly over time.	Vitamin D deficiency was not universal at admission but became more frequent after follow-up, suggesting need for long-term monitoring.
[95]	14 adolescent girls with restricting-type AN followed for one year	Phosphorus, calcium, zinc, iron, ferritin, transferrin saturation, retinol-binding protein	Phosphorus remained stable and relatively high. Calcium was consistently lower than controls but within normal range. Zinc was lower than controls at six months and declined in association with weight recovery. Serum iron was generally preserved, while ferritin was high at admission and declined during recovery.	Mineral status was not uniformly deficient at admission, but zinc may become vulnerable during anabolic weight restoration.
[96]	25 patients with AN; dietary and biochemical assessment	Carotene, iron, potassium, chloride, sodium, calcium, phosphorus	Serum carotene was high-normal. Several serum biochemical markers, including iron, potassium, chloride, sodium, calcium, and phosphorus, were measured, but the study did not show a simple generalized vitamin/mineral depletion pattern.	Despite restricted intake and weight loss, serum vitamin/mineral markers were not uniformly deficient; carotene tended toward high-normal values.

Study	Population / design	Nutrients or markers assessed	Main results relevant to micronutrient status	Overall interpretation
[97]	Ten female AN patients and ten healthy controls	Zinc in serum, hair, and urine	Zinc values were higher in AN across serum, hair, and urinary compartments. Serum zinc, hair zinc, and urinary zinc excretion were all higher in AN than controls.	This study conflicts with the zinc-deficiency model and suggests that zinc metabolism in AN may vary substantially by cohort, methods, and illness stage.
[98]	Short report including 14 women admitted for inpatient treatment of severe anorexia nervosa and 10 healthy female controls of similar age	Plasma zinc, neutrophil zinc as a tissue/cellular zinc marker, albumin	Mean plasma zinc was slightly lower in AN patients than controls, 14.7 ± 2.8 μmol/L vs 16.5 ± 1.1 μmol/L, but remained within the laboratory reference range overall. Three patients had low plasma zinc values. Mean neutrophil zinc was almost identical between AN patients and controls, 1.21 ± 0.30 vs 1.20 ± 0.19 μmol/10^{10} cells, indicating no group-level tissue zinc depletion. However, 3 of 14 patients had low neutrophil zinc. Two patients had both low plasma and low neutrophil zinc, while one patient had low neutrophil zinc despite normal plasma zinc and albumin. Conversely, one patient had low plasma zinc but normal/high neutrophil zinc.	Severe anorexia nervosa was not associated with universal zinc deficiency in this study. The key contribution is methodological: plasma zinc alone was an unreliable indicator of zinc status, because some patients showed normal plasma zinc despite low neutrophil zinc, while another showed low plasma zinc despite preserved tissue zinc. The findings suggest that zinc status in AN is heterogeneous and may require cellular or functional markers in addition to plasma zinc.
[99]†	27 patients with AN or associated eating disorders; grouped by carotene status	Serum carotene, retinol, retinol-binding protein	Serum retinol and retinol-binding protein were normal across groups. Some patients showed hypercarotenemia, but this was not accompanied by vitamin A deficiency.	The study supports disturbed carotenoid handling rather than classical vitamin A deficiency.
[100]	Female AN patients and several comparison groups	Total carotene, β-carotene, retinyl esters, retinal, retinol, retinoic acid	AN was associated with increased β-carotene, retinyl esters, retinol, and retinoic acid. Retinal was not significantly different.	AN showed a paradoxical elevation of carotene and several vitamin A-related compounds rather than depletion.
[101]	17 hospitalized AN patients	Retinol, tocopherol isomers, essential fatty acids	The study focused on fat-soluble vitamins A and E and essential fatty acids. It did not measure most minerals or trace elements.	This study is useful mainly for vitamin A, vitamin E, and lipid-related nutritional status, not for broad micronutrient deficiency mapping.
[102]	20 young Swedish women with AN and 78 controls	Total 25(OH)D, free 25(OH)D, calcium, ionized calcium, phosphate, PTH	Total and free 25(OH)D did not differ significantly between AN and controls. Only two AN patients had total 25(OH)D below 50 nmol/L. Phosphate was similar between groups; total calcium was slightly higher in AN, while ionized calcium did not differ. PTH was lower in AN.	Vitamin D deficiency was not characteristic of this cohort, and calcium-phosphate homeostasis was broadly preserved.
[103]	Adolescent girls with AN compared with healthy controls	Potassium; calcium-phosphorus balance indirectly discussed	No girl with AN was hypokalemic. Mean potassium was slightly higher in AN than controls. The study did not provide direct serum concentrations of most vitamins or trace elements.	In this mostly outpatient/restricting adolescent sample, potassium deficiency was not evident, and direct micronutrient data were limited.

Study	Population / design	Nutrients or markers assessed	Main results relevant to micronutrient status	Overall interpretation
[65]	61 adolescents with AN assessed at admission and discharge	Folate, zinc, iron, sodium, potassium, calcium, phosphorus, magnesium, carotene	Serum folate deficiency increased from 21.9% at admission to 34.4% at discharge. Red blood cell folate was low in 54% at admission and remained low in many at discharge. Zinc deficiency was also important. Iron was low in 11.1% at admission and 6.3% at discharge. Sodium, potassium, calcium, magnesium, and phosphorus were generally normal, although phosphorus increased during refeeding. Some patients had elevated carotene rather than deficiency.	Folate and zinc were the main persistent micronutrient abnormalities; standard electrolytes and minerals were mostly preserved.
[104]	Case report of a 16-year-old girl with severe AN	Zinc, iron, copper, carotene, vitamin A, electrolytes	Serum zinc was low-normal but clinically interpreted as zinc-deficient because of hypogeusia, low alkaline phosphatase, and low-end zinc. Serum iron was slightly low, copper was normal, electrolytes were reported as normal. Carotene was elevated, while vitamin A was normal.	The case suggests clinically relevant zinc deficiency despite borderline serum zinc, mild iron reduction, normal copper, and hypercarotenemia without vitamin A excess or deficiency.
[105]	12 post-menarchal adolescent females with AN admitted for nutritional rehabilitation	Iron, hemoglobin, hematocrit	Iron deficiency was uncommon at admission. Only 1 of 12 patients had low serum iron. By discharge, all patients had normal serum iron, and serum iron increased significantly during refeeding without routine iron supplementation.	Iron deficiency was not a dominant biochemical feature; iron status generally normalized with nutritional rehabilitation.
[106]	33 females with restricting-type AN and 10 healthy controls	Serum zinc; IGF-I as nutritional marker	Serum zinc was significantly lower in AN than controls: 66.8 ± 3.5 μg/dL vs 82.0 ± 7.6 μg/dL. Zinc correlated positively with BMI and IGF-I.	Zinc depletion appeared linked to overall malnutrition severity rather than being an isolated abnormality.
[107]	113 women with AN and 21 healthy controls	25(OH)D3, calcium, phosphorus, ionized calcium, urinary calcium, PTH, calcium intake	Vitamin D deficiency, defined as 25(OH)D3 <20 ng/mL, occurred in 41% of AN patients. Serum calcium, phosphorus, ionized calcium, and PTH were normal on average. Calcium intake was often inadequate; 78% consumed <1200 mg/day.	Vitamin D deficiency and low calcium intake were common, but circulating calcium–phosphate homeostasis was largely preserved.
[108]	Case report of a severely cachectic woman with AN and spontaneous bacterial peritonitis	Zinc, iron indices, magnesium, phosphate, calcium, albumin	Zinc was profoundly low: 1.1 μmol/L vs normal 10–18 μmol/L. Severe microcytic anemia was present with very low transferrin saturation, but ferritin was elevated in the context of inflammation and recent iron infusion. Magnesium was slightly low, phosphate low-normal, calcium normal.	Severe zinc deficiency was the central micronutrient abnormality and may have contributed to immune and hematological vulnerability.
[51]	Case report of severe AN with starvation, laxative abuse, and refeeding	Phosphate, potassium, magnesium, calcium, sodium, chloride	Admission phosphate was low-normal at 2.7 mg/dL, while sodium, chloride, potassium, magnesium, and calcium were initially within normal ranges. Despite this, the case demonstrated major total-body phosphate depletion during refeeding.	Normal or low-normal serum phosphate may mask severe intracellular or total-body phosphate depletion before refeeding.

Study	Population / design	Nutrients or markers assessed	Main results relevant to micronutrient status	Overall interpretation
[109]	8 women with AN and 8 healthy controls; zinc absorption study	Fasting and postprandial serum zinc	Fasting serum zinc was similar in AN and controls. After a zinc-supplemented meal, the serum zinc rise was weaker in AN.	Zinc metabolism may be abnormal even when fasting serum zinc appears normal; impaired absorption or altered zinc handling is possible.
[110]	Case report of a 19-year-old woman with severe AN, vegetarian restriction, overexercise, and scurvy-like features	Vitamin C, vitamin B12, folate, calcium, ferritin, electrolytes	Vitamin C deficiency was documented by a saturation test and clinical response to ascorbic acid. Vitamin B12 was elevated, folate normal, calcium normal, ferritin normal, and routine electrolytes normal.	Severe vitamin C deficiency can occur in AN even when some fruit/vegetable intake is present, particularly when total intake is extremely low.
[111]	28 female AN patients compared with constitutionally thin and normal-weight controls	Potassium, sodium, urinary potassium, urinary sodium	Serum potassium and sodium were normal, but 24-hour urinary potassium excretion was significantly lower in AN than both comparison groups. Urinary sodium was similar across groups.	AN may involve reduced total-body potassium pool despite normal serum potassium.
[112]	30 hospitalized female AN patients	Zinc, copper, iron, TIBC, carotene, vitamin A, retinol-binding protein, folate	Plasma zinc and urinary zinc were reduced. Plasma copper was also reduced. Serum iron was preserved, but TIBC was reduced. Carotene was elevated, while vitamin A and retinol-binding protein were normal. Folate was variable but not uniformly deficient.	The pattern was selective trace-metal depletion with preserved or elevated vitamin-related markers rather than global micronutrient collapse.
[113]	24 female AN patients admitted to hospital	Plasma zinc, plasma copper, hair zinc/copper, carotene, vitamin A, RBP, ceruloplasmin	Plasma zinc and copper were significantly lower than controls. Hair zinc was normal on average, while hair copper was increased. Carotene was elevated, but vitamin A and RBP were not significantly reduced.	Circulating trace elements were reduced, especially zinc and copper, while vitamin A transport appeared preserved and carotene was elevated.
[114]	13 young AN patients, including males and females aged around 10–11 years	Copper, zinc, iron, albumin, hematocrit, red blood cells	Copper deficiency occurred in 46% of patients. Mean zinc remained within the normal range. Mean serum iron was substantially low, with reduced hematocrit and red blood cell count.	Copper and iron abnormalities were prominent, while zinc was relatively preserved in this young AN cohort.
[115]	18 AN patients compared with 20 controls	Vitamins A, D, E, C, folate, zinc, copper, ceruloplasmin	Vitamins A, D, E, C, and folate were generally preserved. Serum zinc and copper were significantly lower in AN, and ceruloplasmin was also reduced.	Severe wasting coexisted with preserved vitamin indices but selective depression of zinc, copper, and ceruloplasmin.
[116]	27 adolescents with restrictive AN and 11 controls; iron-metabolism study	Iron, ferritin, hepcidin, vitamin B12, folate, hemoglobin	Vitamin B12 and folate were generally normal. Serum iron was preserved in most patients. Ferritin and hepcidin were elevated despite severe undernutrition.	Iron status did not show a simple deficiency profile; amenorrhea and altered iron regulation may preserve or elevate iron stores.
[63]	32 hospitalized women with eating disorders, including 8 with AN and 24 with BN	Vitamins A, E, C, B1, B2, B6, folate, B12, indirect vitamin K via prothrombin time	Plasma tocopherol, retinol, folate, and cobalamin were mostly within reference ranges. Vitamin C was reduced in several patients. Functional tests suggested B2 deficiency in some patients and possible B1/B6 abnormalities. Folic acid intake was low in most patients. Prolonged prothrombin time in five patients suggested possible vitamin K deficiency.	Plasma vitamin levels were often normal despite low intake, but functional B-vitamin abnormalities, vitamin C reduction, low folate intake, and possible vitamin K disturbance were relevant.

Study	Population / design	Nutrients or markers assessed	Main results relevant to micronutrient status	Overall interpretation
[117]	84 female outpatients with AN, divided into mild vs moderate-to-extreme severity	Vitamin D, vitamin B12, folate, iron	Mean vitamin D was 24.69 ± 8.58 ng/mL and did not differ significantly by severity. Vitamin B12 tended to be higher in more severe AN, but not significantly. Folate and iron did not differ significantly by severity.	The biochemical profile did not show progressive depletion with lower BMI; vitamin D was borderline/low on average, while B12 tended to rise with severity.
[118]	19 adolescent girls with AN and 19 healthy controls	Calcium intake/status, phosphorus, ionized calcium, urinary calcium, vitamin D-related markers	Serum ionized calcium and phosphorus were normal in all subjects. Urinary calcium was generally normal, although calcium intake and bone-related nutrient adequacy were concerns. No overt hypocalcemia, hypophosphatemia, abnormal PTH, or clear generalized vitamin D deficiency explained skeletal findings.	Bone abnormalities were not explained by simple calcium–phosphate or vitamin D biochemical deficiency, despite nutritional depletion.
[119]	25 young women with AN undergoing 12-week intensive nutritional rehabilitation	25(OH)D, calcium, phosphate	Median 25(OH)D was 84.4 nmol/L at baseline. Only 1 patient was vitamin D deficient and 2 had levels between 25 and 50 nmol/L. Calcium intake during treatment was high, and serum calcium and phosphate remained stable and normal.	Vitamin D status was mostly adequate, likely influenced by supplementation, and calcium–phosphate homeostasis remained stable during monitored refeeding.
[120]	73 postpubertal female AN patients across active, weight-recovered amenorrheic, and fully recovered stages	Calcium excretion, 25(OH)D, 1,25$(OH)_2$D, PTH-related markers	Active AN showed elevated urinary calcium excretion, suggesting unfavorable calcium handling. Low 25(OH)D occurred in 24.6% overall: 17.6% in active AN, 35% in weight-recovered amenorrheic patients, and 26.3% in fully recovered patients. 1,25$(OH)_2$D was generally normal and increased with recovery.	Vitamin D insufficiency affected about one-quarter of patients, and calcium handling improved with nutritional and endocrine recovery.
[121]	Case report of a 48-year-old woman with chronic restricting-type AN and osteomalacia	Vitamin D, calcium, phosphate, PTH, bone-turnover markers	Profound vitamin D deficiency caused osteomalacia, bone pain, fractures, hypocalcemia, secondary hyperparathyroidism, and high bone turnover. Dietary intake was extremely restricted and low in fat-containing vitamin D sources.	This case illustrates severe vitamin D deficiency with clinically overt skeletal consequences in chronic AN.
[122]	Case report of longstanding AN with laxative and diuretic abuse	Magnesium, calcium, potassium, phosphate	Severe hypomagnesemia was central and maintained refractory hypocalcemia and hypokalemia. Admission potassium was extremely low at 1.4 mmol/L. Mineral/electrolyte correction required magnesium replacement.	Magnesium deficiency can drive persistent calcium and potassium abnormalities in AN, especially with purging or diuretic/laxative abuse.
[123]	Case report of a 16-year-old girl with severe AN, starvation, laxative abuse, and binge eating	Phosphate, potassium, magnesium and refeeding-related electrolytes	The dominant abnormality was acute phosphate disturbance after sudden binge eating/refeeding in the context of severe starvation and likely depleted intracellular stores.	Severe hypophosphatemia can be triggered not only by formal refeeding protocols but also by sudden uncontrolled carbohydrate/energy intake after starvation.

Study	Population / design	Nutrients or markers assessed	Main results relevant to micronutrient status	Overall interpretation
[124]	61 newly diagnosed adolescents with AN	Vitamin D intake, calcium intake, 25(OH)D, calcium, phosphate, magnesium, PTH, albumin, urinary calcium	Only 1 patient, or 2%, was vitamin D deficient; 13 patients, or 25%, were insufficient; and 39, or 74%, were sufficient. Dietary vitamin D intake was generally low. Calcium status was assessed biochemically and by intake, with serum values often preserved.	Biochemical vitamin D deficiency was uncommon, but vitamin D intake was low and serum sufficiency did not necessarily indicate optimal habitual intake.
[125]	Women with AN and reduced bone density	Calcium intake, urinary calcium, serum calcium, phosphate, vitamin D/PTH-related markers	Dietary calcium intake and urinary calcium excretion were low, but serum calcium and inorganic phosphate were normal. Bone loss was not explained by frank hypocalcemia, hypophosphatemia, or classical vitamin D deficiency.	Normal serum calcium/phosphate did not exclude poor calcium nutrition or skeletal mineral compromise.
[126]	29 women with AN, some with bulimic symptoms, compared with controls	Calcium, phosphorus, potassium, sodium, vitamin D metabolites, PTH-related markers	Serum calcium and inorganic phosphorus were normal. Alkaline phosphatase was normal on average. Sodium and potassium were assessed as routine electrolytes. The study did not demonstrate overt hypocalcemia or hypophosphatemia.	Reduced bone density occurred despite largely preserved circulating calcium–phosphate indices.
[127]	214 community-dwelling women with AN	Potassium, sodium, calcium-related information, albumin, hematological indices	Hypokalemia occurred in about 20% of participants, with the lowest potassium reported as 1.9 mEq/L. Hyponatremia occurred in 7%, with one seizure history related to hyponatremia. Calcium-related data were indirect and not a full vitamin/mineral panel.	Electrolyte abnormalities, especially potassium and sodium disturbances, were clinically important even in community-dwelling AN patients.
[128]†	13 patients with eating disorders, mostly with AN history	Thiamin/B1, riboflavin/B2, vitamin B6, albumin, clinical chemistry	No biochemical evidence of thiamin deficiency was detected. Riboflavin deficiency markers were present in 31%, and vitamin B6 deficiency markers in 31%.	Functional B-vitamin testing showed that B2 and B6 insufficiency may be common despite absence of overt clinical deficiency.
[64]	17 adolescent girls with AN assessed before and after nutritional rehabilitation	Riboflavin/B2, FMN, FAD, urinary organic acids, protein-status markers	Riboflavin metabolism was abnormal. Patients did not show simple low riboflavin, but rather altered distribution of riboflavin forms, including high erythrocyte free riboflavin and reduced metabolically important FAD.	AN may disturb riboflavin metabolism and cofactor conversion, not merely riboflavin intake.
[129]	17 AN patients with low calcium and vitamin D intake	25(OH)D, 1,25(OH)$_2$D, calcium, phosphate, PTH, bone markers	Dietary calcium and vitamin D intake were extremely low. Median 25(OH)D was 8 nmol/L in AN vs 38 nmol/L in controls. 15 of 17 patients, or 88%, had 25(OH)D below the normal range. 1,25(OH)$_2$D was usually preserved.	This study showed profound vitamin D depletion despite relative preservation of active vitamin D in most patients.
[130]	49 young women with AN and 24 controls	Calcium intake, 25(OH)D, calcium–phosphorus metabolism, urinary calcium/phosphorus	25(OH)D was significantly lower in AN than controls, but remained within the authors' normal range. Ionized calcium, urinary calcium, and urinary phosphorus were not significantly different from controls.	AN was associated with relatively reduced vitamin D and calcium intake/status, but not overt calcium–phosphate biochemical collapse.

Study	Population / design	Nutrients or markers assessed	Main results relevant to micronutrient status	Overall interpretation
[131]	3-year follow-up of young women with AN after intensive nutritional therapy	Total 25(OH)D, free 25(OH)D, calcium, phosphate, PTH/n-oxPTH	Total 25(OH)D was mostly adequate; only one patient was deficient and one insufficient. Free 25(OH)D declined over time. Serum calcium remained relatively preserved and phosphate was stable from baseline to 3 years.	Total vitamin D may appear sufficient, but free vitamin D may reveal longer-term alterations relevant to bone metabolism.
[132]	30 patients with AN who underwent DXA scanning	25-hydroxyvitamin D, cobalamin/B12, ionized calcium	Mean 25(OH)D was within the reference range, but 20% had values below 50 nmol/L. Vitamin D declined with longer illness duration: patients with disease duration >4 years had the lowest mean 25(OH)D. Mean cobalamin was within the normal range. Ionized calcium was normal.	Vitamin D insufficiency was present in a minority and appeared more pronounced with longer illness duration; B12 and ionized calcium were generally preserved.
[133]	91 female AN patients with secondary amenorrhea completing a 20-week weight-restoration program	25(OH)D, calcium/vitamin D–bone-related markers	Mean 25(OH)D decreased significantly during treatment, from 27.0 ± 14.0 ng/mL to 22.5 ± 11.6 ng/mL. Patients who reached post-treatment 25(OH)D ≥30 ng/mL showed better skeletal response to weight gain.	Weight restoration alone did not correct vitamin D status; vitamin D may decline during recovery and may need active monitoring/supplementation.
[134]	Prospective 1-year study of adolescents/young women with severe AN	25(OH)D, PTH, urinary calcium, calcium intake	Mean baseline 25(OH)D was normal, but several patients had low levels. Mean 25(OH)D decreased at 6 months before returning toward baseline at 12 months. Calcium–vitamin D–PTH physiology was evaluated mainly in relation to skeletal recovery.	Mean vitamin D values may appear acceptable, but individual low values and temporal decline during recovery are clinically relevant.
[58]	50 female AN patients and 200 healthy controls	25(OH)D, calcium, phosphorus, supplementation behavior	Vitamin D deficiency was rare in AN: only 2% had 25(OH)D <20 ng/mL. Suboptimal vitamin D <30 ng/mL occurred in 22% of AN patients. Calcium and phosphorus were similar to controls. Preserved vitamin D status appeared partly related to supplementation and sample characteristics.	AN did not show higher vitamin D deficiency than controls in this cohort, likely because of high supplement use and demographic factors.
[135]	9 women with AN studied before and after oral refeeding	25(OH)D, calcium, phosphorus, total proteins	25(OH)D remained within the laboratory reference range before and after weight gain. Serum calcium and phosphorus were also within normal ranges.	Bone and metabolic improvement after refeeding was not explained by correction of vitamin D, calcium, or phosphorus deficiency.
[136]	12 pubertal girls with relapsing AN and healthy pubertal controls	25(OH)D, $1,25(OH)_2D$, $24,25(OH)_2D$, vitamin D-binding protein, calcium, phosphate	25(OH)D was normal and similar to controls, but active $1,25(OH)_2D$ was significantly reduced. $24,25(OH)_2D$ was increased. Calcium, phosphate, alkaline phosphatase, and albumin were within normal values.	Vitamin D stores were preserved, but vitamin D metabolism was altered, with reduced active vitamin D despite normal calcium–phosphate markers.

Study	Population / design	Nutrients or markers assessed	Main results relevant to micronutrient status	Overall interpretation
[137]	565 hospitalized adolescents and young adults with eating disorders, including 332 with AN	25(OH)D, calcium, phosphorus	Mean 25(OH)D was 32.15 ± 11.87 ng/mL. Vitamin D insufficiency occurred in 43.0%, deficiency in 12.4%, and severe deficiency in 3.0%. Severe deficiency was more common in males. Calcium was lower in males than females and positively associated with vitamin D. Phosphorus did not differ by sex and was not associated with vitamin D.	Vitamin D insufficiency was very common in medically hospitalized eating-disorder patients, with males showing greater risk of severe deficiency.
128]	Women with AN undergoing intensive nutritional rehabilitation; healthy controls	25(OH)D, calcium intake, multivitamin use, potassium correction	Mean 25(OH)D was not significantly different from controls. All patients had 25(OH)D above 12 ng/mL, while 13% had levels below 20 ng/mL.	Severe skeletal impairment in AN was not primarily explained by overt vitamin D deficiency in this cohort.
[139]	44 adolescent girls with AN and 25 healthy controls	25(OH)D, calcium intake, vitamin D intake	Girls with AN had higher 25(OH)D than controls: 30.2 ± 1.4 vs 22.2 ± 1.1 ng/mL. They also had higher calcium and vitamin D intake, but lower bone density and altered bone markers.	Vitamin D status was not deficient and did not protect against skeletal impairment, suggesting that undernutrition/endocrine suppression were more central.
[140]	21 adolescent girls/young women with AN and 23 controls	25(OH)D	25(OH)D was significantly higher in AN than controls: 43.6 ± 3.9 vs 31.8 ± 3.6 ng/mL. Despite higher vitamin D, AN patients had poorer bone outcomes.	Vitamin D deficiency was not evident; skeletal impairment occurred despite preserved or elevated 25(OH)D.
[141]	30 women with AN, 19 with hypothalamic amenorrhea, and 30 healthy controls	25(OH)D, vitamin D intake, calcium intake, ionized calcium, PTH, urinary calcium	25(OH)D was within normal range in all groups, though lower in AN than hypothalamic amenorrhea. Calcium intake was similar or numerically higher in AN, partly due to supplementation. Ionized calcium, PTH, and urinary calcium were similar across groups.	Osteopenia in AN was not explained by frank vitamin D or calcium deficiency, but by broader undernutrition, low lean/fat mass, and low IGF-I.
[142]	12 female AN patients and controls; vitamin D metabolism study	25(OH)D, $1,25(OH)_2D$, vitamin D-binding protein, calcium, phosphate, urinary calcium	25(OH)D was normal and similar to controls. Active $1,25(OH)_2D$ was significantly reduced. Vitamin D-binding protein concentration was normal, but serum binding capacity was reduced. Calcium was normal and phosphate high-normal. Urinary calcium interpretation depended on correction method.	AN showed altered vitamin D metabolism/transport rather than classical vitamin D deficiency.
[143]	46 women with AN during inpatient treatment	Total 25(OH)D2/25(OH)D3	Mean vitamin D increased from 28.57 ± 11.90 ng/mL at admission to 44.35 ± 15.31 ng/mL at discharge. Patients with insufficient baseline levels received cholecalciferol.	Vitamin D status improved substantially during treatment, likely reflecting supplementation and clinical monitoring rather than spontaneous repletion alone.
[144]	63 Japanese female AN patients, restrictive and binge/purge subtypes	25(OH)D, intact PTH	Only 14% had vitamin D sufficiency >30 ng/mL. About 86% had insufficiency or deficiency, and approximately 71% had frank deficiency below 20 ng/mL.	Vitamin D deficiency was highly prevalent and represented a dominant micronutrient abnormality in this Japanese AN cohort.
[145]	12 female AN patients compared with controls; renal/osmoregulation study	Sodium, potassium, chloride, calcium, osmolality-related markers	Sodium, chloride, and plasma osmolality were significantly lower than controls but generally remained within normal laboratory ranges. Potassium was normal and stable. No major chronic hypokalemia was documented.	The study suggests mild sodium–chloride/osmoregulatory alterations rather than overt potassium or broad electrolyte depletion.

Study	Population / design	Nutrients or markers assessed	Main results relevant to micronutrient status	Overall interpretation
[43]	7 female adolescents with AN and 7 controls; calcium kinetics study	Calcium balance, urinary calcium, bone-derived calcium, calcium deposition	AN patients had marked hypercalciuria. Urinary calcium was mainly bone-derived rather than diet-derived. Bone calcium deposition and exchangeable bone calcium pool were reduced.	AN was associated with disrupted skeletal calcium handling, with urinary calcium loss reflecting bone mineral loss rather than high dietary calcium absorption.
[146]	19 adolescent female inpatients with AN during 11-week treatment	Calcium, phosphate, calcium supplementation	Calcium intake was increased to approximately 2000 mg/day. Serum calcium remained normal and stable. Serum phosphate also remained stable and within reference range during treatment.	Controlled refeeding with calcium support maintained serum calcium and phosphate; bone recovery was not due to correction of frank hypocalcemia or hypophosphatemia.
[147]	90 women with eating disorders, including 22 with AN, compared with controls	Sodium, potassium, chloride, total CO_2, calcium, phosphate, magnesium	Eating-disorder patients had significantly lower potassium, chloride, and phosphate than controls. Calcium was higher, partly explained by albumin and total CO_2. Magnesium did not differ significantly at group level, though individual abnormalities occurred.	Electrolyte and phosphate abnormalities were prominent, especially in relation to purging/metabolic disturbance, while magnesium was less consistently abnormal.
[148]	Case report of a 35-year-old woman with extremely severe chronic AN	Phosphorus, calcium, potassium, sodium, chloride	Admission phosphorus was normal at 2.7 mg/dL despite extreme malnutrition, but phosphorus dysregulation during refeeding was central. Ionized calcium was normal; total calcium was low-normal/slightly reduced. Potassium was borderline/low early but stabilized. Sodium and chloride remained broadly normal.	Severe total-body phosphorus depletion may be hidden by normal admission phosphate and become clinically important during refeeding.
[149]	Case report of a 19-year-old male with AN after carbohydrate-rich binge/refeeding	Phosphate, zinc, sodium, potassium, calcium, magnesium, folate, B12	Marked hypophosphatemia was present at admission: phosphate 0.2 mmol/L. Zinc was mildly low. Sodium, potassium, calcium, and magnesium were normal. Folate and B12 were within reference ranges.	The case illustrates selective, severe phosphate collapse after sudden carbohydrate intake, with only mild zinc deficiency and preserved other measured vitamins/electrolytes.
[150]	Case series of 5 adolescent/young adult females with AN	Phosphate, potassium, sodium, magnesium, calcium	Severe hypophosphatemia was the dominant abnormality and often developed or worsened during treatment. Several patients also had hypokalemia and major sodium disturbances, including severe hyponatremia and later hypernatremia in one case. Hypophosphatemia was linked with delirium, seizures, arrhythmia, and fatal cardiac outcome in one patient.	Phosphate monitoring is critical in severe AN; dangerous complications may occur even when attention is focused mainly on potassium.
[151]	50 adult AN patients undergoing supervised refeeding	Serum magnesium	Low magnesium occurred in 60% of patients during hospitalization. Some had hypomagnesemia at admission, while others developed it later during refeeding. Mean admission magnesium was 0.79 ± 0.09 mmol/L; those hypomagnesemic at admission averaged 0.64 ± 0.05 mmol/L.	Magnesium deficiency may be common during refeeding and can be missed if only admission values are considered.

Study	Population / design	Nutrients or markers assessed	Main results relevant to micronutrient status	Overall interpretation
[152]	70 female adolescent inpatients with restrictive-type AN	Vitamin B12, folate	Mean vitamin B12 was high-normal/elevated: 643 ± 333 pg/mL. 38.5% had B12 above the upper reference limit, and none had low B12. Folate deficiency was rare: 1.4% below reference range; 4.3% above range.	B12 deficiency was absent, while hypercobalaminemia was frequent and may reflect liver involvement or starvation physiology rather than nutritional adequacy.
[153]	Case report of a 30-year-old woman with AN and sensory neuronopathy	Functional vitamin B12 status, folate, vitamin E	Serum B12 remained within the laboratory range, but functional B12 deficiency was suggested by hyperhomocysteinemia and macrocytosis. Folate was low at baseline. Vitamin E was normal.	Normal serum B12 may miss clinically significant functional B12 deficiency; folate deficiency may coexist.
[52]†	113 patients with eating disorders in primary care, including 68 with AN	Red cell folate, B12, vitamin D, magnesium, manganese, zinc, calcium, potassium, sodium, phosphate, ferritin, albumin, cholesterol	Red cell folate and B12 were often elevated rather than deficient. Vitamin D was reduced. Manganese was markedly low in many patients; zinc and magnesium showed group-level downward shifts. Potassium and sodium were reduced in some patients. Phosphate was elevated rather than reduced at first measurement.	This broad panel showed heterogeneous abnormalities: not global depletion, but reduced vitamin D, manganese, zinc, magnesium, potassium/sodium shifts, and paradoxically elevated folate/B12/phosphate in some contexts.
[67]	71 Japanese female AN patients assessed before nutritional therapy	Vitamin B12	B12 deficiency was uncommon: 2 of 71 patients had low B12. Elevated B12 was relatively frequent: 22 of 71 patients had values above the reference range.	B12 status in AN may be paradoxically elevated; low B12 was rare in this pre-treatment cohort.
[154]	112 AN patients, mostly adolescent/young adult females	Serum cobalamin/B12	The study focused narrowly on B12. Patients were severely underweight, but B12 abnormality was not presented as simple deficiency; B12 status varied by BMI severity and was evaluated as a marker rather than a broad nutritional panel.	This study supports the idea that B12 should be interpreted cautiously in AN and not assumed to be low purely because BMI is low.
[155]	64 active AN, 49 remitted AN, and 49 controls	Plasma vitamin B12, betaine	Plasma B12 was significantly higher in active AN and remitted AN than controls. Active AN mean B12 was 571.0 ± 505.2; remitted AN 588.2 ± 379.9; controls 339.6 ± 224.3.	One-carbon-related nutrient markers again showed paradoxical B12 elevation rather than depletion.
[156]	Case report of severe chronic AN treated with parenteral nutrition	Selenium, glutathione peroxidase-related markers	Serum selenium was profoundly low: 13 µg/L vs reference 107–171 µg/L. Selenium deficiency emerged with muscle pain/weakness during parenteral nutrition and was accompanied by reduced selenium-dependent antioxidant activity.	The case shows that caloric/parenteral repletion without adequate selenium can precipitate clinically significant selenium-deficiency myopathy.
[68]	Case report of a 16-year-old girl with restrictive-type AN and scurvy	Vitamin C, vitamin A, vitamin B12, refeeding-related markers	Serum vitamin C was low: 21 µmol/L, below the reference range of 25–114 µmol/L, confirming scurvy. Vitamin A and B12 were normal. The paper did not provide a broad micronutrient panel.	Severe vitamin C deficiency can occur in restrictive AN, even when other measured vitamins such as A and B12 are normal.

Study	Population / design	Nutrients or markers assessed	Main results relevant to micronutrient status	Overall interpretation
[69]	Case report of a 14-year-old non-binary youth assigned female at birth with severe AN-R and extreme restriction	Vitamin C, potassium	The dominant micronutrient abnormality was clinically diagnosed vitamin C deficiency/scurvy. The patient had perifollicular petechiae, diffuse petechiae, and ecchymoses after months of very low intake of vitamin C-rich foods. Plasma vitamin C was not measured before supplementation, but the clinical picture and response to vitamin C supported the diagnosis. Mild hypokalemia was also present, with potassium of 3.0 mmol/L.	Severe restrictive AN can produce clinically evident scurvy, even when diagnosis is based on clinical signs rather than pre-treatment vitamin C measurement. Mild hypokalemia may coexist in severe restriction without purging.
[157]	20 women with current AN, 16 weight-restored AN, and 18 healthy controls; QSM MRI study	Brain iron content estimated by quantitative susceptibility mapping	Whole-brain and ROI-based QSM analyses found no significant group differences in brain iron estimates between current AN, weight-restored AN, and controls. Exploratory correlations suggested that lower BMI, greater illness severity, longer illness duration, and earlier onset may relate to higher regional QSM values in limbic/subcortical areas.	The study did not support generalized brain iron deficiency or overload in AN, but suggested possible region-specific iron associations with illness severity and chronicity.
[158]	Case report of a young woman with severe restrictive AN and extreme hyperferritinemia	Ferritin, serum iron, transferrin, transferrin saturation, vitamin B12, folate	Ferritin was extremely elevated at admission, 9096 µg/L, despite low serum iron and low-end transferrin saturation. Vitamin B12 and folate were elevated or sufficient rather than deficient.	Severe AN may involve marked disturbance of iron handling, with hyperferritinemia despite reduced circulating iron availability. This pattern should not be interpreted as simple iron excess or classical iron deficiency.
[159]	Retrospective study of eating-disorder patients with ferritin data; AN compared with other eating disorders	Ferritin, serum iron, transferrin saturation, TIBC, hemoglobin	Classical iron deficiency occurred in 16% of AN patients, not significantly different from 19% in non-AN eating disorders. Hemoglobin, plasma iron, and transferrin saturation were broadly similar between groups, but TIBC was significantly lower in AN. Ferritin tended to be higher in AN and may be influenced by starvation-related mechanisms.	Iron deficiency was present only in a minority. AN showed a complex iron profile, with altered ferritin and TIBC rather than simple depletion of iron stores.
[160]	Case report of a 23-year-old woman with extreme restrictive AN during refeeding	Phosphorus, ferritin, serum iron, iron-binding capacity, vitamin B12, folate, B-vitamin supplementation	Admission iron was high-normal/elevated and ferritin markedly elevated. B12 and folate were measured but were not clearly identified as deficient. Severe hypophosphatemia developed during refeeding, with phosphorus falling to <0.7 mg/dL and coinciding with worsening anemia and leukopenia. Blood counts improved after phosphate replacement.	The central micronutrient/electrolyte event was severe refeeding hypophosphatemia, which may contribute to hematological deterioration. Iron markers were paradoxically elevated rather than depleted.
[161]	53 adolescents with AN followed during treatment	Ferritin, hemoglobin, MCV, serum iron, transferrin saturation, inflammatory markers	No patient developed anemia. Hemoglobin, MCV, serum iron, and transferrin saturation were generally normal. Elevated ferritin in some patients was not accompanied by a pattern of iron deficiency or inflammation.	Ferritin may behave as a nutritional/starvation-related marker in adolescent AN, but elevated ferritin does not necessarily indicate iron overload or inflammation.

Study	Population / design	Nutrients or markers assessed	Main results relevant to micronutrient status	Overall interpretation
[53]†	Women with eating disorders, including AN, BN, and atypical ED; outpatient clinic study	Vitamins A, E, B6, zinc, copper	Vitamin A and vitamin E were higher in eating-disorder patients than controls, especially among supplement users. Non-supplemented eating-disorder patients did not show significantly lower B6, zinc, or copper than controls. Low body weight predicted poorer B6 functional status.	In this mixed outpatient sample, fat-soluble vitamin deficiency was not typical; vitamins A and E were often elevated. Zinc and copper were generally preserved, while low body weight may increase risk of functional B6 insufficiency.
[61]	37 patients with AN and blood donor controls	Vitamin B1/thiamin status by erythrocyte transketolase activation	Mean erythrocyte transketolase activation was much higher in AN than controls, indicating poorer thiamin-dependent enzyme saturation. Using >12.6% activation, 38% of AN patients showed biochemical thiamin deficiency; using >32%, 19% still met deficiency criteria.	Functional thiamin deficiency may be relatively common in AN when assessed with sensitive erythrocyte enzyme testing.
[62]	24 adults with extreme AN, mean BMI 12.54 kg/m²; controls	Whole-blood thiamine diphosphate/TDP	Mean admission TDP was within the reference range and did not differ significantly from controls. No participant showed clinical concern for Wernicke's encephalopathy. AN-R and AN-BP subgroup means were also within range.	This study conflicts with the idea of frequent baseline thiamine deficiency in extreme AN, although early thiamine dosing before sampling may have influenced results.
[59]	82 female adolescents with AN; antioxidant micronutrient study	Vitamin E/erythrocyte α-tocopherol; antioxidant enzyme activities	Erythrocyte tocopherol was significantly lower in AN than reference values. About 21% of patients had tocopherol below the reference interval. Antioxidant enzyme systems were also assessed.	A subgroup of adolescents with AN may have vitamin E/antioxidant micronutrient insufficiency, potentially reflecting low intake, oxidative stress, or both.
[162]	15 AN and 18 BN patients compared with healthy volunteers; zinc supplementation study	Serum zinc, 24-hour urinary zinc, dietary zinc intake, copper safety monitoring	Mean serum zinc in AN was similar to controls, but urinary zinc excretion was significantly lower in AN, suggesting zinc conservation/depletion. Some individual AN patients were hypozincemic.	Zinc deficiency in AN may be more visible through urinary zinc conservation than serum zinc alone. Serum zinc may underestimate functional or total-body zinc vulnerability.
[163]	43 female adolescents with AN at diagnosis	Serum folate, serum cobalamin/B12, homocysteine, methylmalonic acid	Serum folate was generally normal, but one patient had low folate and the highest homocysteine. About 21% had low-normal folate with elevated homocysteine, suggesting possible functional folate insufficiency. Cobalamin was not reduced, and methylmalonic acid was normal where measured.	Folate-related one-carbon metabolism may be subtly impaired in some adolescents with AN even when serum folate is technically normal; B12 deficiency was not supported.
[164]	18 women with AN, 27 with BN, and 25 controls	Serum folate, vitamin B12	Folate and B12 did not differ significantly between AN, BN, and controls. Mean folate was 13.8 ng/mL in AN, 13.9 ng/mL in BN, and 10.5 ng/mL in controls. Mean B12 was 404.4 pg/mL in AN, 362.9 pg/mL in BN, and 374.0 pg/mL in controls.	This study found no evidence of group-level folate or B12 deficiency in AN.
[165]	70 female eating-disorder patients, including adolescent and adult AN subgroups	Vitamin B12, folate, homocysteine	Vitamin B12 and folate were generally within reference ranges. Adult AN patients had higher B12 than BN and ED-NOS patients, while folate did not differ meaningfully across adult diagnostic groups.	B12 and folate deficiency were not typical; adult AN may show relatively higher B12 compared with other eating-disorder groups.

Study	Population / design	Nutrients or markers assessed	Main results relevant to micronutrient status	Overall interpretation
[166]	11 female AN patients and 12 healthy female controls	Folic acid, vitamin B6, vitamin B12	AN patients were not deficient in folic acid, B6, or B12. Vitamin B6 was significantly higher in AN than controls, and B12 tended to be higher. Folic acid was comparable between groups.	The study does not support B-vitamin depletion; instead, B6 and possibly B12 were elevated in AN.
[54]	49 female adolescents with eating disorders, including 23 with AN; serum carotene analyzed in 46	Serum carotene	Eating-disorder patients had higher serum carotene than comparison groups. Within eating disorders, AN had the highest mean carotene. Hypercarotenemia occurred in 4 of 21 tested AN patients and in 13% of the total eating-disorder sample.	Hypercarotenemia may occur in adolescent AN, but it is not universal; it has high specificity but low sensitivity for eating disorders in this sample.

Note: Entries are descriptive and may overlap in biological markers, outcomes or clinical timing. Individual reference details are provided in the main article; source categories are explained in Section 2.8.
